# Fulvestrant plus capivasertib versus placebo after relapse or progression on an aromatase inhibitor in metastatic, oestrogen receptor-positive breast cancer (FAKTION): a multicentre, randomised, controlled, phase 2 trial

**DOI:** 10.1016/S1470-2045(19)30817-4

**Published:** 2020-03

**Authors:** Robert H Jones, Angela Casbard, Margherita Carucci, Catrin Cox, Rachel Butler, Fouad Alchami, Tracie-Ann Madden, Catherine Bale, Pavel Bezecny, Johnathan Joffe, Sarah Moon, Chris Twelves, Ramachandran Venkitaraman, Simon Waters, Andrew Foxley, Sacha J Howell

**Affiliations:** a Department of Cancer and Genetics, Cardiff University, Cardiff, UK; b Centre for Trials Research, Cardiff University, Cardiff, UK; c Velindre Cancer Centre, Cardiff, UK; d All Wales Laboratory Genetics Service, University Hospital of Wales, Cardiff, UK; e Department of Cellular Pathology, University Hospital of Wales, Cardiff, UK; f Betsi Cadwaladr University Health Board, Bangor, UK; g Blackpool Teaching Hospitals NHS Foundation Trust, Blackpool, UK; h Calderdale & Huddersfield NHS Foundation Trust, Huddersfield, UK; i University Hospitals of Morecambe Bay NHS Foundation Trust, Lancaster, UK; j University of Leeds and Leeds Teaching Hospitals Trust, Leeds, UK; k The Ipswich Hospital NHS Trust, Ipswich, UK; l Oncology R&D, AstraZeneca, Cambridge, UK; m Division of Cancer Sciences, The University of Manchester and The Christie NHS Foundation Trust, Manchester, UK

## Abstract

**Background:**

Capivasertib (AZD5363) is a potent selective oral inhibitor of all three isoforms of the serine/threonine kinase AKT. The FAKTION trial investigated whether the addition of capivasertib to fulvestrant improved progression-free survival in patients with aromatase inhibitor-resistant advanced breast cancer.

**Methods:**

In this randomised, double-blind, placebo-controlled, phase 2 trial, postmenopausal women aged at least 18 years with an Eastern Cooperative Oncology Group performance status of 0–2 and oestrogen receptor-positive, HER2-negative, metastatic or locally advanced inoperable breast cancer who had relapsed or progressed on an aromatase inhibitor were recruited from 19 hospitals in the UK. Enrolled participants were randomly assigned (1:1) to receive intramuscular fulvestrant 500 mg (day 1) every 28 days (plus a loading dose on day 15 of cycle 1) with either capivasertib 400 mg or matching placebo, orally twice daily on an intermittent weekly schedule of 4 days on and 3 days off (starting on cycle 1 day 15) until disease progression, unacceptable toxicity, loss to follow-up, or withdrawal of consent. Treatment allocation was done using an interactive web-response system using a minimisation method (with a 20% random element) and the following minimisation factors: measurable or non-measurable disease, primary or secondary aromatase inhibitor resistance, *PIK3CA* status, and PTEN status. The primary endpoint was progression-free survival with a one-sided alpha of 0·20. Analyses were done by intention to treat. Recruitment is complete, and the trial is in follow-up. This trial is registered with ClinicalTrials.gov, number NCT01992952.

**Findings:**

Between March 16, 2015, and March 6, 2018, 183 patients were screened for eligibility, of whom 140 (76%) were eligible and were randomly assigned to receive fulvestrant plus capivasertib (n=69) or fulvestrant plus placebo (n=71). Median follow-up for progression-free survival was 4·9 months (IQR 1·6–11·6). At the time of primary analysis for progression-free survival (Jan 30, 2019), 112 progression-free survival events had occurred, 49 (71%) in 69 patients in the capivasertib group compared with 63 (89%) of 71 in the placebo group. Median progression-free survival was 10·3 months (95% CI 5·0–13·2) in the capivasertib group versus 4·8 months (3·1–7·7) in the placebo group, giving an unadjusted hazard ratio (HR) of 0·58 (95% CI 0·39–0·84) in favour of the capivasertib group (two-sided p=0·0044; one-sided log rank test p=0·0018). The most common grade 3–4 adverse events were hypertension (22 [32%] of 69 patients in the capivasertib group *vs* 17 [24%] of 71 in the placebo group), diarrhoea (ten [14%] *vs* three [4%]), rash (14 [20%] *vs* 0), infection (four [6%] *vs* two [3%]), and fatigue (one [1%] *vs* three [4%]). Serious adverse reactions occurred only in the capivasertib group, and were acute kidney injury (two), diarrhoea (three), rash (two), hyperglycaemia (one), loss of consciousness (one), sepsis (one), and vomiting (one). One death, due to atypical pulmonary infection, was assessed as possibly related to capivasertib treatment. One further death in the capivasertib group had an unknown cause; all remaining deaths in both groups (19 in the capivasertib group and 31 in the placebo group) were disease related.

**Interpretation:**

Progression-free survival was significantly longer in participants who received capivasertib than in those who received placebo. The combination of capivasertib and fulvestrant warrants further investigation in phase 3 trials.

**Funding:**

AstraZeneca and Cancer Research UK.

## Introduction

Breast cancer is the most common cancer diagnosis worldwide, and the oestrogen receptor is expressed in most tumours. Endocrine therapies targeting the oestrogen receptor are an integral component of treatment for oestrogen receptor-positive breast cancer, but resistance develops in almost all patients with advanced disease. Several resistance mechanisms have been identified, including alteration of the PI3K/AKT pathway. This pathway is altered in more than 50% of oestrogen receptor-positive advanced breast cancers, most frequently through somatic hotspot mutation in exons 9 and 20 of *PIK3CA*, encoding the p110α isoform of PI3K.^1^, ^2^, ^3^, ^4^ Less frequently, pathway alteration is induced by loss of function mutation or deletion of the negative regulator *PTEN* or activating mutations in *AKT1.* PI3K pathway alteration is associated with endocrine therapy resistance through ligand independent activation of the oestrogen receptor.^5^, ^6^ Conversely, preclinical data show compensatory increases in ligand-dependent oestrogen receptor transcription following PI3K pathway inhibition.^7^, ^8^, ^9^ A rationale therefore exists for simultaneously targeting both the oestrogen receptor and PI3K pathways.

**Research in context**

**Evidence before this study**

We searched PubMed between Jan 1, 2009, and July 31, 2019, to identify publications directly relevant to the FAKTION clinical setting using the search terms “AKT” or “PI3K” or “mTOR” and “oestrogen receptor” and “breast cancer” and “metastatic” and “inhibitor” or “inhibition”. We also searched PubMed for publications in the same period using the terms “capivasertib” or “AZD5363”. We did not use any language restrictions in our search. We found no reports of randomised trials investigating the inhibition of AKT in combination with endocrine therapies in oestrogen receptor-positive, HER2-negative breast cancer. The only other randomised phase 2 study of AKT inhibition in patients with oestrogen receptor-positive, HER2-negative breast cancer showed no advantage of addition of capivasertib to paclitaxel chemotherapy. Five randomised, placebo-controlled trials tested the addition of PI3K or mTOR inhibitors to endocrine therapies. These studies have shown pan-PI3K and beta-sparing inhibitors to have an unfavourable toxicity profile and low clinical activity, and they are no longer in development for this indication. The alpha-specific PI3K inhibitor, alpelisib has activity in combination with fulvestrant, but only in *PIK3CA*-mutant tumours and toxicity remains problematic. mTOR inhibition with everolimus has shown activity, again at the cost of substantial toxicity, but the effect is agnostic to perturbation of the PI3K pathway.

**Added value of this study**

To our knowledge, this study is the first randomised trial to report on the addition of an AKT inhibitor to endocrine therapy in oestrogen receptor-positive metastatic breast cancer after previous aromatase inhibitor therapy. The results showed an improvement in progression-free survival and response rate with addition of capivasertib to endocrine therapy, suggesting synergy, in contrast to the poor efficacy in combination with chemotherapy. Adverse events were common, but manageable with dose reduction, and did not seem to compromise efficacy.

**Implications of all the available evidence**

Several approaches to targeting the PI3K/AKT/mTOR pathway have been shown to be effective in metastatic oestrogen receptor-positive, HER2-negative breast cancer in combination with endocrine therapy. AKT and mTOR inhibition seems to be active in a broader population of patients than PI3K inhibitors. The intermittent scheduling of capivasertib in this study contrasts with the continuous treatment with PI3K and mTOR inhibitors in the majority of previous publications, potentially resulting in improved tolerability. The FAKTION data support further investigation of AKT inhibition with capivasertib in combination with fulvestrant in a phase 3 trial.

Several clinical trials have reported improved progression-free survival with inhibitors of the PI3K pathway in combination with endocrine therapies. Distal inhibition with the mTORC1 inhibitor everolimus improved progression-free survival in combination with the aromatase inhibitor exemestane irrespective of the alteration status of the PI3K pathway, albeit at the cost of additional toxicity.^3^, ^10^ By contrast, proximal pathway inhibition with the PI3Kα-subunit specific inhibitor alpelisib significantly enhanced the efficacy of fulvestrant, but only in tumours harbouring *PIK3CA* hotspot mutations.^11^ This finding has led to selected approval for alpelisib, in combination with fulvestrant, in this specific subgroup of patients with oestrogen receptor-positive advanced breast cancer. An unmet need therefore remains for patients whose tumours do not carry *PIK3CA* hotspot mutations.

Capivasertib (AZD5363) is a potent and selective inhibitor of the three AKT isoforms. Preclinical data show synergistic activity with fulvestrant in both endocrine-sensitive and endocrine-resistant models of oestrogen receptor-positive breast cancer.^12^ Preliminary clinical activity was seen with capivasertib monotherapy in heavily pre-treated patients with *AKT1*-mutant solid cancers, including oestrogen receptor-positive breast cancer, but very low single-agent activity in patients with *PIK3CA*-mutant breast and gynaecological cancers.^13^, ^14^ In the phase 2 BEECH trial, capivasertib did not enhance the efficacy of paclitaxel in oestrogen receptor-positive advanced breast cancer, although endocrine therapy was not permitted in this study as is standard practice.^15^ In a phase 1b study, we determined the capivasertib dose to be used in combination with fulvestrant was 400 mg twice daily for 4 days then 3 days off in a weekly schedule.^16^

In the phase 2 FAKTION trial, we assessed the efficacy and safety of capivasertib plus fulvestrant in postmenopausal women with aromatase inhibitor-resistant, oestrogen receptor-positive, HER2-negative advanced or metastatic breast cancer. Furthermore, the relative efficacy of the treatment combination in participants with and without alteration of the PI3K pathway was assessed.

## Methods

### Study design and participants

We did an investigator-initiated, multicentre, randomised, double-blind, placebo-controlled, biomarker-adaptive, phase 2 trial, in which patients were enrolled from 19 hospitals in the UK (appendix p 20). Eligible patients were postmenopausal women aged at least 18 years with locally confirmed oestrogen receptor-positive, HER2-negative metastatic or locally advanced inoperable breast cancer and Eastern Cooperative Oncology Group performance status of 0–2. Oestrogen receptor-positive was defined as at least 10% of tumour cells staining positive for oestrogen receptor in the primary tumour or a metastatic sample. If no percentage score was available, a Quick (Allred) score of at least 4 out of 8 was considered oestrogen receptor-positive. Participants' cancers were required to have relapsed on or within 12 months of adjuvant aromatase inhibitor therapy or have progressed on an aromatase inhibitor in the metastatic setting (although this did not need to be the most recent therapy). Participants were categorised as having either primary or secondary resistance to aromatase inhibitor therapy. Primary resistance was defined as either disease relapse during or within 6 months of completing aromatase inhibitor treatment in the adjuvant setting, or disease progression within 6 months of starting aromatase inhibitor treatment and no response to aromatase inhibitor treatment in the metastatic setting. Secondary resistance was defined as disease relapse more than 6 months after completion of aromatase inhibitor treatment in the adjuvant setting, or disease progression following achievement of clinical benefit with aromatase inhibitor treatment in the metastatic setting. Radiological or objective clinical evidence of relapse or progression on or after the last systemic therapy before enrolment was required, but not further defined in the protocol. Participants could have measurable or non-measurable disease according to Response Evaluation Criteria in Solid Tumours (RECIST) version 1.1. Up to three previous lines of endocrine therapy and one line of cytotoxic chemotherapy were permitted for metastatic breast cancer. Participants were required to have a life expectancy of at least 12 weeks and adequate organ function (absolute neutrophil count ≥1·0 × 10^9^ per L; platelet count ≥100 × 10^9^ per L; haemoglobin ≥9 g/dL; international normalised ratio ≤1·5; potassium, calcium [corrected for serum albumin], and magnesium within normal limits for the institution; serum creatinine ≤1·5 times the upper limit of normal; alanine aminotransferase and aspartate aminotransferase ≤1·5 times the upper limit of normal [or <3·0 times if liver metastases]; total bilirubin ≤1·5 times the upper limit of normal; and fasting glucose <7·0 mmol/L). Key exclusion criteria included previous treatment with fulvestrant or inhibitors of the PI3K/AKT pathway, and clinically significant abnormalities of glucose metabolism. Participants with well controlled type 2 diabetes with an Hba1c of less than 42 mmol/mol (<8%) and fasting blood glucose of less than 9·3 mmol/L could participate following a protocol amendment on June 11, 2015. A further protocol amendment on Feb 27, 2017, was implemented to allow women with previous malignancy, in remission for at least 5 years, and those with bilateral oophorectomy (to define menopause) to participate. Protocol deviations are outlined in the appendix (p 4), as are the full inclusion and exclusion criteria (appendix pp 21–116).

Written, informed consent was obtained from all participants before trial screening procedures and enrolment. The trial was approved by the North West—Haydock Research Ethics Committee, Manchester, UK (reference number 13/NW/0842). The trial was done in accordance with the principles of Good Clinical Practice, the Declaration of Helsinki, and UK clinical trial regulations.

### Randomisation and masking

Participants were randomly assigned (1:1) to receive fulvestrant plus capivasertib or fulvestrant plus placebo. Randomisation was done centrally, using minimisation with a 20% random element.^17^ Minimisation factors were measurable versus non-measurable disease, primary versus secondary resistance to a third-generation aromatase inhibitor, *PIK3CA* mutation status (mutated *vs* wild-type), and PTEN expression status (null *vs* detected in ≥1% of tumour cells at moderate or strong intensity or ≥10% of cells at weak intensity). An interactive web-response system based on blinded drug pack number was used for treatment allocation. Participants were assigned six-digit trial numbers and treatment groups and a confirmatory email including the participant's trial number, initials, date of birth, and treatment kit numbers was sent to the investigator. Capivasertib tablets and matching placebo had identical packaging, labelling, appearance, and administration schedules. Investigators used the interactive web-response system to receive new kit numbers for subsequent cycles. Participants, investigators, study site staff, and sponsor were masked to treatment assignment until database lock.

### Procedures

Participant blood and tissue samples were obtained after consent and centrally tested at the All Wales Medical Genetics Service and the Department of Cellular Pathology, University Hospital of Wales, Cardiff, UK, for *PIK3CA* and PTEN alteration status before randomisation. If there was an incomplete dataset at the time of randomisation, the patient could still be randomly assigned using the unobtainable category.

Fulvestrant 500 mg was administered on day 1 of every cycle as two intramuscular injections, one into each buttock, and an additional loading dose was delivered at cycle 1 day 15. Capivasertib 400 mg or matching placebo was given orally twice daily on an intermittent weekly schedule of 4 days on and 3 days off, starting on cycle 1 day 15 (to facilitate testing of biomarkers before randomisation). Participants continued to receive study treatment until disease progression, development of unacceptable toxicities, loss to follow-up, or withdrawal of consent. Fulvestrant and capivasertib were manufactured and provided by AstraZeneca (Cambridge, UK) and distributed by Fisher Clinical Services (Horsham, UK).

Participants were reviewed in clinic for toxicities and laboratory monitoring on day 1 of every cycle, at the end of treatment, and 30 days after treatment. Participants who discontinued treatment before progression were also monitored monthly. Blood was drawn for analysis of sodium, potassium, urea, creatinine, albumin, alanine transaminase or aspartate transaminase, alkaline phosphatase, bilirubin, calcium and full blood count on day 1 of every cycle (and day 15 of cycle 1) to cycle 7 and every three cycles thereafter. Random blood glucose sampling followed the same pattern, but was replaced with fasting blood glucose on cycle 1 day 15, cycle 2 day 1, and cycle 3 day 1. Participants performed home urine dipstick for glucose on the third day of capivasertib or placebo dosing each week.

Participants completed drug diaries, which were returned to the study site at each study visit to aid data collection.

The incidence and severity of adverse events and serious adverse events were recorded throughout the study period with haematological and biochemical laboratory tests recorded every 4 weeks. Serious adverse events could be reported at any time. Adverse events were classified according to the National Cancer Institute Common Terminology Criteria for Adverse Events version 4.03. Suspected causal associations between study drugs and serious adverse events were based on investigators' judgment. Toxicities suspected to be related to capivasertib were managed by dose interruption or dose reduction to 320 mg, then 240 mg, then 160 mg at the same schedule. Repeated dose interruptions and continuous interruption of up to 28 days were allowed. Participants were to be discontinued following a single dose interruption of more than 28 days. Dose reduction of fulvestrant to 250 mg was allowed after discussion with the chief investigators if an investigator felt that unacceptable toxicity could reasonably be attributed to fulvestrant or if there were physical difficulties with administration of bilateral injections.

CT scans of the chest, abdomen, and, if indicated, pelvis were done within 28 days before registration to confirm eligibility, and repeated every 8 weeks until cycle 7, then every 12 weeks until disease progression. Participants who discontinued study drugs for any reason other than progression continued to undergo imaging assessments until progression. Scans were assessed according to RECIST by local radiologists, without central review, to determine tumour response and date of progression.

Baseline PTEN status was assessed centrally at the University Hospital Wales Cellular Pathology Department (Cardiff, UK). Freshly cut sections (5 μm thick) were taken from each formalin-fixed paraffin-embedded tumour block. The first section was stained with haematoxylin and eosin to identify the area of greatest tumour density and tumour percentage, and the rest was made available for DNA extraction and whole section immunohistochemistry. Immunohistochemistry was prepared using DAKO (Ely, UK) PTEN Monoclonal Mouse Anti-HumanClone (code M3627), with antigen retrieval done with high pH DAKO Target Retrieval Solution and the DAKO Autostainer Link 48 automated strainer with a pre-detection dilution of 1:100. The validation study and full protocol for the PTEN immunohistochemistry detection have been published previously.^18^ The PTEN protein expression was documented using a numeric intensity score of the cytoplasmic staining in comparison with the surrounding stroma and macrophages (0: null; 1: weak; 2: moderate; and 3: strong).

The protocol definition of PTEN loss, for inclusion of a patient in the altered pathway subgroup, was either immunohistochemistry 0 or weak PTEN staining in less than 10% of cancer cells.

Baseline *PIK3CA* mutational status was assessed centrally at the All Wales Genetics Laboratory (Cardiff, UK). Paraffin-embedded tissue samples were macrodissected and the DNA extracted using the Promega (Madison, WI) Maxwell RSC DNA FFPE kit (AS1450). Upon arrival of the blood samples (and within 96 h of collection), each tube was spun at 2000 g for 10 min at 4°C. Plasma and buffy coat were separated from the red blood cells and spun again to ensure no red blood cells remained in the sample. Plasma and buffy coat were stored in 1 mL aliquots at −80°C until DNA extraction. Cell-free DNA was extracted using the Qiagen (Hilden, Germany) QIAamp circulating nucleic acids kit (55114).

The protocol specified that pathway alteration was defined as either a hotspot mutation detected by digital droplet PCR (ddPCR) on *PIK3CA* exons 9 or 20 in tumour tissue or blood or an immunohistochemistry null status for PTEN in tumour tissue (primary tumour or metastatic biopsy). The method of mutational analysis changed from pyrosequencing to ddPCR during the trial (from Nov 9, 2016), which provided greater sensitivity to detect mutations. 14 patients who were categorised as non-altered on the basis of pyrosequencing analysis had insufficient material to carry out a repeat ddPCR analysis. Tissue DNA and cell-free DNA were analysed by ddPCR using the BioRad (Watford, UK) QX 200 system. Samples were analysed for the common *PIK3CA* exon 9 and 20 mutations (reference sequence NM_006218.2).

In cases where the *PIK3CA* alteration status was changed by the sequential analysis of plasma and tissue and the shift to using ddPCR, the participant was analysed according to the final result.

### Outcomes

The primary endpoint was investigator-assessed progression-free survival, defined as the time from randomisation to either the first documented progression confirmed by RECIST criteria (regardless of whether the patient withdrew from study therapy or received another anti-cancer therapy before progression or death from any cause). Secondary endpoints were overall survival (defined as the time from randomisation to death from any cause), objective response (defined as the proportion of participants with a complete or partial response, according to RECIST version 1.1) and clinical benefit (defined as the proportion of participants with an objective response or stable disease lasting ≥24 weeks). Analysis of the effect of PI3K pathway alteration on these outcomes was planned prospectively and subgroup analyses of progression-free survival, overall survival, and objective response by PI3K pathway alteration were additional secondary outcomes. Subgroup analyses of patients with measurable disease was also planned to assess progression-free survival, objective response, and duration of response in this subgroup of patients. Also evaluated were tolerability and feasibility of the regimen, as shown by the number of participants discontinuing or requiring dose modifications; fulvestrant pharmacokinetics, specifically whether capivasertib affected trough fulvestrant concentrations; and safety, defined as the frequency and severity of adverse events reported during follow-up.

A more extensive, preplanned, exploratory biomarker analysis is currently underway, and when completed, the results will be published in a follow-up paper.

### Statistical analysis

The hypothesis was that participants treated with fulvestrant plus capivasertib would have improved median progression-free survival compared with those treated with fulvestrant plus placebo. The sample size was calculated for a phase 2 screening design, based on a primary outcome of progression-free survival, assuming a time-to-event hazard ratio of 0·65, 90% power, a one-sided alpha of 0·20, and an overall loss to follow-up of 10%.^19^ Under the assumption that the estimated progression-free survival in the control group would be 5·4 months, a total of 98 events were required in 138 participants with 18-month accrual and 6-month minimum follow-up. If recruitment was restricted to participants with *PIK3CA* mutations, 70 events in 98 participants would be required to provide 90% power to detect a hazard ratio of 0·6 in favour of the combination of fulvestrant with capisavertib.

An interim analysis of change in tumour size 8 weeks after randomisation in the first 40 participants without pathway alteration was planned to allow adaptation of recruitment according to participants' pathway alteration status. This approach was designed to look for an early futility signal in the non-altered participant group and to determine whether the trial should continue only in patients with tumours harbouring an altered pathway. Details of this analysis are outlined in the study protocol (appendix). This analysis showed that activity in the non-altered group exceeded the prespecified threshold and the independent data monitoring committee determined that recruitment should remain open to all patients regardless of pathway alteration tumour status.

Efficacy analyses for progression-free survival were done in the full analysis set, comprising all randomly assigned patients, on an intention-to-treat basis. Safety analyses included all participants who had received at least one dose of study drug. Time to event distributions were estimated with the Kaplan-Meier method. Participants with no follow-up RECIST assessment were censored at day 1. Participants without disease progression confirmed by RECIST and those who died or progressed after missing the last two RECIST assessments were censored for progression-free survival at the date of the last evaluable RECIST assessment or at the point of withdrawal of consent. Progression-free survival was compared with a one-sided unadjusted log-rank test (the primary analysis). Cox regression was used to estimate hazard ratios with confidence intervals and p values; multivariable Cox regression was used to adjust the estimates for the randomisation minimisation variables. The proportional hazards assumption was checked using Cox-Snell residuals and Schoenfeld's global test. Overall survival was summarised and analysed in the same way as progression-free survival; participants still alive were censored at the date last seen. The proportion of patients with objective response and clinical benefit was summarised by trial group and analysed using logistic regression.

Two post-hoc analyses were done to calculate the progression-free survival of participants allocated to capivasertib who discontinued or reported a dose reduction and the duration of response (defined as the time from first documented objective response to the first documented progression or death) for participants with measurable disease in both the capivasertib and placebo groups. A per-protocol sensitivity analysis was also done (appendix p 3).

Analyses were done using Stata (version 14.0). Apart from the primary outcome, which had a significance threshold of 0·2, all p values were considered significant at the 0·05 threshold. The independent data monitoring committee reviewed accumulating accrual, safety and treatment data at regular intervals; however, there were no formal stopping guidelines.

This trial is registered with ClinicalTrials.gov, number NCT01992952.

### Role of the funding source

The funder (AstraZeneca) supplied capivasertib, matching placebo, and fulvestrant, contributed to the study design, reviewed the draft analysis plan, and provided critical review of the draft report, including interpretation, but had no role in data collection or data analysis. The co-funder (Cancer Research UK) approved the study design, but had no role in the drafting of the report, or data collection, analysis, or interpretation. The sponsor of the study had no role in the writing of the report. The corresponding author had full access to all the data in the trial and had final responsibility for the decision to submit for publication. Additionally, AC and CC had full access to the raw data.

## Results

**Figure 1:**
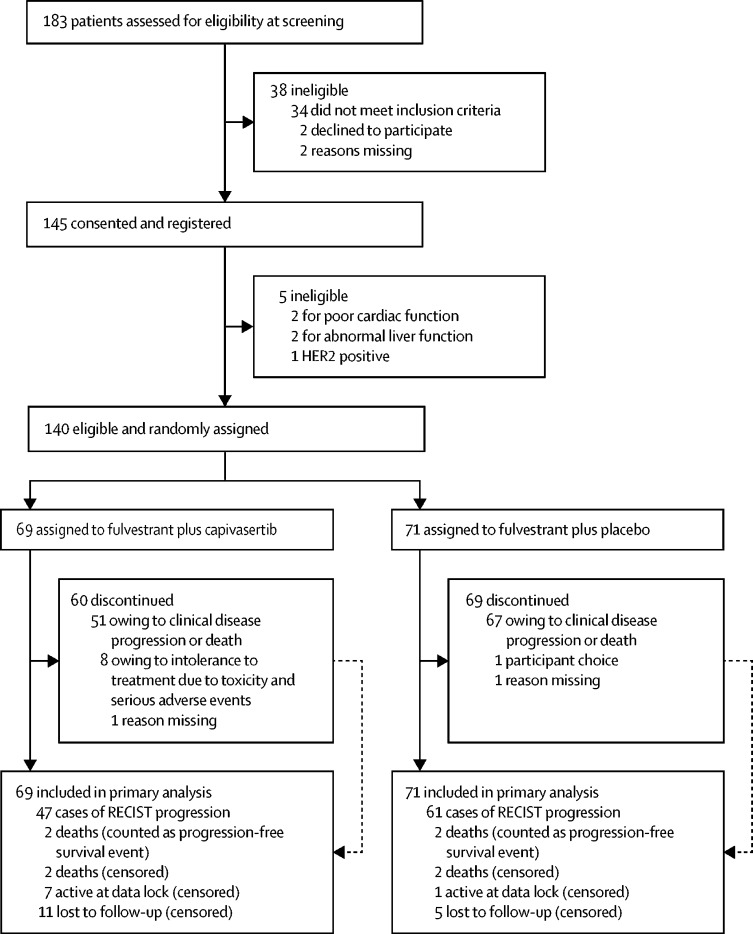
Trial profile

Between March 16, 2015, and March 6, 2018, 183 patients were screened for eligibility and 140 were randomly assigned to receive fulvestrant plus capivasertib (n=69 [49%]) or fulvestrant plus placebo (n=71 [51%]; figure 1). All randomly assigned participants were included in primary efficacy and safety analyses. Participants were followed up until all had had at least 6 months follow-up and the minimum 98 disease progression events required for analysis were confirmed. Median progression-free survival follow-up was 4·9 months (IQR 1·6–11·6). Treatment groups were well balanced for baseline characteristics (table 1).

**Table 1:** Baseline characteristics

		**Fulvestrant plus capivasertib group (n=69)**	**Fulvestrant plus placebo group (n=71)**
Median age, years (IQR); range		62 (55–68); 42–81	61 (53–68); 40–82
Eastern Cooperative Oncology Group performance status (physical examination)			
	0	42 (61%)	49 (69%)
	1	25 (36%)	17 (24%)
	2	1 (1%)	2 (3%)
	Missing	1 (1%)	3 (4%)
Histopathological subtype			
	Invasive ductal carcinoma	57 (83%)	58 (82%)
	Invasive lobular cancer	4 (6%)	12 (17%)
	Mixed invasive ductal carcinoma and invasive lobular cancer	5 (7%)	0
	Other	3 (4%)	1 (1%)
Stage			
	III inoperable	0	1 (1%)
	IV	68 (99%)	68 (96%)
	Missing	1 (1%)	2 (3%)
Number of disease sites			
	Median (IQR); range	2 (2–3); 1–5	2 (1–3); 1–5
	1	15 (22%)	19 (27%)
	≥2	54 (78%)	52 (73%)
Metastatic sites*			
	Brain	1 (1%)	1 (1%)
	Liver	32 (46%)	29 (41%)
	Lung	30 (43%)	28 (39%)
	Bone	58 (84%)	55 (77%)
	Lymph	28 (41%)	30 (42%)
	Pericardial or pleural	5 (7%)	3 (4%)
	Chest wall or skin	1 (1%)	3 (4%)
	Other visceral	2 (3%)	1 (1%)
Visceral disease		49 (71%)	47 (66%)
Measurable disease†		49 (71%)	50 (70%)
Primary or secondary aromatase inhibitor resistance†			
	Primary	25 (36%)	26 (37%)
	Secondary	44 (64%)	45 (63%)
Aromatase inhibitor given as last treatment before registration		57 (83%)	52 (73%)
Bone-only disease		10 (14%)	8 (11%)
Previous breast surgery		59 (86%)	62 (87%)
Previous adjuvant endocrine therapy		60 (87%)	65 (92%)
	Any tamoxifen	41 (68%)	43 (66%)
	Any aromatase inhibitor	40 (67%)	36 (55%)
	Any gonadotropin-releasing hormone	2 (3%)	1 (2%)
	Other	1 (2%)	1 (2%)
	Missing	0	1 (2%)
Previous adjuvant chemotherapy		36 (52%)	42 (59%)
	Anthracycline based	11 (31%)	13 (31%)
	Taxane based	5 (14%)	5 (12%)
	Anthracycline plus taxane	11 (31%)	9 (21%)
	Cyclophosphamide, methotrexate, and fluorouracil or capecitabine	7 (19%)	14 (33%)
	Other	1 (3%)	1 (2%)
	Missing	1 (3%)	0
Previous endocrine treatment (metastatic or locally advanced setting)			
	Median lines (IQR); range	1 (1–2); 0–3	1 (1–2); 0–3
	0 lines	9 (13%)	6 (8%)
	1 line	39 (57%)	45 (63%)
	≥2 lines	20 (29%)	20 (28%)
	Missing	1 (1%)	0
Metastatic chemotherapy for advanced breast cancer		17 (25%)	20 (28%)
	Capecitabine based	3 (18%)	6 (30%)
	Taxane based	8 (47%)	8 (40%)
	Anthracycline based	2 (12%)	6 (30%)
	Combined anthracycline and taxane	3 (18%)	0
	Other	1 (6%)	0
Pathway altered (subgroup A)		31 (45%)	28 (39%)
Pathway non-altered (subgroup B)		38 (55%	43 (61%)
*PIK3CA* results—blood			
	Wild-type	54 (78%)	57 (80%)
	Mutation	14 (20%)	13 (18%)
	Missing	1 (1%)	1 (1%)
*PIK3CA* results—tissue			
	Wild-type	34 (49%)	43 (61%)
	Mutation	22 (32%)	19 (27%)
	Missing	13 (19%)	9 (13%)
*PIK3CA* results—blood or tissue†			
	Wild-type	42 (61%)	47 (66%)
	Mutation	27 (39%)	24 (34%)
	Missing	0	0
PTEN results†			
	0	4 (6%)	4 (6%)
	1	9 (13%)	8 (11%)
	2	13 (19%)	23 (32%)
	3	34 (49%)	28 (39%)
	Missing	9 (13%)	8 (11%)

Data are n (%), unless otherwise specified. The displayed percentages do include missing values.

* Sites are not mutually exclusive.

† Randomisation minimisation factor.

**Figure 2:**
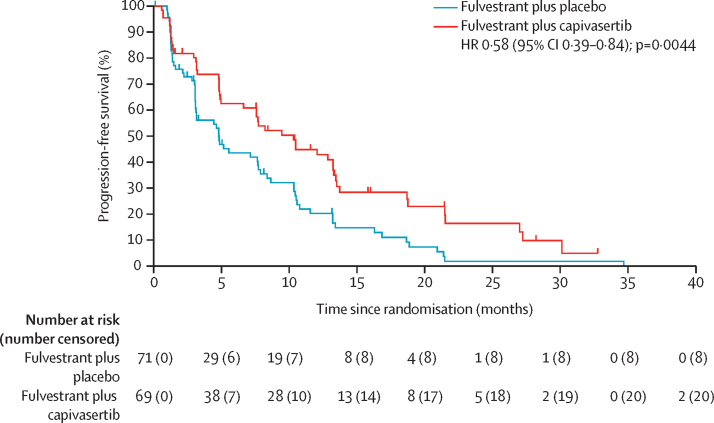
Progression-free survival HR=hazard ratio.

At the time of primary analysis, there had been 112 progression-free survival events: 49 (71%) in 69 patients in the capivasertib group compared with 63 (89%) in 71 in the placebo group. The Schoenfeld's tests were consistent with the proportional hazards assumption, and the assumption was thus adequately met (appendix p 2). Median progression-free survival was 10·3 months (95% CI 5·0–13·2) in the capivasertib group versus 4·8 months (3·1–7·7) in the placebo group, giving an unadjusted hazard ratio (HR) of 0·58 (95% CI 0·39–0·84, 2-sided p=0·0044) and an adjusted HR of 0·58 (0·39–0·85, 2-sided p=0·0049; figure 2) in favour of the capivasertib plus fulvestrant group. The unadjusted log-rank test gave a one-sided p value of 0·0018. The trial, therefore, met its primary endpoint.

99 (71%) participants had measurable disease (49 in the capivasertib group and 50 in the placebo group). Median progression-free survival with the combination of fulvestrant and capivasertib was significantly longer than in the placebo group, both in patients with measurable disease (7·6 months [95% CI 4·8–10·5] *vs* 3·2 months [3·1–7·7]) with a HR of 0·61 (95% CI 0·39–0·95, p=0·030) and those with non-measurable disease (13·4 months [95% CI 7·7–30·1] *vs* 7·9 months [4·4–10·8]) with a HR of 0·47 (95% CI 0·22–0·99, 2-sided p=0·046).

20 (29%) of 69 in the capivasertib group achieved an objective response compared with six (8%) of 71 in the placebo group (odds ratio [OR] 4·42, 95% CI 1·65–11·84, 2-sided p=0·0031). 38 (55%) of 69 in the capivasertib group had clinical benefit versus 29 (41%) of 71 in the placebo group (OR 1·78, 95% CI 0·91–3·47, 2-sided p=0·093).

20 (41%) of 49 participants with measurable disease in the capivasertib group had an objective response compared with six (12%) of 50 in the placebo group (OR 5·06, 95% CI 1·81–14·11, 2-sided p=0·0020). In a post-hoc analysis, the median duration of response in patients with measurable disease was 7·1 months (95% CI 3·8–9·9) for participants in the capivasertib group and 5·0 months (2·7 to not reached) for participants in the placebo group (appendix p 5).

Assessing the relative efficacy of capivasertib plus fulvestrant in participants with PI3K/PTEN pathway altered tumours versus those with non-altered tumours was a prespecified objective of the trial. A full breakdown of *PIK3CA* and PTEN alteration status is given in the appendix (p 2). There were no cases of weak PTEN staining, and all tumours that scored as 0 for immunohistochemistry were completely negative for PTEN staining. These cases are thus termed PTEN null. 59 (42%) of 140 tumours were designated altered (31 [45%] of 69 in the capivasertib group and 28 [39%] of 71 in the placebo group) and 81 (58%) non-altered (38 [55%] of 69 in the capivasertib group and 43 (61%) of 71 in the placebo group). The combined result of sequential analysis of plasma and tissue and the change to ddPCR led to a conversion from non-altered to altered status in 21 participants; 12 (17%) of 69 patients in the capivasertib group and nine (13%) of 71 patients in the placebo group were converted from non-altered to altered, and three (4%) in the capivasertib group and four (6%) in the placebo group were converted from altered to non-altered.

**Figure 3:**
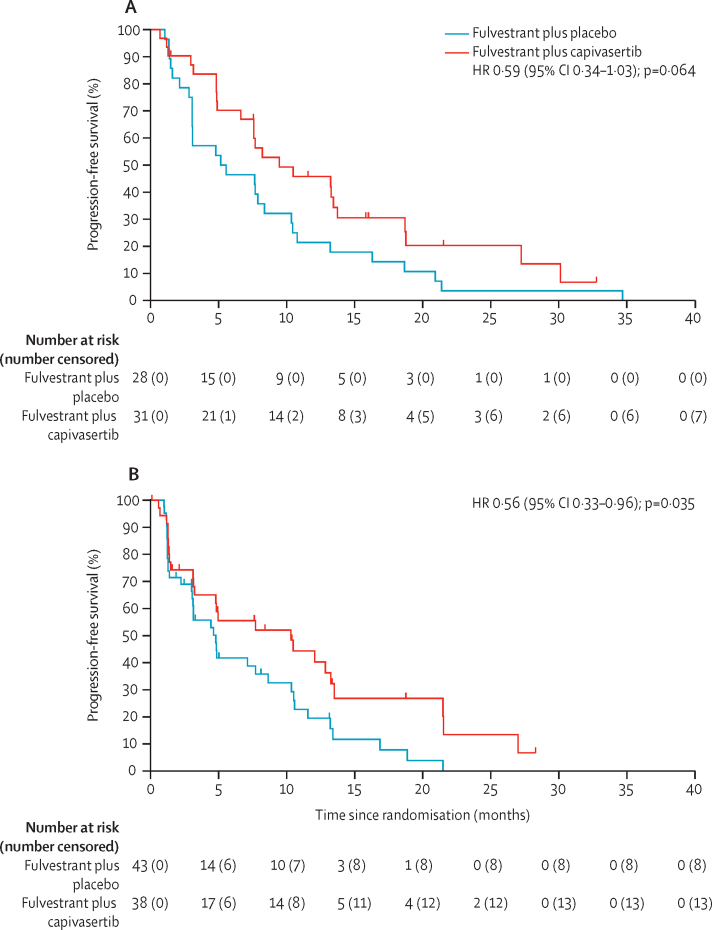
Progression-free survival in subgroups by PI3K pathway alteration status (A) The pathway-altered subgroup. (B) The pathway non-altered subgroup. HR=hazard ratio.

In the PI3K/PTEN pathway non-altered group, median progression-free survival was 10·3 months (95% CI 3·2–13·2) in the capivasertib group and 4·8 months (3·0–8·6) in the placebo group. In the altered group, median progression-free survival was 9·5 months (95% CI 6·6–13·7) in the capivasertib group and 5·2 months (3·1–8·4) in the placebo group (figure 3). The significant improvement in progression-free survival seen with fulvestrant and capivasertib versus placebo in the overall population was preserved in the non-altered group (0·56, 0·33–0·96, p=0·035), but not in patients with PI3K/PTEN pathway altered tumours (0·59, 0·34–1·03, p=0·064). In participants with measurable disease, nine (47%) of 19 participants with pathway alteration in the capivasertib group had an objective response compared with two (11%) of 19 in the placebo group (OR 7·65, 95% CI 1·37–42·71, 2-sided p=0·020). In patients without pathway alteration, 11 (37%) of 30 in the capivasertib group had an objective response compared with four (13%) of 31 in the placebo group (OR 3·91, 95% CI 1·08–14·14, 2-sided p=0·038).

**Figure 4:**
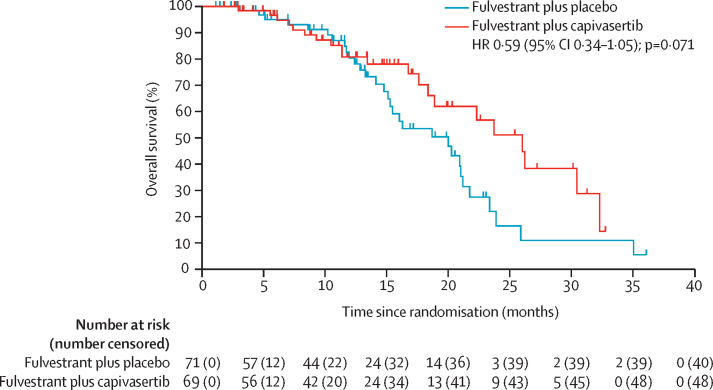
Overall survival Censored patients are marked on the curves with a vertical dash. One patient in the placebo group was censored at 36 months. HR=hazard ratio.

Overall survival data are immature with a median follow-up for survival of 12 months (IQR 6–17). 21 (30%) of 69 patients in the capivasertib group and 31 (44%) of 71 in the placebo group had died at data cutoff. The median overall survival was 26·0 months (95% CI 18·4–32·3) in the capivasertib group and 20·0 months (15·1–21·2) in the placebo group with a HR of 0·59 (95% CI 0·34–1·05, 2-sided p=0·071; figure 4). In the non-altered group, median overall survival was 23·7 months (95% CI 16·8 to not reached) in the capivasertib group and 20·3 months (13·3–23·4) in the placebo group (HR 0·62, 95% CI 0·30–1·28, 2-sided p=0·20). In the altered group, median overall survival was 30·5 months (95% CI 17·6 to not reached) in the capivasertib group and 18·7 months (95% CI 14·1–21·2) in the placebo group (HR 0·53, 95% CI 0·21–1·33, 2-sided p=0·17).

The median duration of fulvestrant treatment was 9·2 months (IQR 3·0–14·1) in the capivasertib group compared with 4·6 months (2·8–10·5) in the placebo group. The median duration of capivasertib treatment itself was slightly shorter than that of fulvestrant in the combined group at 7·7 months (IQR 1·5–13·5) due to discontinuation of capivasertib before disease progression in eight (12%) patients. The median duration of placebo administration was 4·9 months (IQR 2·3–10·6).

Overall, 28 (41%) of 69 patients in the capivasertib group had a capivasertib dose reduction compared with one (1%) of 71 in the placebo group; more specifically, 22 had one, five had two, and one had three capivasertib dose reductions. Eight (12%) participants discontinued capivasertib because of adverse events—five without and three with a previous dose reduction. Of these eight participants, rash was reported in six, diarrhoea in three, and hypoglycaemia, nausea, vomiting, mouth ulcers, and sweating in one each. The most common toxicities leading to a dose reduction were rash (14 [20%]), diarrhoea (eight [12%]), and nausea or vomiting (three [4%]). In a post-hoc exploratory analysis, the median progression-free survival in 33 participants requiring a dose reduction or discontinuation of capivasertib for toxicities was 13·5 months (95% CI 7·7–21·5), suggesting that dose reduction did not compromise treatment efficacy.

The proportion of participants who had grade 3–5 adverse events (irrespective of causality) was 45 (65%) of 69 in the capivasertib group and 35 (50%) of 70 in the placebo group (table 2; appendix pp 6–18). One patient in the placebo group had a grade 5 haemorrhage, attributed to disease progression. All cases of severe diarrhoea, rash, hyperglycaemia, and vomiting were grade 3, apart from one grade 4 diarrhoea in the placebo group. The most common grade 3–4 adverse events were hypertension (22 [32%] of 69 in the capivasertib group *vs* 17 [24%] of 71 in the placebo group), diarrhoea (ten [14%] *vs* three [4%]), rash (14 [20%] *vs* 0), infection (four [6%] *vs* two [3%]), and fatigue (one [1%] *vs* three [4%]). Serious adverse reactions (reported only in the capivasertib group) were acute kidney injury (two), diarrhoea (three), hyperglycaemia (one), loss of consciousness (one), rash (two), sepsis (one), and vomiting (one; appendix p 7). One death on treatment with capivasertib, from an atypical pulmonary infection without disease progression, was considered possibly treatment related. One death in the capivasertib treatment group had an unknown cause, and all remaining deaths in both groups (19 in the capivasertib group and 31 in the placebo group) were disease related.

**Table 2:** Adverse events

	**Fulvestrant plus capivasertib group (n=69)**				**Fulvestrant plus placebo group (n=71)**			
	Grade 1	Grade 2	Grade 3	Grade 4	Grade 1	Grade 2	Grade 3	Grade 4
Diarrhoea	28 (41%)	18 (26%)	10 (14%)	0	21 (30%)	1 (1%)	2 (3%)	1 (1%)
Rash*	15 (22%)	7 (10%)	14 (20%)	0	11 (15%)	2 (3%)	0	0
Hyperglycaemia	17 (25%)	9 (13%)	3 (4%)	0	10 (14%)	0	0	0
Proteinuria	18 (26%)	8 (12%)	0	0	8 (11%)	0	0	0
Hypertriglyceridaemia	32 (46%)	4 (6%)	1 (1%)	0	18 (25%)	2 (3%)	0	0
Infection (including urinary tract infection)	8 (12%)	14 (20%)	3 (4%)	1 (1%)	5 (7%)	6 (8%)	2 (3%)	0
Vomiting	17 (25%)	8 (12%)	2 (3%)	0	13 (18%)	2 (3%)	0	0
Urea (high)	12 (17%)	1 (1%)	0	0	6 (8%)	0	0	0
Creatinine increased	9 (13%)	2 (3%)	0	0	4 (6%)	0	0	0
Back pain	12 (17%)	5 (7%)	0	0	7 (10%)	3 (4%)	1 (1%)	0
Urinary tract infection	4 (6%)	6 (9%)	1 (1%)	0	2 (3%)	3 (4%)	0	0
ECG QTc prolonged	22 (32%)	2 (3%)	0	0	15 (21%)	4 (6%)	0	0
Cholesterol high	19 (28%)	4 (6%)	0	0	16 (23%)	2 (3%)	0	0
Haemoglobin (low)	14 (20%)	4 (6%)	0	0	7 (10%)	5 (7%)	1 (1%)	0
Hypercalcaemia	16 (23%)	1 (1%)	0	0	12 (17%)	0	0	0
Hypoalbuminaemia	7 (10%)	4 (6%)	0	0	5 (7%)	1 (1%)	0	0
Mucositis oral	7 (10%)	3 (4%)	0	0	5 (7%)	0	0	0
LDL (high)	14 (20%)	0	0	0	10 (14%)	0	0	0
Elevated alanine transaminase	16 (23%)	2 (3%)	0	0	12 (17%)	1 (1%)	2 (3%)	0
Abdominal pain	8 (12%)	2 (3%)	0	0	5 (7%)	1 (1%)	1 (1%)	0
Hypocalcemia	1 (1%)	1 (1%)	0	1 (1%)	0	0	0	0
Nausea	30 (43%)	8 (12%)	0	0	31 (44%)	5 (7%)	0	0
HDL (low)	14 (20%)	0	0	0	12 (17%)	0	0	0
Hyponatraemia	1 (1%)	0	2 (3%)	0	0	0	1 (1%)	0
Hypertension	20 (29%)	21 (30%)	22 (32%)	0	23 (32%)	22 (31%)	17 (24%)	0
Pain (other)	5 (7%)	1 (1%)	2 (3%)	0	5 (7%)	2 (3%)	0	0
Anaemia	2 (3%)	1 (1%)	0	0	0	0	2 (3%)	0
Haemorrhage†	0	0	0	0	0	0	0	0
Cough	5 (7%)	2 (3%)	0	0	5 (7%)	2 (3%)	0	0
Fatigue	24 (35%)	15 (22%)	1 (1%)	0	26 (37%)	12 (17%)	3 (4%)	0
Elevated alkaline phosphatase	15 (22%)	5 (7%)	1 (1%)	0	16 (23%)	4 (6%)	2 (3%)	0
White blood cell count (high)	7 (10%)	3 (4%)	0	0	7 (10%)	4 (6%)	0	0
Pain in extremity	6 (9%)	3 (4%)	0	0	6 (8%)	2 (3%)	2 (3%)	0
Influenza-like symptoms	6 (9%)	2 (3%)	0	0	7 (10%)	2 (3%)	0	0
Neutrophil count decreased	7 (10%)	1 (1%)	0	0	8 (11%)	1 (1%)	0	0
Platelet count decreased	2 (3%)	0	0	1 (1%)	4 (6%)	0	0	0
Hot flashes	8 (12%)	2 (3%)	0	0	12 (17%)	0	0	0
Constipation	6 (9%)	0	0	0	9 (13%)	0	0	0
Injection site reactions	16 (23%)	2 (3%)	1 (1%)	0	21 (30%)	2 (3%)	0	0
Arthralgia	12 (17%)	5 (7%)	2 (3%)	0	17 (24%)	6 (8%)	0	0
Elevated aspartate transaminase	7 (10%)	2 (3%)	0	0	9 (13%)	2 (3%)	2 (3%)	0
Pulse (high)	5 (7%)	0	0	0	9 (13%)	0	0	0
Headache	16 (23%)	1 (1%)	0	0	19 (27%)	4 (6%)	0	0
Dyspnoea	1 (1%)	4 (6%)	0	0	9 (13%)	3 (4%)	1 (1%)	1 (1%)

The table presents toxicities reported in at least 10% of patients in either group or any toxicity reported at grade 3 or worse, irrespective of cause. This table includes laboratory value changes that were confirmed as toxicities by research centres. ECG=electrocardiogram.

* All preferred terms of rash have been combined.

† One patient in the treatment group had a grade 5 haemorrhage.

In the pharmacokinetic analysis, there was no apparent difference in trough fulvestrant concentrations between participants assigned to capivasertib and those receiving placebo (appendix p 19).

## Discussion

The results of this study show that the addition of capivasertib to fulvestrant therapy significantly improved progression-free survival in participants with oestrogen receptor-positive HER2-negative breast cancer that had progressed on an aromatase inhibitor. The trial met its primary objective and, to our knowledge, is the first study to show the efficacy of an AKT inhibitor combined with fulvestrant in this setting. In the subgroup of participants with measurable disease, the addition of capivasertib to fulvestrant significantly improved the objective response compared with placebo plus fulvestrant. However, PI3K pathway alteration status, predefined in the protocol as *PIK3CA* hotspot mutation in exons 9 or 20 or PTEN null by immunohistochemistry, did not seem to change the effect size of capivasertib, although there was not enough statistical power within the subgroups to confirm this finding. The overall survival data from FAKTION are not yet mature, and no statistically significant difference exsists between treatment groups. Extended follow-up and a larger phase 3 study will be required to determine whether the trend for improved survival with capivasertib in FAKTION is a genuine finding.

The role of the PI3K/AKT/PTEN pathway in driving resistance to endocrine therapy is well documented in pre-clinical models.^5^, ^6^ Several clinical trials have tested the addition of targeted therapies designed to inhibit different proteins in this pathway. The mTORC1 inhibitor everolimus significantly improved progression-free survival in combination with exemestane,^10^ which was independent of *PIK3CA* mutation and PI3K pathway alteration status.^3^ However, this improvement was at the expense of an increase in toxicity and overall survival was not significantly improved. Two trials have shown significant improvements in progression-free survival with everolimus versus placebo in combination with fulvestrant, although the relative benefits by PI3K pathway status are yet to be reported.^20^, ^21^ The activity of the α-specific PI3K inhibitor, alpelisib, in combination with fulvestrant was confined to patients whose tumours harboured *PIK3CA* mutations.^11^ Therefore, the role of pathway alteration in determining drug efficacy could be dependent on the specific target and its position in the pathway.

The FAKTION data also contrast with the reported activity of capivasertib or ipatasertib (another pan-AKT inhibitor) in combination with paclitaxel in the PAKT^22^ and LOTUS^23^ studies in metastatic triple-negative breast cancer. Both studies reported a significant improvement in progression-free survival in the overall population, but the benefit was predominantly recorded in tumours with activating mutations in *PIK3CA* or *AKT1* or inactivating alterations in *PTEN*.^22^, ^23^ However, deficient PTEN expression is a more frequent alteration in triple-negative breast cancer than in oestrogen receptor-positive, HER2-negative breast cancer and is associated with increased AKT pathway activation,^24^, ^25^ suggesting that the relative benefit of AKT inhibition is context dependent. The cross-talk between the oestrogen receptor and AKT signalling pathways, which seems to be irrespective of mutational status, provides a rational basis for effectiveness of the investigational combination beyond a mutant population.

The pattern of adverse events observed with capivasertib was consistent with other PI3K/AKT pathway inhibitors, with diarrhoea, rash, and hyperglycaemia being the most prevalent. More than a third (41%) of participants required at least one dose reduction, but only three patients subsequently stopped capivasertib because of toxicity. An additional five participants (8%) stopped capivasertib without previous dose reduction. Although a post-hoc exploratory analysis, the median progression-free survival in those participants who reduced or discontinued capivasertib due to toxicity was not compromised. As such, toxicity management guidelines and patient education are crucial for future studies in order to manage toxicity proactively. Quality of life was not assessed in this phase 2 study, but will be key in future studies to assess the effect of such management strategies.

The treatment environment is changing for patients with oestrogen receptor-positive, HER2-negative metastatic breast cancer. CDK4/6 inhibitors are now routinely used in combination with aromatase inhibitors as first-line therapy with improvements in overall survival.^26^, ^27^ The value of continued CDK4/6 inhibition after progression is being tested in several clinical trials, but new, improved options are clearly needed in this setting. Notably, reduction or loss of PTEN has been implicated in resistance to both endocrine^28^ and CDK4/6 inhibitor therapy,^29^ with such cancers responding to AKT inhibition but not alpha-specific PI3K inhibition. The pre-planned subgroup analysis of the FAKTION study showed that the efficacy of AKT inhibition is likely to be independent of PI3K pathway alteration. If this independence is confirmed, more patients would be eligible for capivasertib than alpelisib therapy, which is approved only in patients with tumours harbouring *PIK3CA* mutations. Elucidation of biomarkers of activity of AKT inhibition in this population remains a research priority; however, based on the results of the FAKTION trial, capivasertib is an attractive candidate for further development in this disease setting.

Analysis of efficacy by pathway alteration was limited to hotspot mutation detection in exon 9 and 20 of *PIK3CA* as well as PTEN status by immunohistochemistry. When the study was designed, *AKT1* mutation was reported in only 1–4% of oestrogen receptor-positive breast cancers;^1^, ^2^ thus *AKT1* testing was not part of the original protocol. *AKT1* mutations have been identified in 6–7% of oestrogen receptor-positive breast cancer metastases,^3^ and a more comprehensive biomarker analysis, including mutational status of all AKT isoforms, is underway on FAKTION samples. The full dataset will inform future trial design and will help to identify the patient population with the greatest potential of benefit from capivasertib treatment.

A strength of the study was the randomised nature of this phase 2 trial, which means that, although of modest size, it provides a rationale for a subsequent phase 3 trial. However, the study also has some limitations. First, it was a phase 2 screening study, with a relaxed type 1 error and one-sided design owing to the interest in detecting an active drug. The design does increase the risk of obtaining a false-positive result; however, we were willing to accept this given that a phase 3 confirmatory trial was planned if there was a positive result. Second, no adjustment of type 1 error was made for multiple testing of secondary and exploratory outcomes, and such adjustments should be considered in a confirmatory study. Third, the modest size of the study did not allow meaningful subgroup analyses, such as for bone-only or visceral disease, but these should be evaluated in subsequent studies. Fourth, premenopausal women and men with advanced breast cancer were excluded. Fifth, no FAKTION participants had received previous CDK4/6 inhibitor therapy, which is now standard of care in combination with an aromatase inhibitor or fulvestrant. However, clinical^30^, ^31^, ^32^ and preclinical data^33^, ^34^ suggest that previous exposure to CDK4/6 inhibition should not abrogate the incremental benefit seen with capivasertib. Preclinical data suggest that AKT inhibition might be superior to PI3K inhibition in this setting,^29^ but formal confirmation of this theory in a randomised trial is required.

In conclusion, FAKTION met its primary outcome in showing that the combination of fulvestrant and capivasertib is tolerable and produces a significant improvement in progression-free survival for participants with advanced oestrogen receptor-positive HER2-negative breast cancer who have previously progressed on an aromatase inhibitor. These data indicate that capivasertib is active in combination with fulvestrant and provide the basis for a confirmatory phase 3 study.

## Data sharing

Any requests for anonymised trial data or supporting material will be reviewed on a case-by-case basis. Only requests that have scientifically and methodologically sound proposals will be considered and the usage of the shared trial data or supporting material will be limited to the approved proposal. The final decision as to whether data or supporting material might be shared and the exact data or supporting material to be shared will be made between the sponsor, trial team, and AstraZeneca. Proposals should be directed to the corresponding author.
