# Supplementary material for: Fulvestrant plus capivasertib versus placebo after relapse or progression on an aromatase inhibitor in metastatic, oestrogen receptor-positive breast cancer (FAKTION): a multicentre, randomised, controlled, phase 2 trial
Source: Lancet Oncol. 2020 Mar;21(3):345–57. doi: 10.1016/S1470-2045(19)30817-4 (PMC7052734; doi:10.1016/S1470-2045(19)30817-4)
Supplement: Supplementary appendix [file mmc1.pdf]

THE LANCET
Oncology

Supplementary appendix

This appendix formed part of the original submission and has been peer reviewed. We post it as supplied by the authors.

Supplement to: Jones RH, Casbard A, Carucci M, et al. Fulvestrant plus capivasertib versus placebo after relapse or progression on an aromatase inhibitor in metastatic, oestrogen receptor-positive breast cancer (FAKTION): a multicentre, randomised, controlled, phase 2 trial. *Lancet Oncol* 2020; published online Feb 5. http://dx.doi.org/10.1016/S1470-2045(19)30817-4.

Figure 1- Waterfall plot of change in target lesions from baseline to the point of best response in patients with measurable disease.

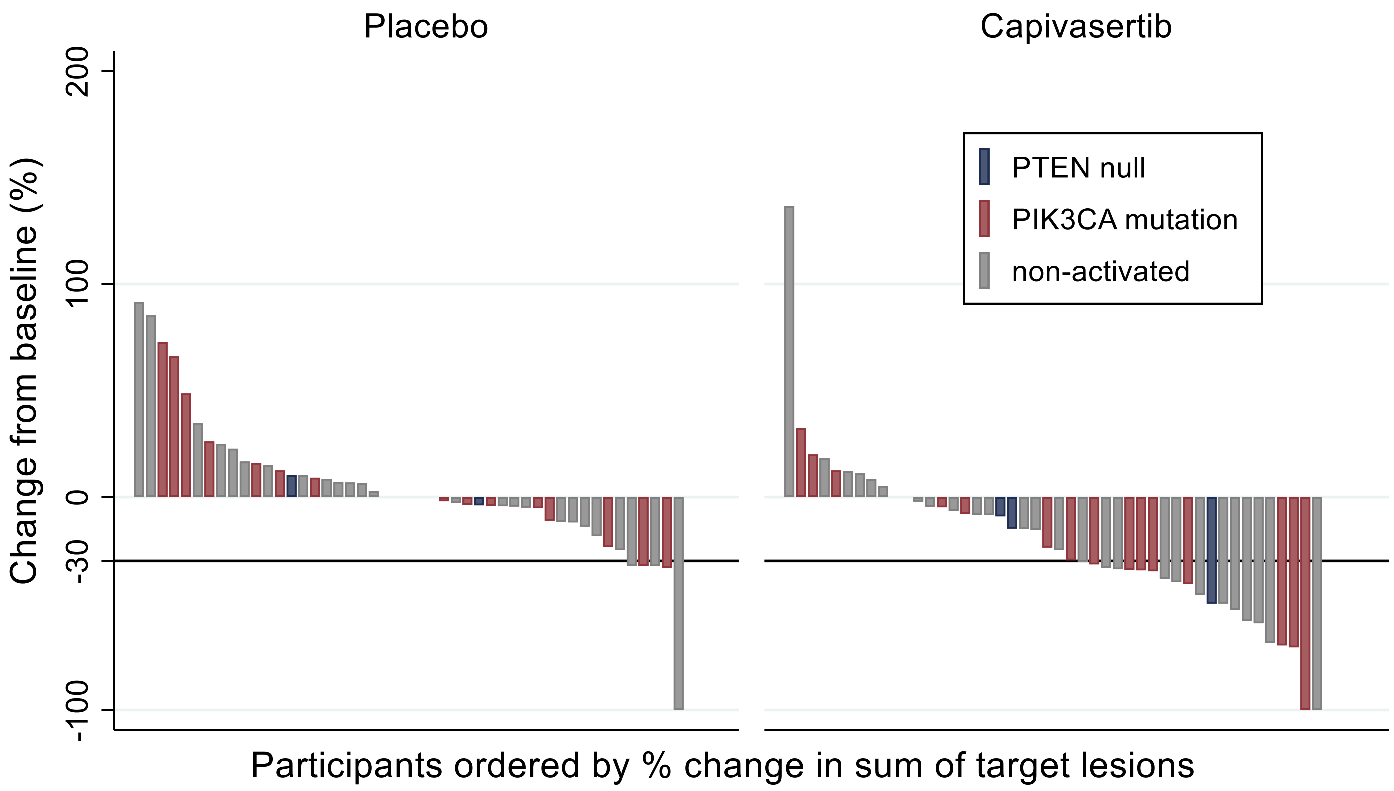

Figure 2 - PIK3CA and PTEN alteration status for each patient by arm, and with source of archival tumour tissue

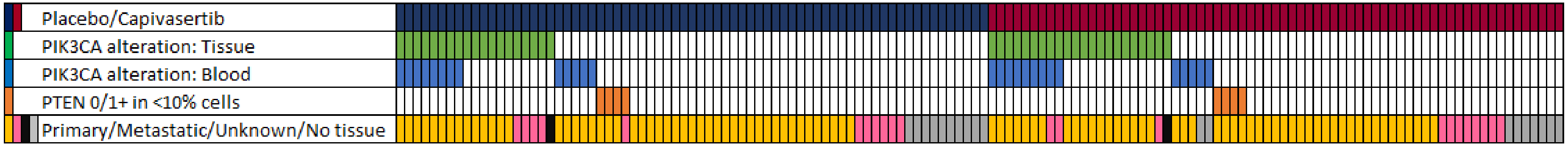

Table 1 – Proportional hazards assumption tests

Comparison for analysis	N	Schoenfeld's test p-value
PFS	140	0.99
PFS PI3K /PTEN pathway non-altered	81	0.29
PFS pathway altered	59	0.52
Overall survival	140	0.64

Per protocol analysis

A sensitivity per protocol analysis (PP1) was performed to ensure that the results were consistent with the primary intention to treat (ITT) analysis after excluding any patients who did not receive treatment; were later found not to meet the eligibility criteria after commencing on the trial; or who were not evaluable for RECIST during follow-up. The per protocol population was identified prior to unblinding.

We found that all patients had received trial treatment, but that sites had accidentally omitted a screening investigation in 28 cases; and randomised the patient after an out of range screening test on 9 occasions. This was despite prior sign-off that all eligibility criteria were met.

These cases were reviewed by the independent data monitoring committee (IDMC) prior to unblinding, and the IDMC recommended that a further per protocol analysis (PP2) should be performed, only excluding four cases of screen fails that could potentially affect patient safety or trial outcomes.

Following unblinding and analysis, the per protocol analyses were performed and the results were consistent with the ITT data (Table 2, below) and therefore do not affect the interpretation of the ITT analysis.

Table 2 – Per protocol analysis

Included participants (PP1)	Capivasertib arm (N=44)		Placebo arm (N=55)	
**Excluded participants**	**16/69 (23%)**		**25/71 (35%)**	
Protocol deviation for missed screening investigation	9		19	
Found to be ineligible post randomisation	6		3	
Participant having non-recommended treatment	0		1	
Not evaluable for RECIST during follow-up	1		2	
RECIST Events	33		48	
Median PFS (months)	12·8	IQR (9·5 - 13·4)	4.6	IQR (3·1 - 7·1)
HR	0·49 (95% CI 0·31 - 0·77); p-value = 0·0021			
Included participants (PP2) - Post IDMC review	Capivasertib arm (N=67)		Placebo arm (N=69)	
**Excluded participants**	**2/69 (3%)**		**2 (3%)**	
Protocol deviation for missed screening investigation	0		0	
Found to be ineligible post randomisation				
fulvestrant given 7 days after radiotherapy	2		0	
fulvestrant given 2 days after anastrazole	0		1	
QTc interval was 474 msec at screening	0		1	
Not evaluable for RECIST during follow-up	0		0	
RECIST Events	47		61	
Median PFS (months)	10·5	IQR (6·6 - 13·3)	4·8	IQR (3·1 -7·7)
HR	0·56 (95% CI 0·38-0·83); p-value = 0·0037			

Table 3 - Objective response and clinical benefit

Best Objective Response		Overall				Measurable Disease only			
		**Fulvestrant plus Placebo arm (n=71)**		**Fulvestrant plus AZD5363 arm (n=69)**		**Fulvestrant plus Placebo arm (n=50)**		**Fulvestrant plus AZD5363 arm (n=49)**	
Response	CR	0	0%	0	0%	0	0%	0	0%
	PR	6	8%	20	29%	6	12%	20	41%
Non-response	SD	43	61%	35	51%	27	54%	17	35%
	PD	21	30%	11	16%	17	34%	10	20%
	NE	1	1%	1	1%	0	0%	0	0%
	Missing	0	0%	2	3%	0	0%	2	4%
**Number of responders who subsequently progressed or died**		6	100%	14	70%				
**Median duration of response (months)***						5.03 (95% CI 2.66 - NR)		7.06 (95% CI 3.84 -9.92)	
**Overall Objective response**		6	8%	20	29%	6	12%	20	41%
Logistic Regression									
Odds Ratio		4.42 (95% CI 1.65-11.84); 2-sided p=0.0031				5.06 (95% CI 1.81-14.11); 2-sided p=0.0020			
**Median duration of ORR (months) (95% CI)**		5.03 (2.66, -)		7.06 (3.84, 9.92)					
**Clinical Benefit Rate (CBR)**		29	41%	38	55%	18	36%	27	55%
Logistic Regression									
Odds Ratio		1.78 (95% CI 0.91 - 3.47); 2-sided p= 0.093				2.18 (95% CI 0.97 - 4.89); 2-sided p=0.058			

***Duration of response is defined as the time from first documentation of complete or partial response to disease progression or death on study from any cause, whichever occurs first, in patient with objective response or to the last evaluable RECIST assessment for patients that do not progress/die.

Table 4 - Full toxicity listing

	**Fulvestrant plus Capivasertib arm (n=69)**										**Fulvestrant plus Placebo arm (n=71)**									
	**Grade Reported N**										**Grade Reported N**									
	**1**		**2**		**3**		**4**		**5**		**1**		**2**		**3**		**4**		**5**	
**Total number of AEs**	783		236		78		2		1		651		135		48		1		0	
**Patients with any AE**	69	(100%)	62	(90%)	45	(65%)	3	(4%)	1	(1%)	69	(99%)	50	(71%)	34	(49%)	1	(1%)	0	(0%)
**Worst grade of toxicity**	2	(3%)	22	(32%)	42	(61%)	2	(3%)	1	(1%)	12	(17%)	23	(33%)	34	(49%)	1	(1%)	0	(0%)
**Patients with AE leading to death**																				
Acute kidney injury (cause of death: atypical chest infection)	1	(1%)	0	(0%)	0	(0%)	0	(0%)	0	(0%)	0	(0%)	0	(0%)	0	(0%)	0	(0%)	0	(0%)
Haemorrhage	0	(0%)	0	(0%)	0	(0%)	0	(0%)	1	(1%)	0	(0%)	0	(0%)	0	(0%)	0	(0%)	0	(0%)
**Serious AEs**																				
Abdominal pain	0	(0%)	0	(0%)	1	(1%)	0	(0%)	0	(0%)	0	(0%)	1	(1%)	0	(0%)	0	(0%)	0	(0%)
Anaemia	0	(0%)	0	(0%)	0	(0%)	0	(0%)	0	(0%)	0	(0%)	0	(0%)	1	(1%)	0	(0%)	0	(0%)
Back pain	0	(0%)	0	(0%)	0	(0%)	0	(0%)	0	(0%)	0	(0%)	0	(0%)	2	(3%)	0	(0%)	0	(0%)
Blocked nephrostomy	0	(0%)	0	(0%)	1	(1%)	0	(0%)	0	(0%)	0	(0%)	0	(0%)	0	(0%)	0	(0%)	0	(0%)
Bone pain	0	(0%)	0	(0%)	1	(1%)	0	(0%)	0	(0%)	0	(0%)	0	(0%)	0	(0%)	0	(0%)	0	(0%)
Dyspnoea	0	(0%)	0	(0%)	0	(0%)	0	(0%)	0	(0%)	0	(0%)	1	(1%)	1	(1%)	1	(1%)	0	(0%)
Fever	0	(0%)	0	(0%)	0	(0%)	0	(0%)	0	(0%)	0	(0%)	0	(0%)	1	(1%)	0	(0%)	0	(0%)
Gastroenteritis	0	(0%)	0	(0%)	0	(0%)	0	(0%)	0	(0%)	0	(0%)	0	(0%)	1	(1%)	0	(0%)	0	(0%)
Haemorrhage	0	(0%)	0	(0%)	0	(0%)	0	(0%)	1	(1%)	0	(0%)	0	(0%)	0	(0%)	0	(0%)	0	(0%)
Hypercalcaemia	0	(0%)	0	(0%)	0	(0%)	0	(0%)	0	(0%)	1	(1%)	0	(0%)	0	(0%)	0	(0%)	0	(0%)
Infection	0	(0%)	0	(0%)	0	(0%)	0	(0%)	0	(0%)	0	(0%)	0	(0%)	1	(1%)	0	(0%)	0	(0%)
Lower respiratory tract infection	0	(0%)	0	(0%)	2	(3%)	0	(0%)	0	(0%)	0	(0%)	0	(0%)	1	(1%)	0	(0%)	0	(0%)
Muscle weakness lower limb	0	(0%)	0	(0%)	1	(1%)	0	(0%)	0	(0%)	0	(0%)	0	(0%)	0	(0%)	0	(0%)	0	(0%)
Non-cardiac chest pain	0	(0%)	0	(0%)	1	(1%)	0	(0%)	0	(0%)	1	(1%)	0	(0%)	0	(0%)	0	(0%)	0	(0%)
Pain	0	(0%)	0	(0%)	1	(1%)	0	(0%)	0	(0%)	0	(0%)	1	(1%)	1	(1%)	0	(0%)	0	(0%)

	Fulvestrant plus Capivasertib arm (n=69)										Fulvestrant plus Placebo arm (n=71)									
	**Grade Reported N**										**Grade Reported N**									
	**1**		**2**		**3**		**4**		**5**		**1**		**2**		**3**		**4**		**5**	
Pain in extremity	0	(0%)	0	(0%)	0	(0%)	0	(0%)	0	(0%)	0	(0%)	0	(0%)	1	(1%)	0	(0%)	0	(0%)
Perineal abscess	0	(0%)	0	(0%)	1	(1%)	0	(0%)	0	(0%)	0	(0%)	0	(0%)	0	(0%)	0	(0%)	0	(0%)
Pleural effusion	0	(0%)	0	(0%)	1	(1%)	0	(0%)	0	(0%)	0	(0%)	0	(0%)	0	(0%)	0	(0%)	0	(0%)
Radicular pain	0	(0%)	0	(0%)	0	(0%)	0	(0%)	0	(0%)	0	(0%)	0	(0%)	1	(1%)	0	(0%)	0	(0%)
Skin infection	0	(0%)	0	(0%)	1	(1%)	0	(0%)	0	(0%)	0	(0%)	0	(0%)	0	(0%)	0	(0%)	0	(0%)
Urinary tract infection	0	(0%)	0	(0%)	1	(1%)	0	(0%)	0	(0%)	0	(0%)	0	(0%)	0	(0%)	0	(0%)	0	(0%)
Vomiting	0	(0%)	0	(0%)	0	(0%)	0	(0%)	0	(0%)	0	(0%)	1	(1%)	0	(0%)	0	(0%)	0	(0%)
**Serious Adverse Reactions**																				
Acute kidney injury	1	(1%)	0	(0%)	1	(1%)	0	(0%)	0	(0%)	0	(0%)	0	(0%)	0	(0%)	0	(0%)	0	(0%)
Diarrhoea	0	(0%)	1	(1%)	2	(3%)	0	(0%)	0	(0%)	0	(0%)	0	(0%)	0	(0%)	0	(0%)	0	(0%)
Hyperglycaemia	0	(0%)	0	(0%)	1	(1%)	0	(0%)	0	(0%)	0	(0%)	0	(0%)	0	(0%)	0	(0%)	0	(0%)
Loss of consciousness	0	(0%)	1	(1%)	0	(0%)	0	(0%)	0	(0%)	0	(0%)	0	(0%)	0	(0%)	0	(0%)	0	(0%)
Rash	0	(0%)	0	(0%)	1	(1%)	0	(0%)	0	(0%)	0	(0%)	0	(0%)	0	(0%)	0	(0%)	0	(0%)
Rash maculo-papular	0	(0%)	0	(0%)	1	(1%)	0	(0%)	0	(0%)	0	(0%)	0	(0%)	0	(0%)	0	(0%)	0	(0%)
Sepsis	0	(0%)	0	(0%)	1	(1%)	0	(0%)	0	(0%)	0	(0%)	0	(0%)	0	(0%)	0	(0%)	0	(0%)
Vomiting	0	(0%)	1	(1%)	0	(0%)	0	(0%)	0	(0%)	0	(0%)	0	(0%)	0	(0%)	0	(0%)	0	(0%)
**All AES**																				
**Blood and lymphatic system disorders**																				
Anaemia	2	(3%)	1	(1%)	0	(0%)	0	(0%)	0	(0%)	0	(0%)	0	(0%)	2	(3%)	0	(0%)	0	(0%)
Febrile neutropenia	0	(0%)	0	(0%)	0	(0%)	0	(0%)	0	(0%)	0	(0%)	1	(1%)	0	(0%)	0	(0%)	0	(0%)
Haemoglobin decreased	1	(1%)	1	(1%)	0	(0%)	0	(0%)	0	(0%)	0	(0%)	0	(0%)	0	(0%)	0	(0%)	0	(0%)
Haemoglobin increased	0	(0%)	0	(0%)	0	(0%)	0	(0%)	0	(0%)	1	(1%)	0	(0%)	0	(0%)	0	(0%)	0	(0%)
Hyperglycaemia	17	(25%)	9	(13%)	3	(4%)	0	(0%)	0	(0%)	11	(16%)	0	(0%)	0	(0%)	0	(0%)	0	(0%)

	Fulvestrant plus Capivasertib arm (n=69)										Fulvestrant plus Placebo arm (n=71)									
	Grade Reported N										Grade Reported N									
	**1**		**2**		**3**		**4**		**5**		**1**		**2**		**3**		**4**		**5**	
Hypoglycaemia (fasting) (lab)	1	(1%)	0	(0%)	0	(0%)	0	(0%)	0	(0%)	0	(0%)	3	(4%)	0	(0%)	0	(0%)	0	(0%)
Hypoglycaemia (random) (lab)	2	(3%)	2	(3%)	0	(0%)	0	(0%)	0	(0%)	1	(1%)	0	(0%)	0	(0%)	0	(0%)	0	(0%)
Polycythaemia	0	(0%)	0	(0%)	0	(0%)	0	(0%)	0	(0%)	1	(1%)	0	(0%)	0	(0%)	0	(0%)	0	(0%)
Thrombocytopenia	0	(0%)	0	(0%)	0	(0%)	0	(0%)	0	(0%)	0	(0%)	0	(0%)	1	(1%)	0	(0%)	0	(0%)
**Cardiac disorders**																				
Palpitations	1	(1%)	0	(0%)	0	(0%)	0	(0%)	0	(0%)	0	(0%)	0	(0%)	0	(0%)	0	(0%)	0	(0%)
Pericardial effusion	0	(0%)	0	(0%)	0	(0%)	0	(0%)	0	(0%)	1	(1%)	0	(0%)	0	(0%)	0	(0%)	0	(0%)
Sinus tachycardia	0	(0%)	0	(0%)	0	(0%)	0	(0%)	0	(0%)	1	(1%)	0	(0%)	0	(0%)	0	(0%)	0	(0%)
**Congenital, familial and genetic disorders**																				
Genital candidiasis	0	(0%)	0	(0%)	0	(0%)	0	(0%)	0	(0%)	1	(1%)	0	(0%)	0	(0%)	0	(0%)	0	(0%)
**Ear and labyrinth disorders**																				
Ear infection	1	(1%)	1	(1%)	0	(0%)	0	(0%)	0	(0%)	0	(0%)	0	(0%)	0	(0%)	0	(0%)	0	(0%)
Ear pain	1	(1%)	0	(0%)	0	(0%)	0	(0%)	0	(0%)	1	(1%)	0	(0%)	0	(0%)	0	(0%)	0	(0%)
Tinnitus	1	(1%)	0	(0%)	0	(0%)	0	(0%)	0	(0%)	1	(1%)	0	(0%)	0	(0%)	0	(0%)	0	(0%)
Vertigo	0	(0%)	0	(0%)	0	(0%)	0	(0%)	0	(0%)	1	(1%)	0	(0%)	0	(0%)	0	(0%)	0	(0%)
**Eye disorders**																				
Blepharitis	0	(0%)	0	(0%)	0	(0%)	0	(0%)	0	(0%)	1	(1%)	0	(0%)	0	(0%)	0	(0%)	0	(0%)
Blurred vision	0	(0%)	0	(0%)	1	(1%)	0	(0%)	0	(0%)	1	(1%)	0	(0%)	0	(0%)	0	(0%)	0	(0%)
Cataract	1	(1%)	0	(0%)	0	(0%)	0	(0%)	0	(0%)	0	(0%)	0	(0%)	0	(0%)	0	(0%)	0	(0%)
Dry eye	0	(0%)	0	(0%)	0	(0%)	0	(0%)	0	(0%)	1	(1%)	0	(0%)	0	(0%)	0	(0%)	0	(0%)
Eye infection	0	(0%)	1	(1%)	0	(0%)	0	(0%)	0	(0%)	0	(0%)	0	(0%)	0	(0%)	0	(0%)	0	(0%)
Eye pain	2	(3%)	0	(0%)	0	(0%)	0	(0%)	0	(0%)	1	(1%)	0	(0%)	0	(0%)	0	(0%)	0	(0%)
Papilloedema	0	(0%)	0	(0%)	0	(0%)	0	(0%)	0	(0%)	1	(1%)	0	(0%)	0	(0%)	0	(0%)	0	(0%)
Photophobia	0	(0%)	0	(0%)	0	(0%)	0	(0%)	0	(0%)	0	(0%)	1	(1%)	0	(0%)	0	(0%)	0	(0%)
Scleral disorder	0	(0%)	0	(0%)	0	(0%)	0	(0%)	0	(0%)	0	(0%)	1	(1%)	0	(0%)	0	(0%)	0	(0%)

	Fulvestrant plus Capivasertib arm (n=69)										Fulvestrant plus Placebo arm (n=71)									
	**Grade Reported N**										**Grade Reported N**									
	**1**		**2**		**3**		**4**		**5**		**1**		**2**		**3**		**4**		**5**	
Watering eyes	1	(1%)	0	(0%)	0	(0%)	0	(0%)	0	(0%)	0	(0%)	0	(0%)	0	(0%)	0	(0%)	0	(0%)
**Gastrointestinal disorders**																				
Abdominal distension	2	(3%)	0	(0%)	0	(0%)	0	(0%)	0	(0%)	1	(1%)	0	(0%)	0	(0%)	0	(0%)	0	(0%)
Abdominal pain	9	(13%)	1	(1%)	0	(0%)	0	(0%)	0	(0%)	5	(7%)	1	(1%)	1	(1%)	0	(0%)	0	(0%)
Anal haemorrhage	1	(1%)	0	(0%)	0	(0%)	0	(0%)	0	(0%)	1	(1%)	0	(0%)	0	(0%)	0	(0%)	0	(0%)
Ascites	0	(0%)	0	(0%)	0	(0%)	0	(0%)	0	(0%)	0	(0%)	0	(0%)	1	(1%)	0	(0%)	0	(0%)
Belching	1	(1%)	0	(0%)	0	(0%)	0	(0%)	0	(0%)	0	(0%)	0	(0%)	0	(0%)	0	(0%)	0	(0%)
Constipation	6	(9%)	0	(0%)	0	(0%)	0	(0%)	0	(0%)	9	(13%)	0	(0%)	0	(0%)	0	(0%)	0	(0%)
Diarrhoea	28	(41%)	18	(26%)	10	(14%)	0	(0%)	0	(0%)	21	(30%)	1	(1%)	2	(3%)	1	(1%)	0	(0%)
Dry mouth	7	(10%)	0	(0%)	0	(0%)	0	(0%)	0	(0%)	3	(4%)	1	(1%)	0	(0%)	0	(0%)	0	(0%)
Duodenogastric reflux	0	(0%)	1	(1%)	0	(0%)	0	(0%)	0	(0%)	1	(1%)	0	(0%)	0	(0%)	0	(0%)	0	(0%)
Dyspepsia	3	(4%)	2	(3%)	0	(0%)	0	(0%)	0	(0%)	3	(4%)	2	(3%)	0	(0%)	0	(0%)	0	(0%)
Dysphagia	0	(0%)	0	(0%)	0	(0%)	0	(0%)	0	(0%)	0	(0%)	1	(1%)	0	(0%)	0	(0%)	0	(0%)
Epigastric discomfort	0	(0%)	0	(0%)	0	(0%)	0	(0%)	0	(0%)	1	(1%)	0	(0%)	0	(0%)	0	(0%)	0	(0%)
Flatulence	2	(3%)	0	(0%)	0	(0%)	0	(0%)	0	(0%)	0	(0%)	0	(0%)	0	(0%)	0	(0%)	0	(0%)
Gastroesophageal reflux disease	1	(1%)	0	(0%)	0	(0%)	0	(0%)	0	(0%)	1	(1%)	1	(1%)	0	(0%)	0	(0%)	0	(0%)
Gingival bleeding	0	(0%)	0	(0%)	0	(0%)	0	(0%)	0	(0%)	1	(1%)	0	(0%)	0	(0%)	0	(0%)	0	(0%)
Gingival blister	0	(0%)	1	(1%)	0	(0%)	0	(0%)	0	(0%)	0	(0%)	0	(0%)	0	(0%)	0	(0%)	0	(0%)
Gingival pain	1	(1%)	0	(0%)	0	(0%)	0	(0%)	0	(0%)	0	(0%)	0	(0%)	0	(0%)	0	(0%)	0	(0%)
Gingival recession	0	(0%)	1	(1%)	0	(0%)	0	(0%)	0	(0%)	0	(0%)	0	(0%)	0	(0%)	0	(0%)	0	(0%)
Glossitis	1	(1%)	0	(0%)	0	(0%)	0	(0%)	0	(0%)	0	(0%)	0	(0%)	0	(0%)	0	(0%)	0	(0%)
Heartburn	1	(1%)	0	(0%)	0	(0%)	0	(0%)	0	(0%)	1	(1%)	0	(0%)	0	(0%)	0	(0%)	0	(0%)
Lip pain	1	(1%)	0	(0%)	0	(0%)	0	(0%)	0	(0%)	0	(0%)	0	(0%)	0	(0%)	0	(0%)	0	(0%)
Mucositis oral	9	(13%)	1	(1%)	0	(0%)	0	(0%)	0	(0%)	5	(7%)	0	(0%)	0	(0%)	0	(0%)	0	(0%)
Nausea	30	(43%)	8	(12%)	0	(0%)	0	(0%)	0	(0%)	31	(44%)	5	(7%)	0	(0%)	0	(0%)	0	(0%)

	Fulvestrant plus Capivasertib arm (n=69)										Fulvestrant plus Placebo arm (n=71)									
	Grade Reported N										Grade Reported N									
	1		2		3		4		5		1		2		3		4		5	
Oral dysaesthesia	1	(1%)	0	(0%)	0	(0%)	0	(0%)	0	(0%)	0	(0%)	0	(0%)	0	(0%)	0	(0%)	0	(0%)
Oral pain	1	(1%)	0	(0%)	0	(0%)	0	(0%)	0	(0%)	1	(1%)	0	(0%)	0	(0%)	0	(0%)	0	(0%)
Oropharyngeal pain	3	(4%)	0	(0%)	0	(0%)	0	(0%)	0	(0%)	4	(6%)	0	(0%)	0	(0%)	0	(0%)	0	(0%)
Rhinorrhoea	1	(1%)	0	(0%)	0	(0%)	0	(0%)	0	(0%)	0	(0%)	0	(0%)	0	(0%)	0	(0%)	0	(0%)
Tooth repair	1	(1%)	0	(0%)	0	(0%)	0	(0%)	0	(0%)	0	(0%)	0	(0%)	0	(0%)	0	(0%)	0	(0%)
Toothache	0	(0%)	0	(0%)	0	(0%)	0	(0%)	0	(0%)	2	(3%)	0	(0%)	0	(0%)	0	(0%)	0	(0%)
Vomiting	17	(25%)	8	(12%)	2	(3%)	0	(0%)	0	(0%)	13	(19%)	2	(3%)	0	(0%)	0	(0%)	0	(0%)
**General disorders and administration site conditions**																				
Edema limbs	4	(6%)	0	(0%)	0	(0%)	0	(0%)	0	(0%)	2	(3%)	0	(0%)	0	(0%)	0	(0%)	0	(0%)
Facial pain	1	(1%)	0	(0%)	0	(0%)	0	(0%)	0	(0%)	0	(0%)	0	(0%)	0	(0%)	0	(0%)	0	(0%)
Fatigue	24	(35%)	15	(22%)	1	(1%)	0	(0%)	0	(0%)	26	(37%)	12	(17%)	3	(4%)	0	(0%)	0	(0%)
Fever	2	(3%)	1	(1%)	0	(0%)	0	(0%)	0	(0%)	0	(0%)	0	(0%)	1	(1%)	0	(0%)	0	(0%)
Flu like symptoms	6	(9%)	2	(3%)	0	(0%)	0	(0%)	0	(0%)	7	(10%)	2	(3%)	0	(0%)	0	(0%)	0	(0%)
Injection site reactions	16	(23%)	2	(3%)	1	(1%)	0	(0%)	0	(0%)	21	(30%)	2	(3%)	0	(0%)	0	(0%)	0	(0%)
Neck edema	1	(1%)	0	(0%)	0	(0%)	0	(0%)	0	(0%)	0	(0%)	0	(0%)	0	(0%)	0	(0%)	0	(0%)
Non-cardiac chest pain	5	(7%)	1	(1%)	1	(1%)	0	(0%)	0	(0%)	6	(9%)	0	(0%)	0	(0%)	0	(0%)	0	(0%)
Oedema	1	(1%)	0	(0%)	0	(0%)	0	(0%)	0	(0%)	0	(0%)	0	(0%)	0	(0%)	0	(0%)	0	(0%)
Oedema face	2	(3%)	0	(0%)	0	(0%)	0	(0%)	0	(0%)	0	(0%)	0	(0%)	0	(0%)	0	(0%)	0	(0%)
Pain	5	(7%)	1	(1%)	2	(3%)	0	(0%)	0	(0%)	5	(7%)	2	(3%)	0	(0%)	0	(0%)	0	(0%)
**Immune system disorders**																				
Hypersensitivity	1	(1%)	0	(0%)	0	(0%)	0	(0%)	0	(0%)	2	(3%)	0	(0%)	0	(0%)	0	(0%)	0	(0%)
**Infections and infestations**																				
Gastroenteritis	0	(0%)	0	(0%)	0	(0%)	0	(0%)	0	(0%)	0	(0%)	0	(0%)	1	(1%)	0	(0%)	0	(0%)
Infection	14	(20%)	10	(14%)	2	(3%)	0	(0%)	0	(0%)	7	(10%)	5	(7%)	1	(1%)	0	(0%)	0	(0%)

	Fulvestrant plus Capivasertib arm (n=69)										Fulvestrant plus Placebo arm (n=71)									
	**Grade Reported N**										**Grade Reported N**									
	**1**		**2**		**3**		**4**		**5**		**1**		**2**		**3**		**4**		**5**	
**Injury, poisoning and procedural complications**																				
Eye injury	0	(0%)	0	(0%)	0	(0%)	0	(0%)	0	(0%)	1	(1%)	0	(0%)	0	(0%)	0	(0%)	0	(0%)
Fracture	0	(0%)	0	(0%)	0	(0%)	0	(0%)	0	(0%)	1	(1%)	0	(0%)	1	(1%)	0	(0%)	0	(0%)
**Investigations**																				
Blood bilirubin decreased (lab)	1	(1%)	0	(0%)	0	(0%)	0	(0%)	0	(0%)	0	(0%)	0	(0%)	0	(0%)	0	(0%)	0	(0%)
Blood bilirubin increased (lab)	1	(1%)	2	(3%)	0	(0%)	0	(0%)	0	(0%)	1	(1%)	0	(0%)	0	(0%)	0	(0%)	0	(0%)
Cholesterol high	2	(3%)	0	(0%)	0	(0%)	0	(0%)	0	(0%)	2	(3%)	0	(0%)	0	(0%)	0	(0%)	0	(0%)
Cholesterol high (lab)	19	(28%)	4	(6%)	0	(0%)	0	(0%)	0	(0%)	16	(23%)	2	(3%)	0	(0%)	0	(0%)	0	(0%)
Creatinine decreased (lab)	2	(3%)	0	(0%)	0	(0%)	0	(0%)	0	(0%)	2	(3%)	0	(0%)	0	(0%)	0	(0%)	0	(0%)
Creatinine increased	3	(4%)	1	(1%)	0	(0%)	0	(0%)	0	(0%)	1	(1%)	0	(0%)	0	(0%)	0	(0%)	0	(0%)
Creatinine increased (lab)	8	(12%)	2	(3%)	0	(0%)	0	(0%)	0	(0%)	4	(6%)	0	(0%)	0	(0%)	0	(0%)	0	(0%)
ECHO ejection fraction (lab)	0	(0%)	0	(0%)	0	(0%)	0	(0%)	0	(0%)	0	(0%)	0	(0%)	0	(0%)	0	(0%)	0	(0%)
Electrocardiogram qt corrected interval prolonged	1	(1%)	0	(0%)	0	(0%)	0	(0%)	0	(0%)	1	(1%)	1	(1%)	0	(0%)	0	(0%)	0	(0%)
Electrocardiogram QT corrected interval prolonged (lab)	22	(32%)	2	(3%)	0	(0%)	0	(0%)	0	(0%)	16	(23%)	3	(4%)	0	(0%)	0	(0%)	0	(0%)
Elevated ALP	11	(16%)	5	(7%)	1	(1%)	0	(0%)	0	(0%)	13	(19%)	4	(6%)	2	(3%)	0	(0%)	0	(0%)
Elevated ALP (lab)	17	(25%)	2	(3%)	1	(1%)	0	(0%)	0	(0%)	19	(27%)	2	(3%)	1	(1%)	0	(0%)	0	(0%)
Elevated ALT	14	(20%)	2	(3%)	0	(0%)	0	(0%)	0	(0%)	11	(16%)	1	(1%)	2	(3%)	0	(0%)	0	(0%)
Elevated ALT (lab)	12	(17%)	2	(3%)	0	(0%)	0	(0%)	0	(0%)	10	(14%)	0	(0%)	1	(1%)	0	(0%)	0	(0%)
Elevated AST	6	(9%)	2	(3%)	0	(0%)	0	(0%)	0	(0%)	9	(13%)	1	(1%)	2	(3%)	0	(0%)	0	(0%)
Elevated AST (lab)	11	(16%)	0	(0%)	0	(0%)	0	(0%)	0	(0%)	6	(9%)	1	(1%)	0	(0%)	0	(0%)	0	(0%)
Elevated bilirubin	1	(1%)	1	(1%)	0	(0%)	0	(0%)	0	(0%)	2	(3%)	0	(0%)	0	(0%)	0	(0%)	0	(0%)
GGT increased	1	(1%)	0	(0%)	0	(0%)	0	(0%)	0	(0%)	0	(0%)	1	(1%)	0	(0%)	0	(0%)	0	(0%)

	**Fulvestrant plus Capivasertib arm (n=69)**										**Fulvestrant plus Placebo arm (n=71)**									
	**Grade Reported N**										**Grade Reported N**									
	**1**		**2**		**3**		**4**		**5**		**1**		**2**		**3**		**4**		**5**	
Glycosylated haemoglobin increased	2	(3%)	0	(0%)	0	(0%)	0	(0%)	0	(0%)	2	(3%)	0	(0%)	0	(0%)	0	(0%)	0	(0%)
Haemoglobin (lab)	14	(20%)	4	(6%)	0	(0%)	0	(0%)	0	(0%)	7	(10%)	5	(7%)	1	(1%)	0	(0%)	0	(0%)
HDL (lab)	14	(20%)	0	(0%)	0	(0%)	0	(0%)	0	(0%)	12	(17%)	0	(0%)	0	(0%)	0	(0%)	0	(0%)
Hypovitaminosis	1	(1%)	0	(0%)	0	(0%)	0	(0%)	0	(0%)	0	(0%)	0	(0%)	0	(0%)	0	(0%)	0	(0%)
LDL (lab)	14	(20%)	0	(0%)	0	(0%)	0	(0%)	0	(0%)	10	(14%)	0	(0%)	0	(0%)	0	(0%)	0	(0%)
Lymphocyte count decreased	1	(1%)	0	(0%)	0	(0%)	0	(0%)	0	(0%)	1	(1%)	0	(0%)	0	(0%)	0	(0%)	0	(0%)
Neutrophil count decreased	2	(3%)	0	(0%)	0	(0%)	0	(0%)	0	(0%)	0	(0%)	0	(0%)	0	(0%)	0	(0%)	0	(0%)
Neutrophils (high) (lab)	3	(4%)	0	(0%)	1	(1%)	0	(0%)	0	(0%)	3	(4%)	0	(0%)	0	(0%)	0	(0%)	0	(0%)
Neutrophils (low) (lab)	6	(9%)	1	(1%)	0	(0%)	0	(0%)	0	(0%)	8	(11%)	1	(1%)	0	(0%)	0	(0%)	0	(0%)
Platelet count decreased	0	(0%)	0	(0%)	0	(0%)	0	(0%)	0	(0%)	1	(1%)	0	(0%)	0	(0%)	0	(0%)	0	(0%)
Platelet count decreased (lab)	2	(3%)	0	(0%)	0	(0%)	1	(1%)	0	(0%)	4	(6%)	0	(0%)	0	(0%)	0	(0%)	0	(0%)
Platelet count increased (lab)	7	(10%)	0	(0%)	0	(0%)	0	(0%)	0	(0%)	1	(1%)	0	(0%)	0	(0%)	0	(0%)	0	(0%)
Prothrombin time (lab)	0	(0%)	0	(0%)	0	(0%)	0	(0%)	0	(0%)	0	(0%)	0	(0%)	0	(0%)	0	(0%)	0	(0%)
Pulse (high) (lab)	5	(7%)	0	(0%)	0	(0%)	0	(0%)	0	(0%)	9	(13%)	0	(0%)	0	(0%)	0	(0%)	0	(0%)
Pulse (low) (lab)	0	(0%)	0	(0%)	0	(0%)	0	(0%)	0	(0%)	1	(1%)	0	(0%)	0	(0%)	0	(0%)	0	(0%)
Urea (high) (lab)	12	(17%)	1	(1%)	0	(0%)	0	(0%)	0	(0%)	6	(9%)	0	(0%)	0	(0%)	0	(0%)	0	(0%)
Urea (low) (lab)	0	(0%)	0	(0%)	0	(0%)	0	(0%)	0	(0%)	1	(1%)	0	(0%)	0	(0%)	0	(0%)	0	(0%)
Weight loss	0	(0%)	2	(3%)	0	(0%)	0	(0%)	0	(0%)	1	(1%)	1	(1%)	0	(0%)	0	(0%)	0	(0%)
White blood cell count (high) (lab)	7	(10%)	3	(4%)	0	(0%)	0	(0%)	0	(0%)	7	(10%)	4	(6%)	0	(0%)	0	(0%)	0	(0%)
White blood cell count (low) (lab)	4	(6%)	0	(0%)	1	(1%)	0	(0%)	0	(0%)	2	(3%)	0	(0%)	0	(0%)	0	(0%)	0	(0%)
White blood cell count decreased	1	(1%)	1	(1%)	0	(0%)	0	(0%)	0	(0%)	1	(1%)	0	(0%)	0	(0%)	0	(0%)	0	(0%)

	Fulvestrant plus Capivasertib arm (n=69)										Fulvestrant plus Placebo arm (n=71)									
	Grade Reported N										Grade Reported N									
	**1**		**2**		**3**		**4**		**5**		**1**		**2**		**3**		**4**		**5**	
**Metabolism and nutrition disorders**																				
Anorexia	5	(7%)	3	(4%)	0	(0%)	0	(0%)	0	(0%)	2	(3%)	2	(3%)	0	(0%)	0	(0%)	0	(0%)
Dehydration	0	(0%)	0	(0%)	0	(0%)	0	(0%)	0	(0%)	0	(0%)	1	(1%)	0	(0%)	0	(0%)	0	(0%)
Hyperalbuminemia (lab)	1	(1%)	0	(0%)	0	(0%)	0	(0%)	0	(0%)	1	(1%)	0	(0%)	0	(0%)	0	(0%)	0	(0%)
Hyperammonaemia	1	(1%)	0	(0%)	0	(0%)	0	(0%)	0	(0%)	0	(0%)	0	(0%)	0	(0%)	0	(0%)	0	(0%)
Hypercalcaemia	1	(1%)	0	(0%)	0	(0%)	0	(0%)	0	(0%)	2	(3%)	0	(0%)	0	(0%)	0	(0%)	0	(0%)
Hypercalcemia (lab)	16	(23%)	1	(1%)	0	(0%)	0	(0%)	0	(0%)	11	(16%)	0	(0%)	0	(0%)	0	(0%)	0	(0%)
Hyperkalaemia	0	(0%)	1	(1%)	0	(0%)	0	(0%)	0	(0%)	0	(0%)	1	(1%)	0	(0%)	0	(0%)	0	(0%)
Hyperkalemia (lab)	7	(10%)	1	(1%)	0	(0%)	0	(0%)	0	(0%)	1	(1%)	1	(1%)	0	(0%)	0	(0%)	0	(0%)
Hypermagnesemia (lab)	0	(0%)	0	(0%)	0	(0%)	0	(0%)	0	(0%)	0	(0%)	0	(0%)	0	(0%)	0	(0%)	0	(0%)
Hypernatremia (lab)	7	(10%)	1	(1%)	0	(0%)	0	(0%)	0	(0%)	0	(0%)	0	(0%)	0	(0%)	0	(0%)	0	(0%)
Hypertriglyceridaemia	1	(1%)	1	(1%)	1	(1%)	0	(0%)	0	(0%)	1	(1%)	1	(1%)	0	(0%)	0	(0%)	0	(0%)
Hypertriglyceridemia (lab)	32	(46%)	4	(6%)	1	(1%)	0	(0%)	0	(0%)	18	(26%)	2	(3%)	0	(0%)	0	(0%)	0	(0%)
Hypoalbuminemia (lab)	7	(10%)	4	(6%)	0	(0%)	0	(0%)	0	(0%)	5	(7%)	1	(1%)	0	(0%)	0	(0%)	0	(0%)
Hypocalcaemia	2	(3%)	0	(0%)	0	(0%)	0	(0%)	0	(0%)	1	(1%)	0	(0%)	0	(0%)	0	(0%)	0	(0%)
Hypocalcemia (lab)	1	(1%)	1	(1%)	0	(0%)	1	(1%)	0	(0%)	0	(0%)	0	(0%)	0	(0%)	0	(0%)	0	(0%)
Hypokalemia (lab)	0	(0%)	0	(0%)	0	(0%)	0	(0%)	0	(0%)	0	(0%)	0	(0%)	0	(0%)	0	(0%)	0	(0%)
Hypomagnesemia (lab)	0	(0%)	0	(0%)	0	(0%)	0	(0%)	0	(0%)	0	(0%)	0	(0%)	0	(0%)	0	(0%)	0	(0%)
Hyponatraemia	1	(1%)	0	(0%)	0	(0%)	0	(0%)	0	(0%)	0	(0%)	0	(0%)	0	(0%)	0	(0%)	0	(0%)
Hyponatremia (lab)	1	(1%)	0	(0%)	2	(3%)	0	(0%)	0	(0%)	0	(0%)	0	(0%)	1	(1%)	0	(0%)	0	(0%)
Low triglycerides (lab)	2	(3%)	0	(0%)	0	(0%)	0	(0%)	0	(0%)	2	(3%)	0	(0%)	0	(0%)	0	(0%)	0	(0%)
**Musculoskeletal and connective tissue disorders**																				
Arthralgia	14	(20%)	5	(7%)	0	(0%)	0	(0%)	0	(0%)	18	(26%)	5	(7%)	0	(0%)	0	(0%)	0	(0%)
Arthritis	0	(0%)	0	(0%)	0	(0%)	0	(0%)	0	(0%)	1	(1%)	0	(0%)	0	(0%)	0	(0%)	0	(0%)

	Fulvestrant plus Capivasertib arm (n=69)										Fulvestrant plus Placebo arm (n=71)									
	Grade Reported N										Grade Reported N									
	1		2		3		4		5		1		2		3		4		5	
Back pain	12	(17%)	5	(7%)	0	(0%)	0	(0%)	0	(0%)	7	(10%)	4	(6%)	0	(0%)	0	(0%)	0	(0%)
Bone pain	2	(3%)	2	(3%)	0	(0%)	0	(0%)	0	(0%)	1	(1%)	0	(0%)	1	(1%)	0	(0%)	0	(0%)
Buttock pain	1	(1%)	2	(3%)	0	(0%)	0	(0%)	0	(0%)	1	(1%)	0	(0%)	0	(0%)	0	(0%)	0	(0%)
Carpal tunnel syndrome	1	(1%)	0	(0%)	0	(0%)	0	(0%)	0	(0%)	0	(0%)	0	(0%)	0	(0%)	0	(0%)	0	(0%)
Chest wall pain	1	(1%)	0	(0%)	0	(0%)	0	(0%)	0	(0%)	1	(1%)	1	(1%)	0	(0%)	0	(0%)	0	(0%)
Dysesthesia	0	(0%)	0	(0%)	0	(0%)	0	(0%)	0	(0%)	2	(3%)	0	(0%)	0	(0%)	0	(0%)	0	(0%)
Flank pain	0	(0%)	0	(0%)	0	(0%)	0	(0%)	0	(0%)	1	(1%)	0	(0%)	0	(0%)	0	(0%)	0	(0%)
Jaw pain	1	(1%)	0	(0%)	0	(0%)	0	(0%)	0	(0%)	1	(1%)	0	(0%)	0	(0%)	0	(0%)	0	(0%)
Muscle spasms	6	(9%)	0	(0%)	0	(0%)	0	(0%)	0	(0%)	3	(4%)	1	(1%)	0	(0%)	0	(0%)	0	(0%)
Muscle weakness lower limb	0	(0%)	0	(0%)	1	(1%)	0	(0%)	0	(0%)	0	(0%)	0	(0%)	0	(0%)	0	(0%)	0	(0%)
Muscle weakness upper limb	0	(0%)	0	(0%)	0	(0%)	0	(0%)	0	(0%)	1	(1%)	0	(0%)	0	(0%)	0	(0%)	0	(0%)
Myalgia	0	(0%)	0	(0%)	0	(0%)	0	(0%)	0	(0%)	1	(1%)	1	(1%)	0	(0%)	0	(0%)	0	(0%)
Neck pain	4	(6%)	0	(0%)	0	(0%)	0	(0%)	0	(0%)	4	(6%)	0	(0%)	0	(0%)	0	(0%)	0	(0%)
Osteonecrosis of jaw	2	(3%)	1	(1%)	0	(0%)	0	(0%)	0	(0%)	0	(0%)	0	(0%)	0	(0%)	0	(0%)	0	(0%)
Pain in extremity	7	(10%)	2	(3%)	0	(0%)	0	(0%)	0	(0%)	6	(9%)	2	(3%)	2	(3%)	0	(0%)	0	(0%)
Pelvic pain	0	(0%)	0	(0%)	0	(0%)	0	(0%)	0	(0%)	0	(0%)	1	(1%)	0	(0%)	0	(0%)	0	(0%)
Restless legs syndrome	0	(0%)	0	(0%)	0	(0%)	0	(0%)	0	(0%)	1	(1%)	0	(0%)	0	(0%)	0	(0%)	0	(0%)
**Neoplasms benign, malignant and unspecified (incl cysts and polyps)**																				
Cyst	0	(0%)	1	(1%)	0	(0%)	0	(0%)	0	(0%)	0	(0%)	0	(0%)	0	(0%)	0	(0%)	0	(0%)
Lymphadenopathy	0	(0%)	0	(0%)	0	(0%)	0	(0%)	0	(0%)	1	(1%)	0	(0%)	0	(0%)	0	(0%)	0	(0%)
Tumour pain	1	(1%)	0	(0%)	0	(0%)	0	(0%)	0	(0%)	0	(0%)	0	(0%)	0	(0%)	0	(0%)	0	(0%)
**Nervous system disorders**																				
Amnesia	1	(1%)	0	(0%)	0	(0%)	0	(0%)	0	(0%)	1	(1%)	0	(0%)	0	(0%)	0	(0%)	0	(0%)
Aphonia	0	(0%)	0	(0%)	0	(0%)	0	(0%)	0	(0%)	1	(1%)	0	(0%)	0	(0%)	0	(0%)	0	(0%)

	Fulvestrant plus Capivasertib arm (n=69)										Fulvestrant plus Placebo arm (n=71)									
	**Grade Reported N**										**Grade Reported N**									
	**1**		**2**		**3**		**4**		**5**		**1**		**2**		**3**		**4**		**5**	
Asthenopia	0	(0%)	0	(0%)	0	(0%)	0	(0%)	0	(0%)	1	(1%)	0	(0%)	0	(0%)	0	(0%)	0	(0%)
Burning sensation	1	(1%)	0	(0%)	0	(0%)	0	(0%)	0	(0%)	0	(0%)	0	(0%)	0	(0%)	0	(0%)	0	(0%)
Dizziness	7	(10%)	0	(0%)	0	(0%)	0	(0%)	0	(0%)	1	(1%)	0	(0%)	0	(0%)	0	(0%)	0	(0%)
Dysaesthesia	1	(1%)	0	(0%)	0	(0%)	0	(0%)	0	(0%)	0	(0%)	0	(0%)	0	(0%)	0	(0%)	0	(0%)
Dysgeusia	4	(6%)	0	(0%)	0	(0%)	0	(0%)	0	(0%)	1	(1%)	0	(0%)	0	(0%)	0	(0%)	0	(0%)
Headache	16	(23%)	1	(1%)	0	(0%)	0	(0%)	0	(0%)	19	(27%)	4	(6%)	0	(0%)	0	(0%)	0	(0%)
Hypoesthesia	0	(0%)	0	(0%)	0	(0%)	0	(0%)	0	(0%)	1	(1%)	0	(0%)	0	(0%)	0	(0%)	0	(0%)
Lethargy	3	(4%)	1	(1%)	0	(0%)	0	(0%)	0	(0%)	0	(0%)	0	(0%)	0	(0%)	0	(0%)	0	(0%)
Mental impairment	0	(0%)	0	(0%)	0	(0%)	0	(0%)	0	(0%)	1	(1%)	0	(0%)	0	(0%)	0	(0%)	0	(0%)
Paraesthesia	4	(6%)	0	(0%)	0	(0%)	0	(0%)	0	(0%)	4	(6%)	0	(0%)	0	(0%)	0	(0%)	0	(0%)
Peripheral motor neuropathy	0	(0%)	0	(0%)	1	(1%)	0	(0%)	0	(0%)	0	(0%)	0	(0%)	0	(0%)	0	(0%)	0	(0%)
Peripheral neuropathy	2	(3%)	0	(0%)	1	(1%)	0	(0%)	0	(0%)	2	(3%)	0	(0%)	0	(0%)	0	(0%)	0	(0%)
Presyncope	1	(1%)	0	(0%)	0	(0%)	0	(0%)	0	(0%)	0	(0%)	0	(0%)	0	(0%)	0	(0%)	0	(0%)
Radicular pain	0	(0%)	0	(0%)	0	(0%)	0	(0%)	0	(0%)	1	(1%)	0	(0%)	1	(1%)	0	(0%)	0	(0%)
Sciatica	0	(0%)	1	(1%)	1	(1%)	0	(0%)	0	(0%)	1	(1%)	2	(3%)	0	(0%)	0	(0%)	0	(0%)
Sensory disturbance	0	(0%)	0	(0%)	0	(0%)	0	(0%)	0	(0%)	1	(1%)	0	(0%)	0	(0%)	0	(0%)	0	(0%)
Spinal disorder	0	(0%)	1	(1%)	0	(0%)	0	(0%)	0	(0%)	0	(0%)	0	(0%)	0	(0%)	0	(0%)	0	(0%)
Syncope	0	(0%)	0	(0%)	0	(0%)	0	(0%)	0	(0%)	0	(0%)	0	(0%)	1	(1%)	0	(0%)	0	(0%)
Tremor	1	(1%)	0	(0%)	0	(0%)	0	(0%)	0	(0%)	0	(0%)	0	(0%)	0	(0%)	0	(0%)	0	(0%)
**Psychiatric disorders**																				
Anxiety	0	(0%)	0	(0%)	0	(0%)	0	(0%)	0	(0%)	2	(3%)	1	(1%)	0	(0%)	0	(0%)	0	(0%)
Depression	1	(1%)	2	(3%)	0	(0%)	0	(0%)	0	(0%)	4	(6%)	0	(0%)	0	(0%)	0	(0%)	0	(0%)
Insomnia	4	(6%)	0	(0%)	0	(0%)	0	(0%)	0	(0%)	5	(7%)	1	(1%)	0	(0%)	0	(0%)	0	(0%)
**Renal and urinary disorders**																				
Acute kidney injury	2	(3%)	1	(1%)	1	(1%)	0	(0%)	0	(0%)	1	(1%)	0	(0%)	0	(0%)	0	(0%)	0	(0%)
Blocked nephrostomy	0	(0%)	0	(0%)	1	(1%)	0	(0%)	0	(0%)	0	(0%)	0	(0%)	0	(0%)	0	(0%)	0	(0%)

	Fulvestrant plus Capivasertib arm (n=69)										Fulvestrant plus Placebo arm (n=71)									
	**Grade Reported N**										**Grade Reported N**									
	**1**		**2**		**3**		**4**		**5**		**1**		**2**		**3**		**4**		**5**	
Cystitis	0	(0%)	3	(4%)	0	(0%)	0	(0%)	0	(0%)	0	(0%)	0	(0%)	0	(0%)	0	(0%)	0	(0%)
Cystitis noninfective	1	(1%)	0	(0%)	0	(0%)	0	(0%)	0	(0%)	0	(0%)	0	(0%)	0	(0%)	0	(0%)	0	(0%)
Dysuria	1	(1%)	1	(1%)	0	(0%)	0	(0%)	0	(0%)	1	(1%)	0	(0%)	0	(0%)	0	(0%)	0	(0%)
Glomerular filtration rate decreased	0	(0%)	1	(1%)	0	(0%)	0	(0%)	0	(0%)	0	(0%)	0	(0%)	0	(0%)	0	(0%)	0	(0%)
Glycosuria	1	(1%)	0	(0%)	0	(0%)	0	(0%)	0	(0%)	0	(0%)	0	(0%)	0	(0%)	0	(0%)	0	(0%)
Haematuria	2	(3%)	0	(0%)	0	(0%)	0	(0%)	0	(0%)	0	(0%)	0	(0%)	0	(0%)	0	(0%)	0	(0%)
Haemoglobinuria	0	(0%)	0	(0%)	0	(0%)	0	(0%)	0	(0%)	1	(1%)	0	(0%)	0	(0%)	0	(0%)	0	(0%)
Hypertonic bladder	0	(0%)	0	(0%)	0	(0%)	0	(0%)	0	(0%)	1	(1%)	0	(0%)	0	(0%)	0	(0%)	0	(0%)
Polyuria	1	(1%)	0	(0%)	0	(0%)	0	(0%)	0	(0%)	0	(0%)	0	(0%)	0	(0%)	0	(0%)	0	(0%)
Proteinuria	2	(3%)	0	(0%)	0	(0%)	0	(0%)	0	(0%)	0	(0%)	0	(0%)	0	(0%)	0	(0%)	0	(0%)
Proteinuria (lab)	17	(25%)	8	(12%)	0	(0%)	0	(0%)	0	(0%)	8	(11%)	0	(0%)	0	(0%)	0	(0%)	0	(0%)
Urinary frequency	2	(3%)	0	(0%)	0	(0%)	0	(0%)	0	(0%)	0	(0%)	0	(0%)	0	(0%)	0	(0%)	0	(0%)
Urine glycolic acid increased	0	(0%)	1	(1%)	0	(0%)	0	(0%)	0	(0%)	0	(0%)	0	(0%)	0	(0%)	0	(0%)	0	(0%)
**Reproductive system and breast disorders**																				
Breast oedema	1	(1%)	0	(0%)	0	(0%)	0	(0%)	0	(0%)	1	(1%)	0	(0%)	0	(0%)	0	(0%)	0	(0%)
Breast pain	2	(3%)	0	(0%)	0	(0%)	0	(0%)	0	(0%)	1	(1%)	1	(1%)	0	(0%)	0	(0%)	0	(0%)
Dysmenorrhoea	0	(0%)	0	(0%)	0	(0%)	0	(0%)	0	(0%)	1	(1%)	0	(0%)	0	(0%)	0	(0%)	0	(0%)
Perineal abscess	0	(0%)	0	(0%)	1	(1%)	0	(0%)	0	(0%)	0	(0%)	0	(0%)	0	(0%)	0	(0%)	0	(0%)
Uterine pain	0	(0%)	0	(0%)	0	(0%)	0	(0%)	0	(0%)	1	(1%)	0	(0%)	0	(0%)	0	(0%)	0	(0%)
Vaginal discharge	1	(1%)	0	(0%)	0	(0%)	0	(0%)	0	(0%)	0	(0%)	0	(0%)	0	(0%)	0	(0%)	0	(0%)
Vaginal haemorrhage	0	(0%)	1	(1%)	0	(0%)	0	(0%)	0	(0%)	2	(3%)	0	(0%)	0	(0%)	0	(0%)	0	(0%)
**Respiratory, thoracic and mediastinal disorders**																				
Asthma	0	(0%)	2	(3%)	0	(0%)	0	(0%)	0	(0%)	0	(0%)	0	(0%)	0	(0%)	0	(0%)	0	(0%)

	Fulvestrant plus Capivasertib arm (n=69)										Fulvestrant plus Placebo arm (n=71)									
	**Grade Reported N**										**Grade Reported N**									
	**1**		**2**		**3**		**4**		**5**		**1**		**2**		**3**		**4**		**5**	
Cough	5	(7%)	2	(3%)	0	(0%)	0	(0%)	0	(0%)	5	(7%)	2	(3%)	0	(0%)	0	(0%)	0	(0%)
Dysphonia	1	(1%)	0	(0%)	0	(0%)	0	(0%)	0	(0%)	3	(4%)	0	(0%)	0	(0%)	0	(0%)	0	(0%)
Dyspnoea	2	(3%)	3	(4%)	0	(0%)	0	(0%)	0	(0%)	9	(13%)	3	(4%)	1	(1%)	0	(0%)	0	(0%)
Emphysema	0	(0%)	0	(0%)	0	(0%)	0	(0%)	0	(0%)	1	(1%)	0	(0%)	0	(0%)	0	(0%)	0	(0%)
Epistaxis	1	(1%)	0	(0%)	0	(0%)	0	(0%)	0	(0%)	1	(1%)	0	(0%)	0	(0%)	0	(0%)	0	(0%)
Hypoxia	0	(0%)	0	(0%)	1	(1%)	0	(0%)	0	(0%)	0	(0%)	0	(0%)	0	(0%)	0	(0%)	0	(0%)
Hypoxia (lab)	0	(0%)	0	(0%)	0	(0%)	0	(0%)	0	(0%)	2	(3%)	0	(0%)	0	(0%)	0	(0%)	0	(0%)
Nasal congestion	1	(1%)	0	(0%)	0	(0%)	0	(0%)	0	(0%)	2	(3%)	0	(0%)	0	(0%)	0	(0%)	0	(0%)
Pleural effusion	1	(1%)	0	(0%)	1	(1%)	0	(0%)	0	(0%)	1	(1%)	0	(0%)	0	(0%)	0	(0%)	0	(0%)
Postnasal drip	2	(3%)	0	(0%)	0	(0%)	0	(0%)	0	(0%)	1	(1%)	0	(0%)	0	(0%)	0	(0%)	0	(0%)
Respiratory	0	(0%)	0	(0%)	0	(0%)	0	(0%)	0	(0%)	0	(0%)	0	(0%)	1	(1%)	0	(0%)	0	(0%)
**Skin and subcutaneous tissue disorders**																				
Alopecia	3	(4%)	1	(1%)	0	(0%)	0	(0%)	0	(0%)	1	(1%)	0	(0%)	0	(0%)	0	(0%)	0	(0%)
Blister	1	(1%)	0	(0%)	0	(0%)	0	(0%)	0	(0%)	0	(0%)	0	(0%)	0	(0%)	0	(0%)	0	(0%)
Chronic lipodermatosclerosis	1	(1%)	0	(0%)	0	(0%)	0	(0%)	0	(0%)	0	(0%)	0	(0%)	0	(0%)	0	(0%)	0	(0%)
Contusion	1	(1%)	0	(0%)	0	(0%)	0	(0%)	0	(0%)	0	(0%)	0	(0%)	0	(0%)	0	(0%)	0	(0%)
Dry skin	7	(10%)	0	(0%)	0	(0%)	0	(0%)	0	(0%)	2	(3%)	0	(0%)	0	(0%)	0	(0%)	0	(0%)
Eczema	1	(1%)	0	(0%)	0	(0%)	0	(0%)	0	(0%)	0	(0%)	0	(0%)	0	(0%)	0	(0%)	0	(0%)
Erythema	2	(3%)	1	(1%)	0	(0%)	0	(0%)	0	(0%)	1	(1%)	0	(0%)	0	(0%)	0	(0%)	0	(0%)
Excoriation	0	(0%)	0	(0%)	0	(0%)	0	(0%)	0	(0%)	1	(1%)	0	(0%)	0	(0%)	0	(0%)	0	(0%)
Infected bites	2	(3%)	0	(0%)	0	(0%)	0	(0%)	0	(0%)	0	(0%)	0	(0%)	0	(0%)	0	(0%)	0	(0%)
Onychoclasis	5	(7%)	0	(0%)	0	(0%)	0	(0%)	0	(0%)	0	(0%)	0	(0%)	0	(0%)	0	(0%)	0	(0%)
Open wound	1	(1%)	0	(0%)	0	(0%)	0	(0%)	0	(0%)	0	(0%)	0	(0%)	0	(0%)	0	(0%)	0	(0%)
Pain of skin	0	(0%)	0	(0%)	0	(0%)	0	(0%)	0	(0%)	1	(1%)	0	(0%)	0	(0%)	0	(0%)	0	(0%)

	Fulvestrant plus Capivasertib arm (n=69)										Fulvestrant plus Placebo arm (n=71)									
	**Grade Reported N**										**Grade Reported N**									
	**1**		**2**		**3**		**4**		**5**		**1**		**2**		**3**		**4**		**5**	
Palmar-plantar erythrodysaesthesia syndrome	1	(1%)	1	(1%)	0	(0%)	0	(0%)	0	(0%)	0	(0%)	0	(0%)	0	(0%)	0	(0%)	0	(0%)
Pruritus	6	(9%)	1	(1%)	0	(0%)	0	(0%)	0	(0%)	5	(7%)	0	(0%)	0	(0%)	0	(0%)	0	(0%)
Psoriasis	0	(0%)	0	(0%)	0	(0%)	0	(0%)	0	(0%)	0	(0%)	1	(1%)	0	(0%)	0	(0%)	0	(0%)
Rash	15	(22%)	7	(10%)	14	(20%)	0	(0%)	0	(0%)	11	(16%)	2	(3%)	0	(0%)	0	(0%)	0	(0%)
Skin laceration	0	(0%)	0	(0%)	0	(0%)	0	(0%)	0	(0%)	1	(1%)	0	(0%)	0	(0%)	0	(0%)	0	(0%)
Skin ulceration	1	(1%)	0	(0%)	0	(0%)	0	(0%)	0	(0%)	0	(0%)	0	(0%)	0	(0%)	0	(0%)	0	(0%)
**Surgical and medical procedures**																				
Dental care	0	(0%)	1	(1%)	0	(0%)	0	(0%)	0	(0%)	0	(0%)	0	(0%)	0	(0%)	0	(0%)	0	(0%)
Failure of implant	0	(0%)	0	(0%)	0	(0%)	0	(0%)	0	(0%)	1	(1%)	0	(0%)	0	(0%)	0	(0%)	0	(0%)
**Vascular disorders**																				
Flushing	1	(1%)	0	(0%)	0	(0%)	0	(0%)	0	(0%)	1	(1%)	0	(0%)	0	(0%)	0	(0%)	0	(0%)
Haemorrhage	0	(0%)	0	(0%)	0	(0%)	0	(0%)	1	(1%)	0	(0%)	0	(0%)	0	(0%)	0	(0%)	0	(0%)
Hot flashes	8	(12%)	2	(3%)	0	(0%)	0	(0%)	0	(0%)	12	(17%)	0	(0%)	0	(0%)	0	(0%)	0	(0%)
Hypertension	1	(1%)	1	(1%)	0	(0%)	0	(0%)	0	(0%)	1	(1%)	0	(0%)	0	(0%)	0	(0%)	0	(0%)
Hypertension (lab)	20	(29%)	21	(30%)	22	(32%)	0	(0%)	0	(0%)	23	(33%)	22	(31%)	17	(24%)	0	(0%)	0	(0%)
Hypotension	0	(0%)	0	(0%)	0	(0%)	0	(0%)	0	(0%)	1	(1%)	0	(0%)	0	(0%)	0	(0%)	0	(0%)
Hypotension (lab)	0	(0%)	0	(0%)	0	(0%)	0	(0%)	0	(0%)	2	(3%)	0	(0%)	0	(0%)	0	(0%)	0	(0%)
Loss of consciousness	0	(0%)	0	(0%)	0	(0%)	0	(0%)	0	(0%)	0	(0%)	1	(1%)	0	(0%)	0	(0%)	0	(0%)
Lymphoedema	1	(1%)	2	(3%)	0	(0%)	0	(0%)	0	(0%)	1	(1%)	1	(1%)	0	(0%)	0	(0%)	0	(0%)
Thromboembolic event	1	(1%)	0	(0%)	0	(0%)	0	(0%)	0	(0%)	0	(0%)	0	(0%)	0	(0%)	0	(0%)	0	(0%)

*AEs marked with (lab) were toxicities reported as abnormal blood/biochemistry results and may not have had clinical significance.

Table 5- Pharmacokinetic analysis of mean minimum (Cmin) fulvestrant concentration (ng/ml)

**Cycle and Day**	**Placebo**					**Capivasertib**				
	Mean Log Cmin	Geometric mean Cmin	95% CI (Geometric mean Cmin)		n	Mean	Geometric mean Cmin	95% CI (Geometric mean Cmin)		n
**C1 D15**	2.27	9.64	8.87	10.46	60	2.25	9.47	8.54	10.49	56
**C2 D1**	2.58	13.26	12.08	14.48	57	2.68	14.57	13.34	15.92	56
**C3 D1**	2.34	10.34	9.60	11.13	48	2.35	10.46	9.63	11.36	46

Table 6- FAKTION Recruiting Centres

Site	PI	Number of Participants registered	Number of Participants randomised
The Christie NHS Foundation Trust, Manchester	Dr Sacha Howell	33	32
Velindre Cancer Centre, Velindre University NHS Trust, Cardiff	Dr Simon Waters	26	26
The Leeds Teaching Hospitals NHS Trust, St James's University Hospital, Leeds	Prof Chris Twelves	16	16
Calderdale and Huddersfield NHS Foundation Trust, Huddersfield	Prof Jonathan Joffe	12	11
University Hospitals of Morecambe Bay NHS Foundation Trust, Lancaster	Dr Sarah Moon	8	8
Betsi Cadwaladr University Health Board, Ysbyty Gwynedd, Bangor	Dr Catherine Bale	6	6
The Ipswich Hospital NHS Trust	Dr Ramachandran Venkitaraman	6	6
Blackpool Teaching Hospitals NHS Foundation Trust, Blackpool Victoria Hospital, Blackpool	Dr Pavel Bezecny	6	5
Barking, Havering and Redbridge University Hospitals NHS Trust, Queen's Hospital, Romford	Dr Mary Quigley	4	4
Wrightington, Wigan and Leigh NHS Foundation Trust, Royal Albert and Edward Infirmary, Wigan	Dr Elena Takeuchi	4	4
Betsi Cadwaladr University Health Board, Glan Clwyd Hospital, Bodelwyddan, Rhyl	Dr Jill Bishop	4	4
The Clatterbridge Cancer Centre NHS Foundation Trust, Clatterbridge Cancer Centre, Liverpool	Prof Carlo Palmieri	4	4
Plymouth Hospitals NHS Trust, Derriford Hospital, Derriford	Dr Sidharth Dubey	4	4
University Hospitals Southampton NHS Foundation Trust, Southampton General Hospital, Southampton	Dr Ellen Copson	4	3
Lancashire Teaching Hospitals NHS Foundation Trust, Royal Preston Hospital, Preston	Dr Elaine Young	3	3
Royal Free London NHS Foundation Trust, Royal Free Hospital, London	Dr Jackie Newby	2	2
East Lancashire Hospitals NHS Trust, Royal Blackburn Hospital, Blackburn	Dr Martin Hogg	1	1
County Durham and Darlington NHS Foundation Trust, University Hospital North Durham, Durham	Dr Wendy Taylor	1	1
University Hospitals of North Midlands NHS Trust, Royal Stoke University Hospital, Stoke-on-Trent	Prof Murray Brunt	1	0

FAKTION CLINICAL TRIAL PROTOCOL

Short Title: Fulvestrant +/- Akt inhibition in advanced aromatase inhibitor resistant breast cancer

Full Title: A phase 1b/2 randomised placebo controlled trial of fulvestrant +/- AZD5363 in postmenopausal women with advanced breast cancer previously treated with a third generation aromatase inhibitor

**Version:**	[redacted]
**Date:**	[redacted]
**EudraCT No:**	**2013-000898-68**
**ClinicalTrials.gov**	**NCT01992952**
**Funder:**	**AstraZeneca and Cancer Research UK (an NCRN/AstraZeneca collaborative trial)**
**CRUK Ref No:**	**CRUK/12/044**
**Name of Sponsor:**	**Velindre NHS Trust**
**Sponsor No:**	**2013/VCC/0008**

Authorised by:

**Name:**	[redacted]	**Role:**	[redacted]
**Signature:**	[redacted]	**Date:**	[redacted]
**Name:**	[redacted]	**Role:**	[redacted]
**Signature:**	[redacted]	**Date:**	[redacted]
**Name:**	[redacted]	**Role:**	[redacted]
**Signature:**	[redacted]	**Date:**	[redacted]

Developed on behalf of the NCRI Breast Clinical Studies Group

General Information

This protocol describes the FAKTION clinical trial, and provides information about the procedures for entering participants into the trial. The protocol should not be used as a guide, or as an aide-memoire for the treatment of other patients. Every care has been taken in drafting this protocol; however, corrections or amendments may be necessary. These will be circulated to the known Investigators in the trial, but centres entering patients for the first time are advised to contact the Wales Cancer Trials Unit (WCTU) [redacted] to confirm that they have the most up-to-date version of the protocol in their possession. Problems relating to the trial should be referred, in the first instance, to the WCTU.

Compliance

This trial will adhere to the conditions and principles which apply to all clinical trials as outlined in the Medicines for Human Use (Clinical Trials) Regulations 2004, as amended, EU Directive 2001/20/EC, EU Directive 2005/28/EC and the ICH Harmonised Tripartite Guideline for Good Clinical Practice (CPMP/ICH/135/95). It will be conducted in compliance with the protocol, the Medicines for Human Use (Clinical Trials) Regulations 2004 (SI2004/1031), as amended, the Research Governance Framework for Health and Social Care (Welsh Assembly Government November 2001 and Department of Health 2nd July 2005), the Data Protection Act 1998, and other regulatory requirements as appropriate.

Funding

The FAKTION trial is being funded by the AstraZeneca NCRN collaboration and has been approved and additionally funded by the CR-UK Feasibility Study Committee and is thus part of the NCRN/NCRI portfolio of clinical trials. This study is supported by Cancer Research UK core funding at the WCTU.

[redacted]

Trial Co-ordination

The FAKTION trial is being coordinated by the Wales Cancer Trials Unit (WCTU), a National Cancer Research Institute (NCRI) accredited and United Kingdom Clinical Research Collaboration (UKCRC) registered trials unit, which is part of the Cardiff University Centre for Trials Research (CTR).

[redacted]

[redacted]

[redacted]

[redacted]

[redacted]

[redacted]	[redacted]	[redacted]
[redacted]	[redacted]	[redacted]
[redacted]	[redacted]	[redacted]
[redacted]	[redacted]	[redacted]
[redacted]	[redacted]	[redacted]
[redacted]		[redacted]

Chief Investigators

[redacted]

[redacted]

Co-investigators

[redacted]

[redacted]

[redacted]

[redacted]

[redacted]

[redacted]

[redacted]

[redacted]

[redacted]

[redacted]

[redacted]

[redacted]

[redacted] [redacted]

Table of contents

1.0 Trial schema 10
2.0 Trial synopsis 10
2.1 Lay summary 15
3.0 Background 16
3.1 Investigational agent 16
3.2 Non-clinical information and correlative studies 16
3.3 Clinical information 17
3.4 Pharmacokinetics 19
3.5 Research hypotheses 20
3.6 Rationale for conducting this study 20
3.6.1 Estrogen Receptor Positive Metastatic Breast Cancer - Background 20
3.6.2 Rationale for using fulvestrant as the endocrine backbone 21
3.6.3 Rationale for using AZD5363 in advanced endocrine resistant ER+BC 21
4.0 Study design 23
4.1 Risk assessment 24
4.2 Phase 2 (Stages 2-4) 25
4.2.1 Safety review 25
4.2.2 Activity review within stratified PIK3CA groups 25
4.3 Completion of Treatment 25
5.0 Participating centre selection 27
6.0 Participant eligibility 28
6.1 Informed consent 28
6.2 Screening procedures 29
6.2.1 Inclusion criteria 30
6.2.2 Exclusion criteria 32
6.3 Restrictions 34
6.4 Registration and randomisation 35
6.4.1 Phase 2 (Stages 2, 3 and 4) 35
6.4.2 Treatment pack allocation using the IWRS website 36
7.0 Trial treatments 38
7.1 Treatment Schedules 38
7.2 Dose delays and modifications 39
7.3 Management of toxicities 39
7.4 Drug supply and distribution 40

7.5 Drug accountability 41
Storage 41
Accountability 41
7.6 Concomitant medications 42
7.7 Use of metformin 45
7.8 Unblinding procedure 45
8.1 Schedule of trial treatments and assessments 46
8.2 Completion of CRFs 48
9.0 Safety reporting and pharmacovigilance 49
9.1 Causality Assessments 50
9.2 Expectedness Assessments 50
9.3 SAE reporting 51
9.3.2 The WCTU responsibilities 52
9.4 SUSAR reporting 52
9.5 Unblinding for the purposes of SUSAR reporting 53
9.6 Safety Reports 53
9.7 Flowchart for Serious Adverse Event reporting 54
10.0 Trial monitoring and management 55
10.1 Monitoring 55
10.1.1 Central monitoring 55
10.1.2 Site monitoring 55
10.2 Trial committees and trial management 55
10.3 End of treatment and participant withdrawal 56
10.4 Lost to follow up 57
10.5 Protocol/GCP non-compliance 57
10.6 Trial closure 57
10.7 Archiving 57
11.0 Statistical considerations 58
11.1 Randomisation 58
11.2 Outcome measures 58
11.2.1 Phase 2 - Primary outcome measure 58
11.2.2 Secondary outcome measures 58
11.3 Sample size calculation 58
11.4 Statistical analyses 59
11.5 Subgroup analyses 59
12.0 FAKTION Translational research 60
12.1 Pharmacokinetic analysis of Fulvestrant 60
12.2 Samples for mutational analysis and future translational research 60
13.0 Publication policy 61
14.0 Informed consent, ethical and regulatory considerations 61
14.1 Ethical and other issues 61
14.2 Regulatory status 62
14.3 Regulatory considerations 62
14.4 Research Governance approval 62
14.5 Sponsorship 62
14.6 Indemnity 63
14.7 Data protection 63
14.8 Finance 63
15.0 References 64

Appendix 1: RECIST criteria 68
Appendix 2 – Change in Tumour Size 77
Appendix 3: List of concomitant medications that should be avoided 79
Appendix 4: Management of toxicities 85
Appendix 5: Hy's Law 92
Appendix 6: A guide to performing causality assessments 95

Abbreviations and glossary

**ABC**	Advanced Breast Cancer<br>Includes locally advanced disease not amenable to surgical resection and metastatic (stage 4) disease
**AE**	Adverse Event
**AI**	Aromatase Inhibitor
**Akt**	Also known as Protein Kinase B (PKB), a serine/threonine-specific protein kinase
**AKT**	The gene which codes for Akt
**BC**	Breast Cancer
**cfDNA**	Circulating free Deoxy-ribose Nucleic Acid
**CI**	Chief Investigator
**CR**	Complete Response
**CRF**	Case report form
**CR-UK**	Cancer Research UK
**CT**	Computerised axial tomography
**CTA**	Clinical Trial Authorisation
**CTCAE**	Common Terminology Criteria for Adverse Events
**DoR**	Duration of Response
**DLT**	Dose Limiting Toxicity
**ER**	Estrogen Receptor
**ER+**	Estrogen Receptor Positive
**EudraCT**	European Union Drug Regulatory Agency Clinical Trial
**GCP**	Good Clinical Practice
**GP**	General practitioner
**HL**	Hy's Law
**IB**	Investigator's brochure
**ICH**	International Conference on Harmonisation
**ICH-GCP**	International Conference on Harmonisation – Good Clinical Practice
**IDMC**	Independent Data Monitoring Committee
**IHC**	Immunohistochemistry
**IMP**	Investigational medicinal product
**ISF**	Investigator site file
**MBC**	Metastatic Breast Cancer
**MHRA**	Medicines and Healthcare products Regulatory Agency
**MREC**	Multi-centre research ethics committee
**MRI**	Magnetic Resonance Imaging
**MTD**	Maximum tolerated dose
**NCRI**	National Cancer Research Institute
**NCRN**	National Cancer Research Network
**NHS**	National Health Service
**NTL**	Non Target Lesion
**ORR**	Objective Response Rate
**OS**	Overall Survival
**Participant**	An individual who has given written informed consent and is participating in trial related activities

**Patient**	A patient under care who may be eligible for the trial but has not yet consented to participate in any trial related activities.
**Pathway Activated**	Tumours with activating mutations in PIK3CA or low/absent expression of PTEN by IHC
**PD**	Progressive Disease
**PFS**	Progression free survival
**PHL**	Potential Hy's Law
**PI**	Principal Investigator
**PI3K**	Phosphatidylinositide 3-kinases
**PIK3CA**	Gene encoding catalytic subunit of class 1 PI3K
**PIS**	Participant information sheet
**PKB**	Protein Kinase B; also known as Akt, a serine/threonine-specific protein kinase
**PR**	Partial Response
**PTEN**	Phosphatase and Tensin Homolog
**PT**	Prothrombin Time
**q28**	28 day cycle
**R&D**	Research and development
**REC**	Research ethics committee
**SAE**	Serious adverse event
**SAR**	Serious adverse reaction
**SD**	Stable Disease
**SOP**	Standard operating procedure
**SRC**	Safety Review Committee
**SUSAR**	Suspected unexpected serious adverse reaction
**TL**	Target Lesion
**TMF**	Trial master file
**TMG**	Trial management group
**TSA**	Tumour Size Analysis
**TSC**	Trial steering committee
**TSF**	Trial site file
**WCTU**	Wales Cancer Trials Unit
**WT or Wildtype**	Tumours without activating mutations in PIK3CA or low/absent expression of PTEN by IHC

1.0 Trial schema

Phase 2: Randomised Trial

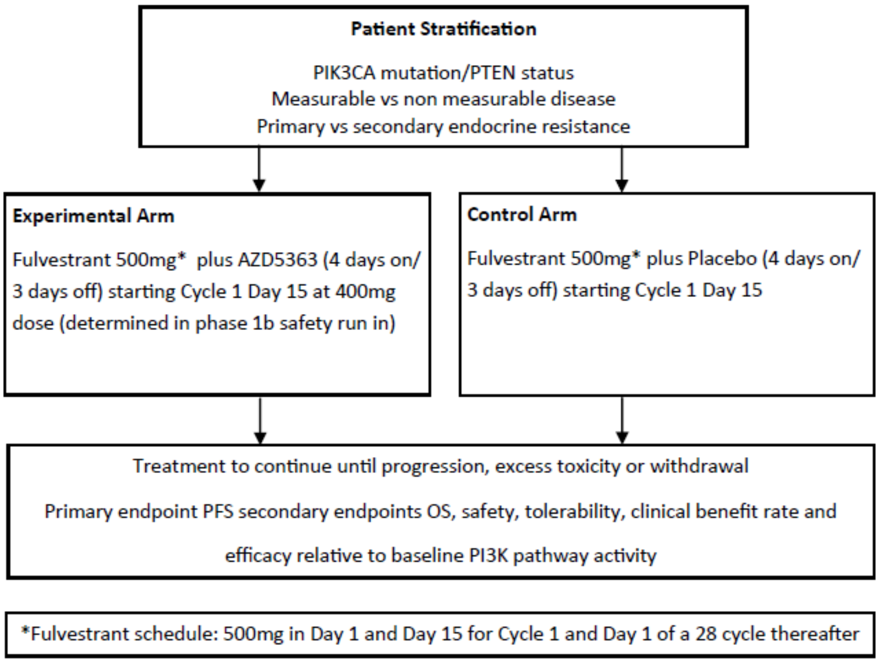

2.0 Trial synopsis

**Study title:**	A phase 1b/2 randomised placebo controlled trial of fulvestrant +/- AZD5363 in postmenopausal women with advanced breast cancer previously treated with a third generation aromatase inhibitor.				
**Study acronym:**	FAKTION				
**Short title:**	**F**ulvestrant +/- **Akt** inhibiti**on** in advanced aromatase inhibitor resistant breast cancer				
**EudraCT No:**	2013-000898-68				
**ClinicalTrials.gov:**	NCT01992952				
**Funder:**	[redacted]	[redacted]	[redacted]		
[redacted] **Investigators:**	[redacted]				
[redacted]	[redacted]	[redacted]	[redacted]		
**Study period:**	5 years	**Phase:**	1b/2	**Number of arms:**	2
**Number of participants:**	Up to 150 in total, the number of participants in phase 1b was 9. The target sample size in phase 2 is 138. If after the TSA of 40 wildtype participants PIK3CA mutation selection is required recruitment will continue until 98 PIK3CA mutant patients is achieved.				
**Investigational Medicinal Products(s)**	The IMP for this trial is AZD5363 a selective inhibitor of the kinase activity of the				

**(IMP)**	serine/threonine Akt/PKB (protein kinase B).

Study design

Stage 1: Phase 1b safety run in. [redacted]
Stage 2-4: Randomised double blind phase 2 expansion:

Stage 2: Inclusion of participants irrespective of PI3K/Akt/PTEN pathway activation.

Stage 3: After 40 wildtype (WT) participants are recruited, followed-up and their data are collected a pre-planned interim analysis will be performed. Inclusion of participants irrespective of PI3K/Akt/PTEN pathway activation continues during this stage.

Stage 4: Following the interim analysis, if activity of AZD5363 is confirmed in participants without pathway activation, inclusion of participants irrespective of PI3K/Akt/PTEN pathway activation will continue up to 138 participants. If not, trial entry will be restricted to participants with non-wildtype tumours until 98 PIK3CA mutant patients are recruited.

Primary Objectives

Phase 1. Safety run-in:

- To assess the safety and tolerability of AZD5363 when combined with fulvestrant in participants with advanced breast cancer (ABC); and to recommend, by assessment of dose limiting toxicities and other safety and tolerability data, a dose of AZD5363 for further study in combination with fulvestrant

Phase 2. Randomised expansion:

- To assess the relative anti-tumour activity of AZD5363 in combination with fulvestrant vs. fulvestrant plus placebo in terms of progression free survival (using RECIST 1.1, Appendix 1) in women with ER+ ABC

Secondary objectives (summarised)

Phase 1. Safety run-in:

- To make a preliminary assessment of the anti-tumour activity of AZD5363 in combination with fulvestrant

Phase 2. Randomised expansion:

- To assess the safety and tolerability of AZD5363 in combination with fulvestrant compared with fulvestrant plus placebo

- To examine the relative efficacy of fulvestrant plus AZD5363 vs fulvestrant plus placebo in sub-populations of patients with and without activation of the tumour PI3K/Akt/PTEN pathway

Phase 1 and 2:

- To assess the impact of AZD5363 on the pharmacokinetics of fulvestrant

- To store genetic samples for future translational research aimed at the identification of predictive

markers for response to Akt inhibition with AZD5363

Main inclusion criteria:

- Post-menopausal women
- Minimum life expectancy of 12 weeks
- Histological confirmation of ER+ve, breast cancer
- Provision of pathological block for molecular analysis of PI3K/Akt/PTEN pathway activation status. If insufficient tumour available contact WCTU for further information.
- Clinical or histological confirmation of metastatic or locally advanced disease not amenable to surgical resection (defined as Advanced Breast Cancer; ABC)
- ECOG performance status 0 to 2
- Measurable or non-measurable disease
- Adequate bone marrow, renal and hepatic function
- Progressive disease whilst receiving an aromatase inhibitor (AI) for ABC, although this does not need to be the most recent therapy, OR relapsed with ABC whilst receiving an AI in the adjuvant setting
- Up to 3 prior lines of endocrine therapy for ABC
- Up to 1 line of chemotherapy for ABC – Note: a chemotherapy regimen that was discontinued due to toxicity, during, or within 6 weeks of the first dose, with a maximum of one cycle delivered and no evidence of disease progression clinically or radiologically at the time subsequent therapy was initiated, is not considered a line of therapy.
- Suitable for further endocrine therapy
- Informed consent

Additional inclusion criteria during Phase 2 (Stage 4):

- If, following pre-planned interim analysis, the activity of AZD5363 is not confirmed in wildtype tumours, recruitment will be restricted to participants with tumours harbouring PIK3CA mutations only.

Main exclusion criteria:

- Previous treatment with fulvestrant or PI3K/mTOR/Akt inhibitor therapy
- Treatment with chemotherapy, immunotherapy or targeted, biologic or tumour embolisation within 21 days of study drug administration
- Palliative radiotherapy within 7 days of study drug
- Clinically significant abnormalities in glucose metabolism including diabetes mellitus
- Rapidly progressive visceral disease not suitable for further endocrine therapy
- Spinal cord compression or brain/leptomeningeal metastases that have not been controlled with surgery or radiotherapy
- Any co-existing medical condition precluding trial entry Including significant cardiac disease (to be defined in protocol)
- Concomitant medication unsuitable for combination with trial medication including certain commonly used antiemetics and statins (see Appendix 3)

Treatments:

Phase 1b (n=9) - [redacted]

All participants were treated in 28 day cycles until disease progression or discontinuation of treatment due to toxicities or patient withdrawal. Fulvestrant 500mg, was given in two 250mg slow (1-2 minute) intramuscular injections on day 1 and day 15 in Cycle 1, then 500mg on day 1 only of subsequent cycles.

AZD5363 was given orally twice daily, starting on day 15, on an intermittent schedule (4 days on/ 3 days off). Starting dose of AZD5363 was 400mg and dose limiting toxicities (DLTs) were assessed by the safety review committee after one cycle of treatment. The SRC reviewed the dosing, toxicity and clinical outcome data of all 9 evaluable and non-evaluable patients recruited to phase 1b and the unanimous recommendation was that the phase 1b established tolerated dose for AZD5363 was 400mg and this is the dose being taken forward into phase 2.

Phase 2 (n=138)

Experimental arm: Fulvestrant (scheduled as above) plus AZD5363 starting on day 15, at 400mg dose (defined from phase 1b) given po bd (4 days on/ 3 days off).
Control arm: Fulvestrant (as above) plus placebo, starting on day 15, given po bd (4 days on/ 3 days off).

Trial assessments after consent taken:

Baseline (inclusive of eligibility screening collected after participant consent):

- Physical examination
- WHO performance status
- Clinical disease assessment
- Baseline toxicity assessment
- Baseline cardiac function assessment
- Radiological disease assessment
- Collection of screening blood samples
- Collection of archival tissue sample

During treatment –until disease progression, up to week 104 (cycle 26)

Participants will be reviewed by a clinician on Cycle 1, Days 1 and 15 and on weeks 4, 8, 16, 24 and then every 12 weeks. A trials nurse will review the participant at each fulvestrant administration. CT scans will be performed at 8, 16 and 24 weeks after registration and at 12 weekly intervals thereafter. Participants will be treated until disease progression (or unacceptable toxicity or withdrawal of consent). The following assessments will be made:

- Physical examination
- ECOG performance status
- Treatment details
- Toxicity assessment
- Radiological disease assessment (weeks 8, 16, 24, 36, 48, 60; and every 12 weeks thereafter)
- Clinical disease assessment (at weeks 72, 84 and 96 if radiological disease assessment not done)

If participants have not progressed by week 104 they can remain on trial therapy. Assessment of disease will be according to local PIs' practice and trial based monitoring will change to a three monthly review, collecting data on date of progression, SAEs, trial withdrawal or death only. Trial assessments will continue until study closure.

Endpoints:

Primary outcome measure:

- Progression-free survival

Secondary outcome measures:

- Safety, tolerability and feasibility of use
- Objective response rate and clinical benefit as assessed by RECIST 1.1
- Overall survival, time from randomisation to death with those still alive censored at date last seen
- The influence of mutational status of PIK3CA and the level of expression of PTEN on outcome in the two treatment groups
- The influence of AZD5363 on fulvestrant pharmacokinetics
- Exploratory biomarkers

2.1 Lay summary

Breast cancer cells which have the estrogen receptor (ER positive or ER+) are more likely to grow when estrogen is present in the bloodstream. This can be treated by drugs that interfere with the action of estrogen or the estrogen receptor. Examples of this include Tamoxifen, and the Aromatase Inhibitors (Anastrozole Letrozole and Exemestane). Although these drugs are often effective for a while, the cancer can frequently become resistant and the drugs stop working. Patients then require treatment with chemotherapy. In this trial, we are investigating whether we can reverse resistance to hormone therapy by adding an additional oral drug called AZD5363. This is a targeted therapy that blocks the action of a cellular protein called Akt, which has been shown to cause resistance to hormone therapy. We will combine this drug with fulvestrant, another hormone therapy which is sometimes used alone in patients who have developed resistance to Aromatase Inhibitors, or Tamoxifen. Thus, patients entering the trial will have a known drug with proven efficacy and will also possibly receive the experimental drug which may enhance activity.

The study is divided into two phases. The first phase is now closed to recruitment; it involved a small number of patients to test if fulvestrant and AZD5363 could be combined in a safe manner (safety run in phase 1b). All patients received a standard dose of fulvestrant and the phase 1b was used to determine the dose of AZD5363 used in phase 2. The dose of AZD5363 determined to be safe for use in phase 2 was 400mg. This is a randomised controlled phase 2 trial where half the patients will receive fulvestrant alone (plus a dummy drug - placebo) and half will receive a combination of the fulvestrant and AZD5363 at 400mg. This will involve a larger number of patients and will allow us to compare the anticancer activity of fulvestrant alone versus fulvestrant and AZD5363.

During the trial, we will monitor patients to ensure the treatment is both safe and effective. If a patient needs to come off treatment (either because of side effects or because the treatment isn't working) they will be offered alternative treatment options outside of the trial. The main method to measure treatment success will be a computerised axial tomography (CT) scan to determine how long the treatment controls the cancer after starting it (Progression Free Survival) but we will also look at how long patients live for (Overall Survival). The trial will have specific eligibility criteria to select patients who we believe can be treated safely and have a good chance of receiving benefit from the treatment. This will be reflected in the screening process.

We will also investigate whether DNA mutations in the original cancer specimen or on cancer cells in the blood determine the likelihood of treatment success. This will be possible by examining the original cancer specimen from a previous biopsy or operation, and a blood test when entering the trial.

3.0 Background

3.1 Investigational agent

AZD5363 is a potent, selective inhibitor of the kinase activity of the serine/threonine Akt/PKB (protein kinase B) that is being developed as a potential treatment for solid and haematological malignancies.

Akt is part of the AGC family of kinases. Mammalian cells express three closely related Akt isoforms: Akt1 (PKBα), Akt2 (PKBβ) and Akt3 (PKBγ), all encoded by different genes. Akt is a node of multiple signalling pathways promoting tumorigenesis, inhibiting apoptosis, impacting on cell cycle and promoting invasion and migration.

The Phosphoinositide 3-kinase (PI3K)/ Akt/ Phosphatase and Tensin Homolog (PTEN) pathway is frequently deregulated in cancer and drives tumour growth and cell survival (Lindsley 2010). All three Akt isoforms are activated in different tumour types including breast, prostate, ovarian, pancreatic and gastric cancers, and this activation is often associated with resistance to established cancer therapies as well as advanced disease and/or poor prognosis (Altomere and Testa 2005). Akt activation in tumours is largely due to input from other signalling pathways upstream of Akt (e.g., mutation of oncogenes such as Ras, Bcr-abl, mutation of receptor tyrosine kinases such as EGFR, amplification of Her2, loss of PTEN function, mutations of PI3K).

Inhibitors of Akt are anticipated to have efficacy when combined with cytotoxic chemotherapies or when combined with targeted or antihormonal agents. AZD5363 inhibits all three Akt isoforms and therefore has the potential to provide clinical benefit over a range of therapeutic indications.

3.2 Non-clinical information and correlative studies

AZD5363 is a potent inhibitor of Akt 1, 2 and 3 in enzyme assays and inhibits the phosphorylation of Akt substrates in cells. AZD5363 inhibits the proliferation of a range of cell lines derived from solid and haematological tumours. Breast cancer cell lines appear to be the tumour types that show the greatest sensitivity to AZD5363. AZD5363 shows dose dependent pharmacodynamic and antitumour activity in xenografts at well-tolerated doses, and can enhance the efficacy of existing treatment (fulvestrant, trastuzumab and docetaxel) in appropriate xenograft models.

Studies in vitro show AZD5363 to be a potent inhibitor of Akt 1, 2 and 3 (concentration giving 50% of the drug-induced inhibitory effect [IC50] <10 nM), and to inhibit protein kinase A (PKA) with a similar potency. AZD5363 inhibits Rho associated protein kinase (ROCK) 1 and ROCK2 with moderate potency (IC50: 126 nM and 56 nM, respectively). In counter screens against ~200 kinases, AZD5363 showed activity against 13 kinases other than AKT1, 2 and 3, with >75% inhibition at 1 µM.

In cell lines, AZD5363 inhibits the phosphorylation of substrates of glycogen synthase kinase 3 β (GSK3β) and proline rich Akt substrate of 40 kilodaltons (PRAS40) with a potency of <1 µM. AZD5363 also inhibits the kinase activity of PKA with a potency of approximately 1 µM in a tumour cell line, but displays low activity for phosphorylation of cofilin, which lies downstream of ROCK.

AZD5363 inhibits the proliferation of 22 tumour cell lines with a concentration causing 50% inhibition of cell growth (GI50) of <1 µM, including the 2 tumour cell lines in which inhibition of Akt substrates was

demonstrated. Breast cancer cell lines appear to show the greatest sensitivity to AZD5363 as monotherapy *in vitro*.

[redacted]

[redacted]

[redacted]

Preclinical data have also been published for inhibitors of PI3K and mTOR demonstrating differential sensitivity by PIK3CA mutation status. For example, the pan-PI3K inhibitors pictilisib (GDC-0941; O'Brien et al 2010) and buparlisib (BKM120; Sanchez et al 2011), the mTOR inhibitor everolimus (Di Nicolantonio et al 2010) and the dual PI3K/mTOR inhibitor BEZ235 (Serra et al 2008) were all shown to preferentially inhibit cell lines with PIK3CA mutations. However, the preclinical data have not been borne out clinically with only one of 8 clinical studies reporting significant preferential sensitivity in tumours bearing PIK3CA mutations (Krop et al 2016; Schmid et al 2016; Hortobagyi et al 2016; Moynahan et al 2016; Blackwell et al 2015; Mayer et al 2014; Balko et al 2015; Ma et al 2015; Tamura et al 2016; Baselga et al 2015).

3.3 Clinical information

Currently 5 AZ-sponsored Phase 1 and Phase 2 studies have been conducted or are ongoing, and 18 investigator-sponsored studies are planned or are ongoing (of which several have completed recruitment).

AZD5363 continuous twice daily (bd) dosing: 80 mg, 160 mg, 240 mg, 320 mg, 400 mg, 480 mg, and 600 mg.

AZD5363 intermittent bd dosing (4 days on treatment followed by 3 days off treatment): 480 mg, 400 mg, and 320mg.

AZD5363 intermittent bd dosing (2 days on treatment followed by 5 days off treatment): 640 mg and 800 mg.

The most commonly reported adverse events (AEs), regardless of dose, schedule or causality, are diarrhoea, nausea, hyperglycaemia, fatigue, vomiting, decreased appetite, and rash (maculo-papular). The majority of AEs reported in patients receiving AZD5363 have been CTCAE Grade 1 or Grade 2 and clinically manageable.

[redacted]

[redacted]

[redacted]

AZD5363 in advanced or metastatic breast cancer
The first-in-human evaluation of AZD5363 was conducted in Study D3610C00001, a multipart, Phase 1, open-label, multicentre study in patients with advanced solid malignancies with the aim at investigating the safety, tolerability, and pharmacokinetics of AZD5363 as well as at defining recommended dosing schedule.

In Part A –dose escalation – and in Part B – dose expansion (Banerjii et al 2017), a total of 90 patients, who were not prospectively characterised according to tumour mutation status, received AZD5363 in either the continuous or intermittent (4 days on, 3 days off; or 2 days on, 5 days off) schedule.
The MTD for the continuous schedule was 320 mg bd, while the lower dose considered tolerable for the intermittent schedule was 480 mg bd (4 days on, 3 days off) and 640 mg bd (2 days on, 5 days off).
The most frequently reported AEs across all dosing schedules were diarrhoea, vomiting, and nausea and, for CTCAE grade >3 events, hyperglycaemia.

The expansion Parts C and D of the study were restricted more specifically to patients with solid tumours harbouring PIK3CA (Part C) or AKT 1 mutations (Part D).

The expansion parts of Study D3610C00001 (Parts C and D) recruited an advanced cancer population (ER+ breast cancer, gynaecological cancer, or other solid tumour types) selected for patients with proven *PIK3CA* (Part C) or *AKT1* (Part D) mutations (Banerji et al 2017; Hyman et al 2017). In patients with ER+ breast cancer, 1/28 patients with measurable disease had a confirmed RECIST PR in Part C (*PIK3CA*

mutation). In patients with gynaecological cancer, 2/26 patients with *PIK3CA*-mutant cancer and measurable disease had a confirmed RECIST PR. In *AKT1*-E17K-mutant patients, confirmed PRs were observed in ER breast (4/20 patients) and gynaecological cancers (4/18 patients, including cervical cancer [n=1], endometrial cancer [n=2], and ovarian cancer [n=1]), as well as 2/20 patients with other cancers including triple-negative breast and lung adenocarcinoma (n=1 each). Median PFS in the *AKT1*-E17K-mutant ER+ breast, gynaecologic, and other solid cancer cohorts was 5.4 months (95% CI 2.9 to 6.8 months), 5.8 months (95% CI, 2.6 to 8.2 months), and 2.6 months (95% CI, 1.4 to 5.6 months), respectively.

Parts E and F expansion cohorts of Study D3610C00001 are designed to explore the combination of AZD5363 400 mg BD, 4 days on 3 days off, in combination with fulvestrant 500 mg in ER+/HER2- metastatic breast cancer patients: 1) AKT1 mutant, enrolled into fulvestrant-naïve (Edelay or Ed) and fulvestrant-resistant (Ereverse or Er) cohorts; 2) PTEN mutant, enrolled into fulvestrant-naïve (Fdelay or Fd) and fulvestrant-resistant (Freverse or Fr) cohorts. The planned interim analyses of Part E conducted when 12 patients/cohort reached maturity for assessment of 24-week clinical benefit rate showed that AZD5363 plus fulvestrant is clinically active in patients with AKT1m ER+ metastatic breast cancer, including those with prior resistance to fulvestrant (Smyth et al 2017). The clinical benefit rate was 42% and 33% in the Er and Ed cohorts, respectively.

A further study investigating safety and tolerability of AZD5363 in patient with advanced or metastatic breast cancer is D3610C00002 (BEECH), a Phase 1/2, multicentre study comprising a safety run-in with AZD5363 combined with paclitaxel (Part A); followed by a randomised expansion of paclitaxel combined with AZD5363 or placebo in a 1:1 randomisation schedule (Part B).

The study was designed to identify a recommended dose and schedule of AZD5363 in combination with weekly paclitaxel and to investigate whether AZD5363, when combined with paclitaxel, had the potential to improve PFS in patients with ER+HER2- advanced or metastatic breast cancer, stratified by PIK3CA mutation status.

In Part A of the study, the dosing schedule of 400mg bd 4 days on, 3 days off 400mg bd was selected as the recommended dose for Part B of the study.

The most commonly reported AEs in those treated at 400mg bd (4 days on/3 days off) were diarrhoea, alopecia, nausea, and anaemia.

Primary analysis of D3610C00002 (BEECH) has been reported (Turner et al 2017). Adding AZD5363 to weekly paclitaxel did not significantly prolong progression-free survival in the overall population or in the PIK3CA mutation positive subgroup of ER+/human epidermal growth factor receptor (HER)-negative (HER) advanced or metastatic breast cancer patients.

3.4 Pharmacokinetics

Pharmacokinetic exposure increased dose proportionally in a dog model but more than dose proportionally in a rat model. Minimal accumulation was seen after multiple daily dosing in both the rat and dog. AZD5363 free fraction in human serum albumin and human α1-acid glycoprotein were 29.5% and 66.5-75.8%, respectively. In vitro the major human metabolite was a direct glucuronide conjugate (via UGT1A9 and UGT2B7). CYP3A4 was mainly responsible for the formation of monooxygenated metabolites, with contributions from CYP2C9 and CYP3A5. AZD5363 produced reversible inhibition of CYP2D6, CYP3A4/5, CYP2C9, CYP2B6 and CYP2C19. Time-dependent inhibition of CYP3A4/5 was observed. In a rat quantitative whole-body autoradiography (QWBA) study there was persistence of radioactivity up to at least 168 and 504 hours after dosing in pigmented skin and the uveal tract of the eye respectively.

AZD5363 has been found in an *in vitro* assay to inhibit Organic Cation Transporter 2 (OCT2), found in the human kidney. In a clinical setting this has the potential to increase a patient's serum creatinine level, and also to increase the plasma levels of drugs known to be excreted by this transporter, including metformin. Metformin is a treatment option for the management of hyperglycaemia occurring in patients participating in studies of AZD5363.

Findings from previously described Phase I studies were that the AZD5363 time to maximum concentration (tmax) occurred at approximately 2 hours following either single or multiple doses. The apparent terminal half-life following single doses ranged from 8 to 15 hours. The accumulation ratio (RAC) values were consistent with the measured half-life and the linearity factor values were generally close to unity, indicating that the multiple dose kinetics were consistent with the single dose data. The exposure of AZD5363 increased approximately proportionally with AZD5363 dose for the dose range 80 mg to 400 mg.

Further details are provided in the Investigator's Brochure.

3.5 Research hypotheses

Phase 1. Safety run-in: AZD5363 when combined with fulvestrant will have an acceptable safety and tolerability profile. No pharmacokinetic interaction between the drugs is expected.

Phase 2. Randomised expansion:

The addition of AZD5363 to fulvestrant will be more efficacious than fulvestrant alone - as demonstrated by an increase in progression-free survival in patients with Estrogen-Receptor positive (ER+ve) advanced or metastatic breast cancer.

Breast cancers that have mutations in PIK3CA or low/reduced PTEN expression by IHC will have increased sensitivity to AZD5363 and, therefore, demonstrate greater improvement in PFS to AZD5363 when combined with fulvestrant than those without such molecular aberrations.

3.6 Rationale for conducting this study

3.6.1 Estrogen Receptor Positive Metastatic Breast Cancer - Background

In 2010, 49,564 women in the UK were diagnosed with invasive breast cancer and 11,556 died (http://www.cancerresearchuk.org). The majority of early breast cancers are ER+ and women with such tumours receive adjuvant endocrine therapy. Such endocrine therapy will cure approximately 30% of women with undetected micro-metastatic disease but 70% will relapse and subsequently die from ER+ endocrine resistant metastatic breast cancer (MBC) (Davies et al 2011). In ER+ ABC, endocrine therapy is the treatment of choice due to its improved toxicity profile and comparable efficacy when compared with cytotoxic chemotherapy. However, half of such cancers will progress through first line therapy (primary endocrine resistance) and half will progress after an initial period of disease control (secondary or acquired endocrine resistance). There is a significant need to improve upon current endocrine therapies by circumventing these resistance mechanisms.

3.6.2 Rationale for using fulvestrant as the endocrine backbone

Aromatase inhibitors (AIs) have, until recently, been the treatment of choice for de novo metastatic disease as well as for patients treated with adjuvant tamoxifen (Mauri et al 2006). Increasingly, however, AIs are also being used in the adjuvant setting and the most efficacious sequence of endocrine therapies in this situation has not been defined. Fulvestrant at a dose of 250mg q28 days was shown to be as effective as the steroidal AI exemestane following failure of a non-steroidal AI (anastrazole or letrozole) in postmenopausal women with metastatic breast cancer (Chia et al. 2008). Subsequently fulvestrant at a dose of 500mg q28 days with an extra loading dose on day 15 was shown to be superior to the 250mg dose given according to the same schedule and is currently licenced at the higher dose (Di Leo et al, 2010).

The use of third-generation aromatase inhibitors in endocrine-naive advanced disease has recently been challenged by two randomised studies. In the FIRST study, fulvestrant at the 500mg dose demonstrated superior efficacy compared with anastrozole (Robertson et al, 2012). Fulvestrant treatment resulted in superior progression-free survival (PFS) versus anastoazole (23.4 months versus 13.1 months, HR 0.66; 95% CI 0.47–0.92, P<0.01) despite a lack of improvement in clinical benefit rate [72.5 versus 67.0%, respectively (odds ratio, 1.30; 95% CI, 0.72–2.38; P=0.386)]. In the phase 3 FALCON trial 462 postmenopausal women were randomised 1:1 to fulvestrant or anastrozole (Robertson et al, 2016). Progression-free survival was significantly longer with fulvestrant (HR 0·797, 95% CI 0·637-0·999, p=0·0486). Median progression-free survival was 16·6 months (95% CI 13·83-20·99) in the fulvestrant group versus 13·8 months (11·99-16·59) in the anastrozole group.

3.6.3 Rationale for using AZD5363 in advanced endocrine resistant ER+BC

In many experimental models of acquired endocrine resistance, increased Akt signalling is evident, reactivating ER and providing strong proliferation/survival signals in an ER dependent and ER independent manner (Miller et al, 2011; Saal et al, 2005; Stemke-Hale et al 2008). Increased Akt activity has also been associated with clinical endocrine resistant states (Miller et al 2011; Sabnis et al 2007). Ligand independent ER activity driven by CDK4/E2F mediates survival in long term estrogen deprived (LTED) cells and fulvestrant antagonises this activity and synergises with PI3K/Akt inhibition to markedly increase apoptosis (Miller et al, 2011). In MCF-7aro LTED xenografts (ER persistent) PI3K/Akt pathway inhibitor plus fulvestrant was the most efficacious growth inhibitory combination tested (Sabnis et al 2007). Taken together, these data provide a strong rationale for testing Akt inhibitors clinically in combination with fulvestrant after aromatase inhibitor treatment.

Multiple potential mechanisms of endocrine resistance have been defined pre-clinically (Osborne and Schiff 2011). Persistent ER can act as a focus for crosstalk with multiple intracellular signalling pathways such as PI3K/Akt, even in the absence of estrogen (Miller et al 2011). This pathway is activated in approximately 50% of ER+ breast cancer due to mutations in PIK3CA or loss of PTEN protein expression (Saal et al 2005; Stemke-Hale et al 2008; Ellis et al 2012). Adaptive upregulation of the PI3K/Akt pathway is also a feature of preclinical models of aromatase inhibitor resistance (Sheri et al 2010). The clinical importance of this pathway in endocrine resistance has recently been demonstrated with the mTOR inhibitor everolimus in combination with exemestane or tamoxifen, resulting in significant improvements in progression-free survival over endocrine therapy alone in randomised studies (Bachelot et al 2012; Baselga et al 2011). However, ligand independent activation of the ER is not surmounted by agents such as exemestane in contrast to fulvestrant which induces ER degradation.

Thus the combined inhibition ER with fulvestrant and the Akt pathway with AZD5363 may prove an effective strategy in patients with MBC who experience progression or recurrence on or shortly after AI therapy. This trial will evaluate whether Akt inhibition improves the efficacy of fulvestrant in this patient group.

Study Objectives

- To establish whether the combination of AZD5363 and fulvestrant will improve clinical outcome in patients with endocrine resistant advanced breast cancer (RECIST v1.1 Appendix 1)
- To determine whether the combination of AZD5363 and fulvestrant is tolerable, safe and feasible to deliver
- To determine whether AZD5363 activity is related to the activation status of the tumour PI3K/PTEN pathway

4.0 Study design

This is a four stage study, with an initial dose escalation phase 1b study (stage 1) which is now closed and subsequent double blind randomised phase 2 controlled trial (stages 2-4). The phase 2 study is divided into three stages as pre-clinical data support the concept that AZD5363 may be more active in tumours with PI3K/Akt/PTEN pathway activation. The trial is designed to test this concept in the clinical setting, with a pre-planned interim analysis. The four stages are described below.

Eligible patients are post-menopausal women with locally advanced or metastatic ER+ breast cancer not suitable for surgical resection. Patients should be suitable for endocrine treatment, but have received no more than three previous lines of endocrine treatment and up to one line of chemotherapy for ABC. They will also have had progressive disease during treatment with a third generation aromatase inhibitor or have relapsed on an AI in the adjuvant setting. Participants will receive fulvestrant in combination with either placebo or AZD5363 until disease progression. Each cycle will be 28 days in duration. The dose of AZD5363 in the phase 2 trial has been determined by results from the initial phase 1. In both parts of the study fulvestrant is initiated on Cycle 1 D1, whereas AZD5363 (or placebo) is initiated on Cycle 1 Day 15 (see below).

The phase 2 study will use four variables to balance patient allocation, using minimisation with a random element:

- PI3K pathway activation status (assessed by PIK3CA mutation analysis on cfDNA and tumour tissue and PTEN level by IHC on tumour tissue see section 6.4)
    - PIK3CA mutational status (wildtype (WT) or mutated)
    - PTEN level (0/1+ in <10% of tumour cells vs >1+ or ≥10% tumour cells)
- Resistance to prior AI therapy (primary or secondary resistance)
    - Primary resistance is defined as either 1) disease relapse during or within 6 months (i.e. 26 weeks) of completing AI treatment in the adjuvant setting, or 2) disease progression within 6 months of starting AI treatment and no response to AI treatment in the metastatic setting
    - Secondary resistance is defined as 1) disease relapse more than 6 months (i.e. 26 weeks) after completion of AI treatment in the adjuvant setting, or 2) disease progression following achievement of clinical benefit with AI therapy to treat MBC
- Measurable disease (vs. non measurable)

PI3K/Akt/PTEN pathway activation status will be determined by genetic and IHC analysis of the cfDNA and historically obtained tumour tissue (see section 6.4); analysis will occur within 10 working days from receiving the tumour material and blood or plasma samples. In order to minimise treatment delay, participants will initiate fulvestrant on Cycle 1 Day 1, and then be randomised prior to Cycle 1 Day 15, at which point they will initiate AZD5363 or placebo. Results of the cfDNA and tumour tissue analysis should be reported prior to randomisation.

Stage 1: A non-randomised phase 1b dose escalation study (not included in this version of the protocol)

Stages 2-4: A double-blind, stratified and randomised evaluation of AZD5363 when combined with fulvestrant vs. fulvestrant plus placebo. The dose and schedule of AZD5363 has been selected as an outcome from the Phase 1 safety run-in. Stages 2-4 differ only in the eligibility of patients, specifically pertaining to PI3K pathway activation.

Stage 2: An initial group of participants will be included regardless of PI3K/Akt/PTEN pathway activation status. Prospective analysis of PTEN expression and PIK3CA mutation status will be carried out in archival tumour samples and PIK3CA of cfDNA in blood samples prior to randomisation to facilitate participant stratification. Once 40 participants have been recruited with wildtype PI3K pathways (20 in each treatment arm) the trial will progress to stage 3.

Stage 3: An interim analysis of the 40 participants with wildtype PI3K pathways will be performed using the tumour size analysis (TSA) protocol (see section 4.2.2).Whilst data from stage 2 are analysed for the interim analysis to define whether AZD5363 may be of benefit to participants with wildtype tumours, participants will continue to be recruited regardless of PI3K/Akt/PTEN pathway activation status.

Stage 4: When data from stage 2 have been analysed, eligibility for stage 4 will depend on TSA results. If participants with wildtype tumours do appear to derive benefit from the addition of AZD5363 to fulvestrant then participants will continue to be recruited regardless of PI3K/Akt/PTEN pathway activation status. If no apparent benefit from AZD5363 is seen in participants with wildtype tumours then restricted recruitment of participants with tumours harbouring PIK3CA mutations. . These decisions will be in the remit of the interim data monitoring committee (IDMC).

4.1 Risk assessment

A trial risk assessment has been completed by the WCTU to identify the potential hazards associated with the trial and to assess the likelihood of those hazards occurring and resulting in harm. This risk assessment has been completed in accordance with the MRC/DH/MHRA Joint project guidance document 'Risk-adapted approaches to the management of Clinical Trials of Investigational Medicinal Products' and includes:

- The risk to participant safety in relation to the IMP;
- All other risks related to the design and methods of the trial (including risks to participant safety and rights, as well as data integrity);
- The potential risks have been balanced against the level of risk that a trial participant would be exposed to outside of the trial. This trial has been categorised as a TYPE C, where the level of risk is markedly higher than the risk of standard medical care. A copy of the trial risk assessment may be requested from the WCTU Trial Manager. The trial risk assessment is used to determine the intensity and focus of monitoring activity (see section 10.0).

4.2 Phase 2 (Stages 2-4)

A double-blind, stratified and randomised evaluation of AZD5363 when combined with fulvestrant vs. fulvestrant plus placebo. The dose and schedule of AZD5363 is the recommended Phase 2 dose as established from the Phase 1 safety run-in and has been agreed by the SRC. Participants will be randomised to one of two arms:

Control Arm: fulvestrant + placebo

The control arm will consist of fulvestrant 500mg administered on Day 1 of every 28 day cycle with a loading dose on Cycle 1 Day 15 only. Placebo capsules/tablets will be taken twice daily on a 4 days on / 3 days off schedule starting on Cycle 1 Day 15.

Experimental arm: fulvestrant + AZD5363

The experimental arm will consist of fulvestrant 500mg administered on day 1 of every 28 day cycle with a loading dose on Cycle 1 Day 15. AZD5363 capsules/tablets at the recommended Phase 2 dose of 400mg from Phase 1, will be taken twice daily on a 4 days on / 3 days off schedule starting on Cycle 1 Day 15.

4.2.1 Safety review

Two safety reviews will be performed after 20 and 40 participants have received at least 1 cycle of treatment. The WCTU will request that all toxicity forms and any serious adverse events (SAEs) are submitted promptly following the first cycle. Recruitment will continue as normal during these reviews.

4.2.2 Activity review within stratified PIK3CA groups

Recent clinical data from phase I studies and preclinical data in breast cancer cell lines suggest that activity of AZD5363 will be greater in tumours with mutations in PIK3CA or suppression of PTEN; however, this is yet to be confirmed clinically in ER+ve breast cancer.

A review of treatment activity will be performed after 40 participants with confirmed wildtype tumours (confirmed from the archival tissue block) have been randomised in stage 2 and have reached 8 weeks of follow-up (the first radiological analysis of response). Tumour size analysis will be performed (for detailed analysis plan, see Appendix 2) and if there is <10% difference between placebo and AZD5363 treated wildtype participants, then the eligibility criteria will be restricted to participants with PIK3CA mutant tumours only (see section 4.0 study design).

Recruitment will not stop whilst sufficient time elapses for all participants in stage 2 to reach the 8 week time-point and for the analysis to be performed.

Following the interim analysis, if recruitment is restricted to participants with PIK3CA mutations for the remainder of the trial, the target sample size of the PIK3CA positive group will be 98 participants.

Sites will be informed of the results of the activity review, and whether they should restrict recruitment to only PIK3CA mutant participants, or continue recruitment of wildtype participants. An additional screening PIS and separate Phase 2 PIS (to stages 2 and 3) will be provided if recruitment is restricted in stage 4 of the study.

4.3 Completion of Treatment

Participants in both stages of the trial may continue AZD5363/placebo until disease progression or until intolerable treatment toxicity or consent withdrawal. It is estimated that participants will complete treatment by week 60; however participants may be treated beyond this if they have not yet progressed. In phase 2, if AZD5363/placebo is discontinued due to toxicity, the participant may continue on fulvestrant

alone at the investigator's discretion (see section 7.2). If trial treatment is permanently discontinued, the participant should continue trial follow up where possible (see section 10.3). Once trial follow-up is complete and the patient has ended the study, there will be no further assessments. However, death data will be collected until the end of the trial.

NOTE: if the participant ends treatment for reasons other than disease progression (and has not withdrawn consent to follow-up), sites will be asked to follow up if/when a patient starts new treatment. For participants that do not start new treatment, progression data will be requested when it becomes available until the end of the trial.

5.0 Participating centre selection

This study will be carried out at participating sites within the UK. All sites who are interested in participating in the trial will be required to complete a registration form to confirm that they have adequate resources and experience to conduct the trial.

The following documentation must be completed and received by the WCTU in order for a site to begin recruitment:

- Confirmation of local R&D approval
- Favourable opinion of host care organisation/PI from main ethics committee
- Signed partnership agreement between the host care organisation and Sponsor
- Current Curriculum Vitae and good clinical practice (GCP) training certificate of the Principal Investigator (PI)
- A copy of the most recent approved version of the Participant Information Sheet(s) (PIS(s)) and Consent Form(s) on host care organisation headed paper
- A copy of the most recent approved General Practitioner (GP) letter on host care organisation headed paper
- Completed Delegation Log (signature list and delegation of responsibilities)
- Full contact details for all host care organisation personnel involved, indicating preferred contact
- A set of laboratory normal ranges and laboratory certification/accreditation from the host care organisation laboratory being used for analyses

Once all the documentation has been received at the WCTU, confirmation of centre approval will be sent by the WCTU to the site PI and Fisher Clinical Services who are providing the AZD5363 and fulvestrant.

Case report forms (CRFs) will also be sent to the Clinician, Data Manager or Research Nurse nominated as responsible for the participant.

All documentation must be stored in the Investigator Site File (ISF) at the site and in the Trial Site File (TSF) at the WCTU. The WCTU must be notified of any changes to the trial personnel and their responsibilities during the running of the trial and the respective trial files must contain this up-to-date information.

Occasionally during the trial, amendments may be made to the trial documentation listed above. WCTU will issue the site with the latest version of the documents as soon as they become available. It is the responsibility of the site to ensure that they obtain local R&D approval for the new documents, and that all relevant staff, including pharmacy staff, are working to the current versions once R&D approval has been obtained.

Centre initiation will be by attendance at a national FAKTION launch meeting or by teleconference if attendance of key personnel at the launch meeting is unfeasible.

6.0 Participant eligibility

Any queries about whether a patient is eligible to enter the trial should be discussed with the WCTU before enrolment. Any issues will then be raised with the one of the Chief Investigators (CIs) or one of the clinical Co-Investigators in the CIs' absence. If it is confirmed that the inclusion or exclusion criteria are not met, then no eligibility waivers are permitted.

The site will inform the participant's GP of the participant's enrolment, if the participant gives consent to do so.

It may be possible for participants to be recruited into other clinical trials, but this should be discussed with the CI via the WCTU before this is considered.

Patients are eligible for the trial if all the inclusion criteria (Section 6.1.1) are met and none of the exclusion criteria (Section 6.1.2) apply.

6.1 Informed consent

The patient's written informed consent must be obtained using the FAKTION trial Consent Form, which follows the PIS. Patients should be given ample time to consider participation and to discuss with friends and family if required before being asked to sign the Consent Form. Please note, only when written informed consent has been obtained from the patient and they have been registered/enrolled into the trial can they be considered a trial participant.

Patients may also be asked to consent to NHS Information Centre Flagging (and Scottish and Northern Irish equivalents) so that the date and cause of death can be collected without longer term follow-up.

The patient's consent to participate in the trial should be obtained by the treating doctor after a full explanation has been given of the treatment options, including the conventional and generally accepted methods of treatment. All patients must be informed of the aims of the study, the possible adverse events, the procedures and possible hazards to which they may be exposed. They will be informed of the strict confidentiality of their patient data, but that their medical records may be reviewed for trial purposes by authorised individuals other than their treating physician.

Patient's consent will be sought to notify their GP of their involvement in the trial. A contact number for someone at the site should be given to the patient should they wish to discuss any aspect of the trial. Following this, the investigator should determine that the patient is fully informed of the trial and their participation, is in accordance with the principles of GCP. Patients should always be asked to sign a FAKTION trial consent form. One copy should be given to the participant but the original copy should be kept in the investigator site file and a further copy should be kept with participant's hospital notes.

Participant consent is requested to collect NHS Numbers to utilise NHS data for future research, through Cancer Research UK and the National Cancer Intelligence Network (NCIN).

Participant consent is also requested to store tissue and blood samples for future genetic research.

The right of the participant to refuse to participate in the trial without giving reasons must be respected. After the patient has entered the trial, the investigator must remain free to give alternative treatment to that specified in the protocol, at any stage, if he/she feels it to be in the best interest of the participant. However, the reason for doing so should be recorded and the participant will remain within the trial for the purpose of follow-up and data analysis according to the treatment option to which he/she has been

allocated. Similarly, the participant must remain free to end the protocol treatment at any time without giving reasons and without prejudicing his/her further treatment.

This is a randomised controlled trial and therefore neither the participants nor their physicians will be able to choose the participant's treatment. Treatment will be allocated randomly using a computer-based algorithm. This is to ensure that the groups of participants receiving each of the different treatments are similar.

6.2 Screening procedures

Screening logs should be completed for every patient considered for the trial. Copies of these screening logs should be sent to the WCTU upon request.

During Phase 2, immediately after consent the participant's research nurse and/or doctor should contact WCTU to request the sample numbers assigned to that participant. All screening samples in Phase 2 should be labelled with the appropriate sample number provided by the WCTU and the samples sent to the [redacted] laboratory. These numbers should be used for all screening samples associated with that participant and will be linked to the corresponding participant trial ID after randomisation.

The following procedures should be performed as soon after consent as possible and before the commencement of study treatments:

- Archival tumour tissue to be requested from the local pathology laboratory for molecular analysis of PI3K/Akt/PTEN pathway activation status, if insufficient tumour available contact WCTU for further information.
- Two 10ml blood samples to be collected; the first will be used to test cfDNA for PIK3CA status. The second will be stored for future correlative translational studies.
- An additional 10 ml blood sample may be requested by WCTU; this will be processed at the time of collection, the plasma removed and stored frozen at site until collection is arranged by WCTU (see section 12.2).

The following assessments and procedures should be performed **within 28 days** prior to registration in to the study to confirm eligibility:

- Medical history and physical examination including ECOG performance status, weight, vital signs
- Concomitant medication
- CT of chest, abdomen (± pelvis as clinically indicated) and/or other RECIST v1.1 compatible imaging
- MUGA scan/echocardiogram
- Blood tests
    - FSH and estradiol
    - Fasting glucose
    - Glycosylated haemoglobin
    - Lipids

The following assessments and procedures should be performed **within 7 days** prior to registration to confirm eligibility:

- Electrocardiograph (ECG) x 3 (1 minute apart)
- Bloods tests
  - Full blood count
  - Serum biochemistry
  - Prothrombin time
  - Magnesium
- Urine dipstick for protein and glucose

6.2.1 Inclusion criteria

Patients meeting all of the following criteria may be included in the trial:

1. Provision of informed consent prior to any study specific procedures.
2. Adult female patients, aged ≥ 18 years.
3. Postmenopausal patients. Post-menopausal can be defined as either of the following criteria:
   a. Amenorrhoeic throughout AND after therapy with a third generation AI, without a GnRH analogue (eg goserelin) AND FAKTION screening FSH and estradiol in institutional post-menopausal ranges. If estradiol is in institutional post-menopausal ranges but FSH is not and the patient has been clinically post menopausal for over 5 years, the patient may be considered eligible after discussion with the CI's.

      OR

   b. Treatment of early or metastatic breast cancer with a third generation AI and GnRH analogue, with discontinuation of the GnRH analogue for at least 6 months AND no resumption of menstruation AND FAKTION screening FSH and estradiol in institutional post-menopausal ranges.
   c. Patients who received an AI and GNRH analogue combined and subsequently have bilateral oophorectomy are considered eligible for the study if they meet all other eligibility criteria.
4. Histological confirmation of ER+ve breast cancer on primary tumour at diagnosis or on biopsy of a metastasis. ER is considered positive if ≥10% of tumour cells stain positive for ER (whatever the intensity of staining). If no percentage score is available then a Quick (Allred) Score of ≥4/8 will be considered ER positive.
5. Histological confirmation of HER2 negative breast cancer on primary tumour at diagnosis or on biopsy of a metastasis. HER2 is considered negative by IHC if scored as 0 or 1+ by Herceptest or similar assay. If HER2 is scored 2+ or 2+/3+ by IHC then HER2 gene amplification must be assessed by FISH/CISH/D-DISH and the ratio of HER2 to CEP17 probes must be <2.0.
6. Clinical or histological confirmation of metastatic or locally advanced disease not amenable to curative surgical resection.
7. ECOG performance status 0 to 2 with no deterioration over the previous 2 weeks.
8. Minimum life expectancy of 12 weeks.

9. Measurable or non-measurable disease
10. Progressive disease whilst receiving a third generation aromatase inhibitor (exemestane, anastrazole or letrozole) for locally advanced or metastatic BC or relapsed with metastatic disease whilst receiving a third generation AI in the adjuvant setting. The AI does not need to be the last treatment immediately prior to recruitment.
11. No more than 3 prior lines of endocrine therapy for ABC. If an attempt to downstage a locally advanced tumour with endocrine therapy was made in the absence of MBC, and the tumour operated upon, then this does not count as a line of therapy for ABC. In contrast, if the tumour remained inoperable then this treatment should be included as a line of therapy for ABC.
12. No more than 1 line of cytotoxic chemotherapy for ABC (Adjuvant and neo-adjuvant chemotherapy are not classed as lines of chemotherapy for ABC [see inclusion criterion 11] A chemotherapy regimen used to treat ABC but that was discontinued due to toxicity, during, or within 6 weeks of the first dose, with a maximum of one cycle delivered and no evidence of disease progression clinically or radiologically at the time subsequent therapy was initiated, is not considered a line of therapy).
13. Suitable for further endocrine therapy according to the treating clinician.
14. Radiological or objective clinical evidence of recurrence or progression on or after the last systemic therapy prior to enrolment.
15. Provision of most recent archival tumour sample for PIK3CA mutation and PTEN by IHC testing. If insufficient tumour available contact WCTU for further information.
16. Provision of baseline plasma sample for PIK3CA mutation testing on cfDNA.
17. Patient has adequate bone marrow and organ function as defined by the following:
    a. Absolute Neutrophil Count (ANC) ≥ 1.0 x 10^9/L;
    b. Platelets (plt) ≥ 100 x 10^9/L;
    c. Haemoglobin (Hgb) ≥ 9 g/dl [Note: any blood transfusion must be >14 days prior to the determination of haemoglobin];
    d. PT ≤ 1.5xULN; Treatment with coumarins such as warfarin is not permitted in the study.
    e. Potassium, calcium (corrected for serum albumin) and magnesium within normal limits (WNL) for the institution;
    f. Serum creatinine ≤ 1.5xULN;
    g. Alanine aminotransferase (AST) or aspartate aminotransferase (ALT) ≤ 1.5xULN (or < 3.0 x ULN if liver metastases are present);
    h. Total bilirubin ≤1.5 times ULN.
18. Patients with type II diabetes mellitus that is well controlled by dietary measures alone and have an HgA1c < 8% are eligible to participate.

NB in phase 2 (Stage 3): Additional inclusion criteria may apply during phase II of the trial

After 40 wildtype participants have been recruited into phase 2 and the TSA completed, recruitment may be restricted to participants with tumours harbouring PIK3CA mutations only – please see section 4.2.2 for further details.

6.2.2 Exclusion criteria

If any of the following criteria apply, patients cannot be included in the trial:

1. Previous treatment with fulvestrant or inhibitors of the PI3K/mTOR/Akt pathway to treat breast cancer;
2. Clinically significant abnormalities of glucose metabolism as defined by any of the following:
    a. Diagnosis of diabetes mellitus type I;
    b. Fasting plasma glucose [fasting is defined as no calorific intake for at least 8 hours]: ≥ 7.0mmol/L (126 mg/dL) for those patients without a pre-existing diagnosis of Type 2 diabetes mellitus

       ≥ 9.3 mmol/L (167mg/dL) for those patients with a pre-existing diagnosis of Type 2 diabetes mellitus
    c. Glycosylated haemoglobin (HbA1C) ≥8.0% at screening (64 mmol/mol)

       (conversion equation for HbA1C [IFCC-HbA1C (mmol/mol) = [DCCT-HbA1C(%) – 2.15] x 10.929):
    - Requirement for insulin for routine diabetic management and control
    - Requirement for more than two oral hypoglycaemic medications for routine diabetic management and control
3. Rapidly progressive visceral disease not suitable for further endocrine therapy.
4. Last dose chemotherapy, immunotherapy, targeted therapy, biological therapy or tumour embolisation must be more than 21 days (more than 6 weeks for nitrosurea or mitomycin C) prior to the first dose of study treatment (fulvestrant). *Note: endocrine (hormone) therapy is not considered a targeted or biological therapy for the purposes of this study*. Denosumab and bisphosphonate treatment are accepted concomitant medications as long as they are started at least 14 days prior to study drug commencement.
5. Last dose of palliative radiotherapy must be more than 7 days prior to the first dose of study treatment (fulvestrant).
6. Major surgery (excluding placement of vascular access) within 4 weeks before the first dose of study treatment (fulvestrant).
7. Spinal cord compression or brain metastases unless asymptomatic, treated and stable and not requiring steroids for at least 4 weeks prior to start of study treatment.
8. As judged by the Investigator, any evidence of severe or uncontrolled systemic diseases, including uncontrolled hypertension, active bleeding diatheses, or active infection including hepatitis B, hepatitis C and human immunodeficiency virus (HIV). Screening for chronic conditions is not required.
9. Any of the following cardiac criteria:
    a. Mean resting corrected QT interval (QTc) >470 msec obtained from 3 consecutive ECGs (taken 1 minute apart);
    b. Any clinically important abnormalities in rhythm, conduction or morphology of resting ECG eg, complete left bundle branch block, third degree heart block;
    c. Any factors that increase the risk of QTc prolongation or risk of arrhythmic events such as heart failure, hypokalaemia, potential for torsades de pointes, congenital long QT syndrome, family history of long QT syndrome or unexplained sudden death under 40 years of age or any concomitant medication known to prolong the QT interval;

d. Experience of any of the following procedures or conditions in the preceding 6 months: coronary artery bypass graft, angioplasty, vascular stent, myocardial infarction, angina pectoris, congestive heart failure NYHA Grade 2.
e. Uncontrolled hypotension – SBP <90 mmHg and/or DBP <50 mmHg.
f. Left ventricular cardiac ejection fraction (LVEF) <50%, calculated by ECHO or MUGA. Where LVEF% cannot be calculated on ECHO the following applies:
    i. if the LV function is normal, the patient is eligible;
    ii. If mild LV dysfunction is noted, the patient must have a MUGA scan or cardiac MRI and the LVEF be ≥50% to be eligible;
    iii. If moderate to severe LV dysfunction is reported on ECHO, the patient is ineligible for study entry;

10. With the exception of alopecia, any unresolved toxicities from prior therapy greater than CTCAE grade 1 at the time of starting study treatment.
11. Elevated Alkaline phosphatase (ALP) is not exclusionary if due to the presence of bone metastasis or if liver function is otherwise considered adequate in the investigator's judgement.
12. Proteinuria >3+ on dipstick analysis or ≥3.5 g/24 hours or a urine protein/creatinine ratio >3.5.
13. Refractory nausea and vomiting, chronic gastrointestinal diseases, inability to swallow the formulated product or previous significant bowel resection that would preclude adequate absorption of AZD5363;
14. History of hypersensitivity to active or inactive excipients of AZD5363 or fulvestrant;
15. Judgment by the Investigator that the patient should not participate in the study if the patient is unlikely to comply with study procedures, restrictions and requirements.
16. Past medical history of interstitial lung disease, drug-induced interstitial lung disease, radiation pneumonitis which required steroid treatment, or any evidence of clinically active interstitial lung disease.
17. Evidence of dementia, altered mental status or any psychiatric condition that would prohibit understanding or rendering of informed consent.
18. Participation in another clinical study with an investigational product (IP) during the last 30 days.
19. Known immunodeficiency syndrome;
20. Inability or unwillingness to comply with study procedures, including the inability to take regular oral medication.
21. Concomitant medication unsuitable for combination with trial medication including certain commonly used antiemetics and statins (see Appendix 3). Treatment with coumarins such as warfarin is not permitted in the study.
22. Previous or concomitant malignancies at any other site with the exception of the following:
    a. Benign basal cell carcinoma.
    b. Benign low grade transitional cell carcinoma of the bladder.

Other effectively treated malignancy that has been in remission for more than 5 years and is considered to be cured.

Note: A list of non-permitted medications can be found in section 7.6 and Appendix 3.

The patient's research nurse and/or doctor will screen the patient to ensure that they meet the eligibility criteria. The PI or co-investigator must confirm the eligibility of a patient in the patient's medical notes and on the enrolment CRF prior to enrolment.

6.3 Restrictions

The following restrictions apply while the patient is receiving study treatment and for the specified times before and after:

1. Patients who are blood donors should not donate blood during the study and for three months following their last dose of study treatment.
2. Patients should avoid excessive sun exposure and use adequate screening protection. The use of sun-beds and tanning booths should be avoided.
3. On blood glucose assessment days (incorporating clinical chemistry and glucose evaluations) it is requested that patients refrain from calorific intake for a minimum of 8 hours prior to the morning dose of AZD5363 / placebo.
4. Each week from Cycle 1 Day 15 onwards participants will be required to carry out a urine glucose assessment by dipstick first thing in the morning and before taking AZD5363 on day three of AZD5363/placebo treatment. If a positive result is observed, they must contact the clinic for further investigation of this result.

6.4 Registration and randomisation

6.4.1 Phase 2 (Stages 2, 3 and 4)

After consent but prior to registration (cycle 1 day 1) and randomisation (cycle 1 day 15) the participant's research nurse and/or doctor should contact WCTU to request a sample number to be assigned to that participant's tissue block and blood samples.
The participant's research nurse and/or doctor will follow the steps above (section 6.2) to screen the participant and ensure that they meet the trial eligibility criteria. Registration and randomisation will be performed through logging in to the Interactive Web Response System (IWRS) for the trial. An IWRS user guide will be provided to sites, see section 6.4.2. The IWRS will require confirmation of the eligibility criteria before registering the patient. The participant trial number will be generated by the IWRS system and confirmed by email. This should be recorded on the participant registration form.

Once sites are open to phase 2 and have been given IWRS log-ins, they should send the IWRS confirmation form to the WCTU (provided in the site file), which will confirm they are able to log in and have the correct access to forms including randomisation and unblinding.

For all patients registered in phase 2, randomisation can only take place once the results of the tests for PTEN and/or PIK3CA status have been returned to WCTU [redacted] The IWRS system will require the result of at least one of the tests to be entered prior to randomisation. The WCTU will contact sites to inform them of the results and that the patient is ready to be randomised. At randomisation, the PI should ensure that the participant still meets the trial eligibility criteria. The top copy of the participant randomisation form should be returned to the WCTU within four weeks.

1. Following confirmation of consent, call the WCTU [redacted] to obtain sample numbers for requesting the release of the tissue block and blood sample collection. This tissue will be used to confirm the PIK3CA mutation status following tumour DNA extraction and PTEN status by IHC analysis. These data will be used for patient stratification. The blood samples will be used to test cfDNA for PIK3CA status; these results will be used for stratification if the tumour analysis is not available.
2. Contact the local pathologist to arrange for the fast-track release of the participant's tumour sample blocks, to be sent to the following address:

   [redacted]

3. **Two 10ml blood samples must be taken from the patient immediately after consent. The first will be used to test cfDNA for PIK3CA status. The second will be stored for future correlative translational studies.** Blood should be collected into Cellsave/Streck tubes, inverted 10 times and placed immediately into first-class delivery pre-paid safeboxes provided by the WCTU. Blood samples can only be sent Monday to Thursday to ensure that they can be processed rapidly on receipt.

[redacted]

[redacted]

4. An additional 10ml blood sample may be requested at certain sites. This sample will be processed at site to extract the plasma which will be placed into frozen storage until collection is arranged by WCTU. This plasma sample will be analysed at the AWGL and the results compared to the corresponding 10mL blood sample collected for cfDNA PIK3CA mutation analysis.

5. Once eligibility is confirmed, complete the registration Case Report Form (CRF) and register the patient by logging in to the IWRS.

6. The following tests must be performed within 7 days prior to start of fulvestrant treatment (registration) to confirm patient eligibility:

   - ECG and QTc evaluation x 3 (taken 1 minute apart)
   - Clinical chemistry: serum levels of sodium, magnesium, potassium, urea, creatinine, albumin, alanine aminotransferase (ALT) or aspartate aminotransferase (AST), alkaline phosphatase (ALP), bilirubin, calcium
   - Full blood count (FBC): haemoglobin, white cell count, ANC, platelets
   - Random glucose
   - Prothrombin Time (PT)

Participants should start treatment with fulvestrant as soon as possible following registration. This should ideally be early in the week (Monday or Tuesday) to facilitate administration of AZD5363/placebo and testing of urine +/- blood glucose during the working week.

NB in phase 2, stage 4: If after the TSA of 40 participants with PIK3CA/PTEN wildtype tumours indicates that recruitment should be restricted to participants with non-wildtype tumours only, this will be based on the results of the cfDNA blood test, which will be available within 1 to 2 working days of receipt of the blood/plasma sample at AWGL. When the result of the cfDNA test is sent to WCTU, the WCTU will send the site a formal notification of the result to confirm eligibility. This should be filed in the investigator site file. No further data collection will be required for registered patients who are ineligible based on the cfDNA test result and any remaining blood/plasma sample will be destroyed by the All Wales Genetic Laboratory. At this stage 4 in the trial, the results of the cfDNA analysis should be available before the site requests the archival tumour tissue.

6.4.2 Treatment pack allocation using the IWRS website

IWRS will be used in phase 2 (stage 2, 3 and 4) of the trial only. Each centre will be provided with a user guide (see the Cenduit “Study Site User Guide”) and login details for the IWRS. The centre will enter the trial number and the treatment code number via the web interface. Once the treatment packs have been allocated, an email confirming the treatment kit numbers to be dispensed will be sent to the research

nurse, the site pharmacist and the WCTU. Each centre will be provided with kits of packaged drugs. The packaging and tablets/capsules will be identical in appearance for both active and matching placebo treatments. The label attached to each package of blinded study material will have a unique treatment kit number that is linked to the randomisation scheme.
The randomisation is centralised, and kit numbers will not be sequential within a centre. If a participant is given the incorrect kit, the participant should continue to take the medication they have been allocated. The site must inform the IWRS immediately after the error is identified.

The centre will inform the participant's GP of the participant's enrolment, if the participant gives consent to do so. It may be possible for participants to be recruited into other clinical trials, but this should be discussed with the CI via the WCTU before this will be considered.

7.0 Trial treatments

Fulvestrant

Fulvestrant is a non-investigational medicinal product (NIMP) in this trial, as it will be used within its marketing authorisation. It is administered intramuscularly slowly as two x 5ml (250mg) injections, one into each buttock. Fulvestrant 500 mg will be given on day 1 of every 28 day cycle (± 3 days window). An additional loading dose will also be delivered on Cycle 1 Day 15 (± 3 days window).

AZD5363 / matching placebo

AZD5363 is the investigational medicinal product (IMP) in this trial. A twice daily regimen of an oral formulation given on an intermittent weekly dosing schedule (4 days on/ 3 days off), starting on Cycle 1 Day 15.

Where possible, all doses of AZD5363/placebo should be taken at approximately the same time each day, in a fasted state (water to drink only) from at least 2 hours prior to the dose to at least 1 hour post-dose. Please also refer to Restrictions (section 6.3) for further guidance regarding participant fasting status on study assessment days.

If vomiting occurs no additional capsules/tablets should be taken. Patients should miss this dose and proceed to next dose.

Should a participant miss a scheduled dose, the participant will be allowed to take the dose up to a maximum of 2 hours after the scheduled dose time. If greater than 2 hours after the scheduled dose time, the missed dose should not be taken and the participant should take their allotted dose at the next scheduled time. If a participant needs to take the dose earlier, for whatever reason, the participant can take the dose up to 2 hours earlier than the scheduled dose time. The participant should make every reasonable effort to take the AZD5363/placebo capsule/tablet(s) on time, a diary card will be provided to the participant to help keep a record of this.

If a participant is unable to swallow the tablets due to toxicity, AZD5363/placebo treatment may be temporarily suspended until the toxicity is resolved. If the tablets are stopped for more than 28 continuous days, the participant should end all trial treatment.

7.1 Treatment Schedules

Participants will first receive fulvestrant on day 1 and AZD5363/placebo will start on day 15 of Cycle 1. Further treatment will be according to the schedule diagram below (figure 3). The order in which the two drugs are given is not critical but investigators are strongly urged to maintain participants on this schedule.

Figure 3 – Dosing schedule

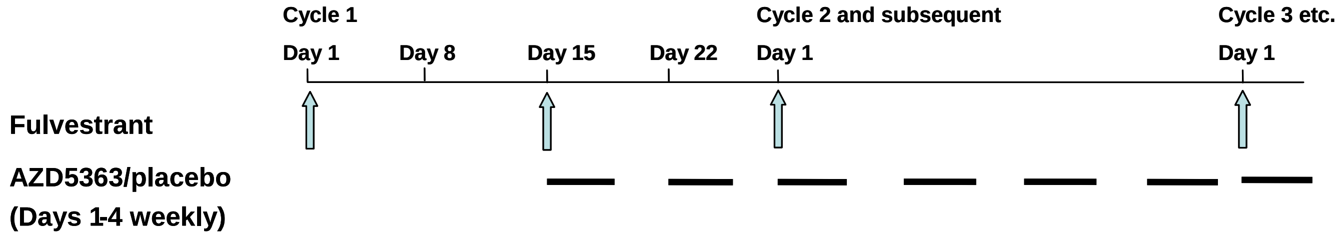

Participants should continue on treatment with AZD5363/placebo and/or fulvestrant until RECIST 1.1 defined objective progression or until a treatment discontinuation criterion is met.

7.2 Dose delays and modifications

- If fulvestrant is discontinued for any reason, the participant must also end AZD5363/placebo treatment. The participant must continue all follow up assessments in accordance with the schedule of assessments.

- If AZD5363/placebo is discontinued for reasons other than disease progression, the participant may continue on fulvestrant alone at the investigator's discretion. The participant must continue being scanned for RECIST 1.1 assessment in accordance with the schedule of assessments until objective disease progression or withdrawal from fulvestrant. The participant will no longer be required to be assessed for hyperglycaemia and proteinuria, and therefore glucose assessments and urine dipstick assessments can be stopped. Until disease progression, no additional anticancer therapy may be added to single agent fulvestrant to replace the discontinued therapy.

- If a participant becomes amenable for surgery to remove the primary tumour or a metastasis, surgery and/or adjuvant treatment with fulvestrant/AZD5363/ placebo is permitted. The participant must continue being scanned for RECIST 1.1 assessment post-surgery until objective disease progression.

7.3 Management of toxicities

In the event of an AE which the investigator considers to be related to the administration of study treatment (fulvestrant or AZD5363/placebo), supportive therapy should be given at the discretion of the investigator. We recommend that nausea and vomiting are treated with cyclizine or metoclopramide. Note that aprepitant and haloperidol are permitted but may interact with AZD5363 and should be used with caution (see Appendix 3).
Inflammation of the mouth and lips is very common in patients taking AZD5363. It can be managed with treatments such as mouthwashes and steroid-based creams.
In addition, the investigator may decide that dosing of study treatment should be temporarily interrupted or study treatment permanently discontinued as per the guidelines outlined below.

NB. All AE and additional treatment should be reported on the CRF.

AZD5363/placebo

Please see Appendix 4 for details of dose adjustments for:

- General toxicities
- Diarrhoea
- Skin reactions (including dry skin and pruritus)
- Hyperglycaemia and management with metformin (please also see section 7.7 for use of metformin)
- Hepatic toxicity
- High QTc
- Proteinuria

In the event of suspected toxicities associated with receipt of AZD5363/placebo, the investigator may wish to temporarily suspend dosing. If receipt of AZD5363/placebo is suspended for >28 continuous days, AZD5363/placebo should be permanently discontinued.

Phase 2

Any toxicity observed during the course of the study that is suspected of being related to AZD5363/placebo may be managed at the discretion of the investigator by dose interruption or by dose reduction according to Table 1. Please also see Appendix 4. Repeat dose interruptions are allowed as required, however reductions beyond the 2nd reduction should first be discussed with the Chief Investigators.

Table 1 - AZD5363/placebo dose reductions

AZD5363/placebo starting dose	1st dose reduction	2nd dose reduction
400mg bd	320mg bd	240mg bd

Dose interruptions may also be considered after discussions with the Chief Investigators for personal reasons such as holidays.

All dose modifications and interruptions (including any missed doses due to AEs), and the reasons for the dose modifications/interruptions are to be recorded in the CRF.

Fulvestrant

If an investigator feels that unacceptable toxicity can reasonably be attributed to fulvestrant, or if there are physical difficulties with administration of bilateral injections, a single dose reduction to the previously licensed dose of 250mg every 28 days may be allowed. However, this should first be discussed with the Chief Investigators as fulvestrant 250mg has been shown in randomised trials to be inferior to the 500mg dose.

7.4 Drug supply and distribution

AstraZeneca will fund the supply of both the investigational product (AZD5363 or matching placebo) as a solid oral formulation (tablets). The non-investigational product (Fulvestrant) will be supplied in pre-filled syringes:

Identity of investigational product

Investigational product	Form	Dosage strength	Manufacturer
AZD5363	Tablet	80mg and 200mg	AstraZeneca
Placebo to AZD5363	Tablet	N/A	AstraZeneca

AZD5363/placebo in a solid oral formulation will be supplied in white high density polyethylene (HDPE) Child Resistant bottles.

In Phase 2 (Stages 2, 3 and 4), when a site is first activated it will be provided with an initial supply of fulvestrant syringes and AZD5363 in blinded “kits” (bottles) of capsules/tablets and re-supply is automatic

when a site's stock falls below a minimum level. An on-line system called IWRS - for which a user guide will be provided (see the Cenduit "Study Site User Guide") – is used to:

- Confirm receipt of drug shipment (site Pharmacy will need do this)
- Dispense AZD5363 IMP (bottles) and NIMP (cartons) during treatment (Research Nurse/Pharmacist will do this) and receive kit number/s that should be allocated
- Withdraw/Unblind participants

AZD5363 will be supplied by AstraZeneca will be packaged, labelled and distributed [redacted] in accordance with local regulations and Good Manufacturing Practice, stating that the drug is for clinical use only and should be kept out of the reach and sight of children. Each bottle will contain 30 AZD5363 80mg or placebo tablets or 60 AZD5363 200mg or placebo tablets.

Fulvestrant will be supplied by AstraZeneca will be packaged and distributed [redacted] in accordance with local regulations and Good Manufacturing Practice.

Site to site transfers of AZD5363/placebo and fulvestrant within the same Trusts/Health boards are permitted as long as local procedures meet all trial associated regulatory requirements.

A detachable peel-off label will be affixed to each container and will contain space for participant trial ID number and date of dispensing to be completed and attached to the participant drug accountability case record form (CRF) at the time of dispensing.

Participants will be supplied with sufficient medication for each visit. There will be sufficient AZD5363 in the bottle to cover the visit window.

7.5 Drug accountability

Storage

Both AZD5363, fulvestrant and placebo must be kept in a secure place under appropriate storage conditions. A description of the appropriate storage and shipment conditions are specified on the product labels. Study treatment must be kept out of the reach and sight of children.

- AZD5363 and placebo should be stored at <30°C. Report any temperature excursions to WCTU as soon as they are discovered and quarantine the stock. WCTU will confirm whether the stock can be used, and if stock cannot be used, then the IWRS will be updated to allow for re-supply.
- Fulvestrant should be refrigerated at 2 to 8 ^{o}C. It should be stored in the original packaging and protected from light. Temperature excursions should be managed according to the SPC.

Accountability

The medication provided for this study is for use only as directed in the protocol. It is the investigator's responsibility to establish a system for handling the IMP and NIMP to ensure that:

- Deliveries of IMP and NIMP from AstraZeneca are correctly received by a responsible person (e.g. Pharmacist or suitable pharmacy designee) and are handled and stored correctly and safely. Receipt should be acknowledged through the IWRS system, or by returning the study drug receipt form in Phase 1
- Study IMP is dispensed only to study participants, and in accordance with the protocol
- Participants may return any unused AZD5363 IMP and all empty containers to the investigator

- A dispensing record (which will include the identification of the participant to whom the investigational product was dispensed, the date of dispensing, the quantity of investigational and non-investigational product dispensed, and the date and quantity of any unused IMP and NIMP returned to the pharmacy) is accurately maintained. Any discrepancies must be accounted for on the appropriate form. This record is in addition to any drug accountability information recorded in the CRF
- It must be possible to reconcile delivery records with dispensing records and records of destroyed or returned stock

In the case that the study IMP or NIMP is damaged during storage, please contact WCTU for reconciliation and replacement. If the study drug is damaged on arrival, please report it via IWRS (see the Cenduit "Study Site User Guide").

The participants will be given a diary card to keep track of the AZD5363 /placebo capsules or tablets they have taken. This should not be returned to the WCTU but should be used to help accurately complete treatment compliance and toxicity CRFs.

Unused or returned IMP from participants should be destroyed locally at site following local procedures after authorisation from WCTU. The number of tablets returned should be counted and recorded on the accountability log.

At the termination of the study or at the request of the sponsor, all unused drugs should be accounted for and destroyed locally at the study sites. Certificates of delivery and destruction must be signed and copies retained in the ISF.

7.6 Concomitant medications

The administration of all medication (including IMPs) must be recorded in the appropriate sections of the case report form (CRF). Participants must be instructed that additional medication should not be taken without the prior consent of the investigator. If medically feasible, participants taking regular medication - with the exception of potent inhibitors or inducers or substrates of CYP3A4 or substrates of CYP2D6 – should be maintained on it throughout the study period. Other anti-cancer agents and investigational agents should not be given while the participant is on study treatment.

Radiation for palliation at focal sites is permitted.

<u>Permitted concomitant medications / procedures</u>

- Blood transfusions are allowed at any time during the study.
- Granulocyte colony stimulating factors should not be used for secondary prophylaxis of AZD5363 induced neutropenia.
- Participants may receive bisphosphonates or denosumab for the treatment of bone metastases at any point before the start of the study but once the study has commenced starting such treatment would need to be discussed with the CI's.
- Participants may receive Domperidone for nausea and vomiting if alternatives are not appropriate and with appropriate monitoring for potential side effects.
- Participants may take corticosteroids, however, if initiated then an analysis for Glycosuria will be required 3 days following initiating steroids and managed as Appendix 4, management of hyperglycaemia.

- Metformin can be used for the management of hyperglycaemia occurring in participants participating in studies of AZD5363. Metformin should be given with care when used in combination with AZD5363 due to inhibition of OCT2. See section 7.7 for details.

Non-permitted concomitant medications (Appendix 3)

Treatment with coumarins such as warfarin is not permitted in the study. Therapeutic anticoagulation with low molecular weight heparin (LMWH) is permitted.

Potent CYP3A4 inhibitors may increase exposure to AZD5363 [redacted] and are strongly discouraged by AstraZeneca:

- Ketoconazole
- Protease inhibitors (danoprevir, ritonavir, saquinavir, indanavir, tapranavir, telaprevir, elvitegravir, lopinavir, nelfinavir, bocepravir)
- Cobicistat
- Conivaptan
- Nefazodone
- Mebefradil
- Itraconazole
- Posaconazole
- Voriconazole
  Clarithromycin
- Telithromycin
- Troleandomycin

Potent CYP3A4 inducers may reduce exposure to AZD5363 [redacted] and are strongly discouraged by AstraZeneca:

- Phenobarbital
- Carbamazepine
- Phenytoin
- Rifampicin
- Rifabutin
- Mitotane
- Enzalutamide
- St John's Wort

Agents that are significantly metabolised by CYP3A4 that AstraZeneca strongly recommend are not combined with AZD5363:

- Alfentanil
- Cyclosporin
- Diergotamine
- Ergotamine
- Fentanyl
- Sirolimus
- Tacrolimus
- Atorvastatin
- Lovastatin
- Simvastatin
- Cerivastatin
- Carbamazepine

Agents that are significantly metabolised by CYP2D6 that AstraZeneca strongly recommend are not combined with AZD5363:

- Amitryptyline
- Desipramine
- Trimipramine
- Doxepin
- Atomoxetine
- Metoprolol
- Nefazodone
- Nebivolol
- Perphenazine
- Tropisetron
- Tolterodine

Agents that are sensitive to combined CYP3A4 and CYP2D6 inhibition that AstraZeneca strongly recommend are not combined with AZD5363:

- Haloperidol
- Tramadol

For a complete list of these drugs and washout periods required, in addition to drugs that should only be used with caution refer to Appendix 3.

7.7 Use of metformin

Metformin is currently recommended for the management of hyperglycaemia occurring in patients participating in studies of AZD5363 (see Appendix 4 for indication threshold to start metformin) Investigators should exercise caution in the dosing and management of patients receiving the metformin/AZD5363 combination and must be vigilant for signs of renal impairment and metformin toxicity, such as lactic acidosis and hypoglycaemia, namely: lethargy, hypotension, poor urine output, drowsiness, irritation, tachypnoea, sweating, diarrhoea, and vomiting.

Metformin should only be given on the days when AZD5363 is also administered (the half-life of AZD5363 is approximately 8-15 hours), and should be withdrawn when treatment with AZD5363 is withdrawn, unless otherwise clinically indicated.

AZD5363 has been found in an in vitro assay to inhibit Organic Cation Transporter 2 (OCT2), found in the human kidney. In a clinical setting this has the potential to increase a patient's serum creatinine level, and also to increase the plasma levels of drugs known to be excreted by this transporter, including metformin. Due to this potential interaction, when taking both AZD5363 and metformin concurrently patients should attend the clinic for monitoring of serum creatinine at least once per week for the first three weeks after initiation of metformin.

7.8 Unblinding procedure

Individual treatment kit codes, indicating the treatment randomisation for each randomised participant, will be available to the investigator(s) or pharmacists from the IWRS. The kit code will be different for each bottle of drug. 24 hour unblinding can be performed via the IWRS system. The nominated site pharmacists, FAKTION research nurses and the PI all have IWRS accounts. In the event that the investigator (or pharmacist) is not available to unblind the following Cenduit 24 hour helpline is provided: +44 140 334 2316. Routines for this will be described in the IWRS user manual that will be provided to each centre.

Unblinding must not be performed except in medical emergencies when the appropriate management of the participant necessitates knowledge of the treatment randomisation. If the treatment code is broken then the investigator(s) must document and report to WCTU immediately. Once unblinded, it will not be possible to dispense further AZD5363/placebo or fulvestrant to the patient, and the end of treatment form should be completed. The patient can continue in the trial follow-up. In the event that the participant cannot be unblinded, it should be assumed that they have been treated with AZD5363.

8.0 Schedule of Trial treatments and Assessments

Participant follow-up visits should be planned according to the schedule in 8.1. Pharmacokinetic samples should be taken for all participants, please see section 11.6 for details.

8.1 Schedule of trial treatments and assessments

	Screening (after consent)			Cycle 1 Day 1	Cycle 1 Day 15	Cycle 2 Day 1[2]	Cycle 2 Week 4	Cycle 3 Day 1	Cycle 4 Day 1	Cycle 4 week 4	Cycle 5 Day 1	Cycle 6 Day 1	Cycle 6 week 4	Every 3 cycles, from Cycle 7[3]	End of Treatment	30 days post End of Treatment	3 monthly follow-up after Cycle 26
**Week**	-4[1]	-4 - 0	-1 - 0	0	1-2	4		8	12		16-			24-			
**Treatments**																	
Fulvestrant administration				X	X	X		X	X		X	X*					
AZD5363/placebo days 1-4 every week					X	X	X	X	X	X	X	X*	X	X			
**Assessments**																	
Translational Blood Sample Collection[4]	X							X							X		
Request archival tumour[5]	X																
Inclusion / exclusion criteria		X	X														
Informed consent		X															
Medical history		X													X		
Physical exam & vital signs[6]		X		X	X	X		X	X		X	X		X	X	X	
Disease evaluation (clinical)														X	X		
Disease evaluation (scan)[7]		X					X			X			X	X			X
ECG/QTc evaluation x 3 (1 min apart)			X	X	X	X		X						X			
MUGA/echocardiogram		X															
Serum biochemistry & full blood count[8]			X	X	X	X		X	X		X	X		X			X
PT and magnesium[8]			X														
Fasting glucose		X				X		X									
Random glucose[9]			X	X	X				X		X	X		X			
Urine glucose and protein			X	X	X	X		X	X		X	X		X			
Glycosylated haemoglobin		X						X			X			X			
FSH and estradiol		X															
Lipids		X						X			X			X	X		
Randomisation					X												
Adverse Events and toxicity [10]			X	X	X	X		X	X		X	X		X	X	X	X
Concomitant medication record		X		X	X	X		X	X		X	X		X	X	X	
Pharmacokinetic samples[11]					X	X		X									

1 Request samples immediately after consent.
2 All cycle assessments should be done on day 1 before the start of the cycle treatment. However, for convenience all assessments can be done within 72 hours of starting treatment. Participants will also be seen on days 15 of cycle 1.
3 All cycle assessments (except CT scans – see 7) should be done on day 1 before the start of the cycle treatment. However, for convenience all assessments can be done within 72 hours of starting treatment. If participants have ended treatment due to disease progression only death data is followed up. If participants have ended treatment for another reason and have not started another treatment disease progression and death data will be followed up.
4 2 x 10ml translational blood samples to be collected immediately after consent, week 8, and upon progression. An additional 10ml screening blood sample may be requested, to be spun and plasma stored frozen according to the laboratory instructions provided in the separate translational pack.
5 Archival tumour blocks should be requested from local pathology laboratories immediately after consent with an optimum turnaround time of results in 14 days.
6 Including ECOG performance status, weight, blood pressure, and pulse.
7 To include cross-sectional imaging of chest and abdomen (and, if clinically indicated, pelvis) during screening, and at weeks 8, 16, 24, then weeks 36, 48 and 60. After week 60, CT scans should be performed as per clinical practice. Other RECIST v1.1 compatible imaging will be allowed e.g. MRI as long as the same method is then used throughout the study.

8 Bloods include magnesium (screening only), sodium, potassium, urea, creatinine, albumin, ALT or AST, ALP, bilirubin, calcium, PT (screening only).
9 Non- fasting sample taken prior to AZD5363 and Fulvestrant dosing.
10 N.B. Serious Adverse Events (SAE) will be collected in real time via a designated SAE email address.
11 3ml blood to be taken for pharmacokinetics analysis at Cycle 1 Day 15 prior to first AZD5363 and then at Cycle 2 Day 1 and Cycle 3 Day 1 prior to AZD5363, see section 11.6 for details.

*Note 28 day treatment cycles continue as before.

8.2 Completion of CRFs

Phase 2

The following CRFs should be faxed/emailed to WCTU as soon as the information becomes available (within 24 hours):

- PIK3CA Testing and Translational Blood Sample Collection (once research nurse has completed section A)
- Archival Tumour Tissue Sample Collection for PIK3CA and PTEN Testing (as soon as research nurse has completed section A and again when local pathologist has completed section B)
- Withdrawal CRF
- Death CRF

The top copy of all completed CRFs should be returned to the WCTU by post within four weeks of the visit. The remaining copy is to be retained at the local site. If carbon copy CRFs are not provided fax/email or send a photocopy to WCTU instead.

In accordance with the principles of GCP, the PI is responsible for ensuring accuracy, completeness, legibility and timeliness of the data reported to the WCTU in the CRFs.

CRF pages and data received by the WCTU from participating trial sites will be checked for missing, illegible or unusual values (range checks) and consistency over time.

If missing or questionable data are identified, a data query will be raised on a data clarification form. The data clarification form will be sent to the relevant participating site. The site shall be requested to answer the data query or correct data on the data clarification form. The CRF pages should not be altered.

All answered data queries and corrections should be signed off and dated by a delegated member of staff at the relevant participating site. The completed data clarification form should be returned to the WCTU and a copy retained at the site along with the participants' CRFs.

The WCTU will send reminders for any overdue data. It is the site's responsibility to submit complete and accurate data in timely manner.

9.0 Safety reporting and pharmacovigilance

The Principal Investigator is responsible for ensuring that all site staff involved in this trial are familiar with the content of this section.

The following definitions are in accordance with the Medicines for Human Use (Clinical Trials) Regulations 2004 (SI2004/1031) (as amended) and EU Directive 2001/20/EC.

Term	Definition
**Adverse Event (AE)**	Any untoward medical occurrence in a patient or clinical trial participant administered a medicinal product and which does not necessarily have a causal relationship with this treatment. An adverse event can therefore be any unfavourable and unintended sign (including an abnormal laboratory finding for example), symptom or disease temporally associated with the use of a medicinal product, whether or not considered related to the medicinal product.
**Adverse Reaction (AR)**	Any untoward and unintended response in a clinical trial participant to an investigational medicinal product which is related to any dose administered to that participant
**Serious Adverse Event (SAE)**	Any adverse event that -<br>• Results in death<br>• Is life-threatening*<br>• Required hospitalisation or prolongation of existing hospitalisation**<br>• Results in persistent or significant disability or incapacity<br>• Consists of a congenital anomaly or birth defect<br>• Other medically important condition ***<br>In addition for the purposes of this trial the following events will also be considered SAEs and must be captured on the SAE form and reported to the WCTU with 24 hours of knowledge of the event:<br>• Any event of liver injury meeting the criteria for potential Hy's Law (PHL), see Appendix 5<br>For the purposes of this trial the following events will not require immediate reporting<br>• Death due to disease progression<br>• Hospitalisation due to disease progression

	These should be completed in the participants notes and on the relevant toxicities CRF page and forwarded to the WCTU in the normal timeframes for CRFs.
**Serious Adverse Reactions (SARs)**	Any SAE occurring in a clinical trial participant for which there is a reasonable possibility that it is **related** to the IMP at any dose administered.
**Suspected Unexpected Serious Adverse Reactions (SUSARs)**	A SAR, the nature and severity of which is not consistent with the Reference Safety Information (RSI) for the IMP.

***Note:** The term 'life-threatening' in the definition of serious refers to an event in which the trial participant was at risk of death at the time of the event or it is suspected that used or continued used of the product would result in the subjects death; it does not refer to an event which hypothetically might have caused death if it were more severe.

**** Note:** Hospitalisation is defined as an inpatient admission, regardless of the length of stay, even if the hospitalisation is a precautionary measure for continued observation. Pre-planned hospitalisation e.g. for pre-existing conditions which have not worsened, or elective procedures, does not constitute an SAE.

***** Note:** other events that may not result in death, are not life-threatening, or do not require hospitalisation, may be considered as an SAE when, based upon appropriate medical judgement, the event may jeopardise the participant and may require medical or surgical intervention to prevent one of the outcomes listed above.

9.1 Causality Assessments

The Principal Investigator (or another delegated medically qualified doctor from the trial team) and Chief Investigators (or another medically qualified doctor from the Trial Management Group) will assess each SAE to determine the causal relationship with the IMP, and will answer 'yes' or 'no' to the question "Do you consider that there is a reasonable possibility that the SAE may have been caused by the IMP?"

For SAEs causal relationship will also be assessed for other trial treatments (NIMPs) and procedures.

**IMPs:** AZD5363
**NIMPs:** Fulvestrant and permitted concomitant medications (see section 7.6)

The causality assessment given by the Principal Investigator (or delegate) cannot be downgraded by the Chief Investigator (or delegate), and in the case of disagreement both opinions will be provided.

A guide to the interpretation of the causality question is found in Appendix 6 of this clinical trial protocol.

9.2 Expectedness Assessments

The Chief Investigator (or another delegated appropriately qualified individual) will assess each SAE to perform the assessment of expectedness.

The expectedness assessment should be made with reference to the current Reference Safety Information (RSI) for the IMP in this trial. Expectedness decisions must be based purely on the content of the RSI; other

factors such as the participant population and participant history should not be taken into account. Expectedness is not related to what is an anticipated event within a particular disease.

SAEs which add significant information on specificity or severity of a known, already documented adverse event constitute unexpected events. For example, an event more specific or more severe than that described in the RSI is considered unexpected.

The table below lists the RSI that should be referenced

IMP	RSI to be used for expectedness assessment	Relevant section of RSI to be used for expectedness assessment
AZD5363	Investigator's Brochure	Section 5.4

9.3 SAE reporting

9.3.1 Participating Site Responsibilities

All SAEs must be reported immediately (and within 24 hours of knowledge of the event) by the PI at the participating site to the WCTU unless the SAE is specified as not requiring immediate reporting (see above). This includes SAEs related to IMPs and non-Investigational Medicinal Products (NIMPs).

The PI (or delegated medically qualified doctor from the trial team) should sign and date the SAE CRF to acknowledge that he/she has performed the seriousness and causality assessments.

A completed SAE form for all events requiring immediate reporting should be emailed to the WCTU within 24 hours of knowledge of the event. A separate form must be used to report each event, irrespective of whether or not the events had the same date of onset.

The participant will be identified only by trial number, date of birth and initials. The participant's name should not be used on any correspondence.

It is also required that sites respond to and clarify any queries raised on any reported SAEs and report any additional information as and when it becomes available through to the resolution of the event.

[redacted]

Serious adverse events should be reported from time of signature of informed consent, throughout the treatment period up to, and including 30 days after the participant receives their last dose of the IMP even if the participant has completely withdrawn from the study. Serious adverse reactions (such as long term side effects of trial treatment under investigation) should continue to be reported until resolution.

Adverse events (AE) should be graded using the NCI Common Terminology Criteria for Adverse Events (CTCAE) Version 4.03.

The toxicity grades should be recorded on the toxicity part of the CRF.

An SAE form is not considered as complete unless the following details are provided:

- Full participant trial number
- An Adverse Event / Adverse Reaction
- A completed assessment of the seriousness, and causality as performed by the PI (or another appropriately medically qualified doctor registered on the delegation log).

If any of these details are missing, the site will be contacted and the information must be provided by the site to the WCTU within 24 hours.

All other AEs should be reported on the CRF following the CRF procedure described in Section 8.2.

9.3.2 The WCTU responsibilities

Following the initial report, all SAEs should be followed up to resolution wherever possible, and further information may be requested by the WCTU.

The WCTU should continue reporting SAEs until 30 days after the participant receives their last dose of the investigational medicinal product. SAEs should also be reported for participants who have withdrawn (from any aspect of the study) up to 30 days after the participant received their last dose of the investigational medicinal product. All reported SAEs should be followed up until resolution irrespective of withdrawal.

Once an SAE is received at the WCTU, it will be evaluated by staff at the WCTU and sent to the Chief Investigator (or their delegate) for an assessment of causality and expectedness.

Investigator reports of suspected SARs will be reviewed immediately and those that are identified as SUSARs are reported to the MHRA, Main Ethics Committee and AstraZeneca.

9.4 SUSAR reporting

Velindre NHS Trust is undertaking the duties of trial Sponsor and has delegated to the WCTU the responsibility for reporting SUSARs and other SARs to the regulatory authorities (MHRA and relevant ethics committees) and to AstraZeneca as follows:

SUSARs which are fatal or life-threatening must be reported to the MHRA and MREC within 7 calendar days of receipt at the WCTU. Any additional, relevant information must be reported within a further 8 calendar days of submitting the initial report.

SUSARs that are not fatal or life-threatening must be reported to the MHRA and MREC within 15 days of receipt at the WCTU. Any additional, relevant information must be reported within a further 15 days.

N.B. There is no requirement for WCTU to report SUSARs to NIMPs to the MHRA except in the following instances:

- If the adverse reaction is suspected to be linked to an interaction between a NIMP and IMP, and is serious and unexpected, WCTU should report as a SUSAR due to the interaction with the IMP.
- If a SUSAR is suspected and might be linked to either a NIMP or an IMP and cannot be attributed to only one of these.
- If the adverse reaction due to the NIMP is likely to affect the safety of trial subjects then WCTU should report it to the MHRA and Main Ethics Committee in accordance with the relevant Standard Operating Procedure for reporting Urgent Safety Measures.

9.5 Unblinding for the purposes of SUSAR reporting

To enable processing of a SAR in this blinded trial, expectedness should be assessed initially using the assumption that the AZD5363 has been given to the trial participant.

If it is assessed as unexpected, as per the RSI of AZD5363, the SUSAR will be unblinded by the WCTU safety group prior to reporting to the MHRA and MREC.

If after unblinding, it is evident that the trial participant received AZD5363, but the event still meets the criteria for a SUSAR (i.e. unexpected as per the AZD5363 RSI, then it will be reported expeditely according to the requirements set out in section 9.4 above).

If after unblinding, it is evident that the trial participant received the placebo, this event will not require expedited reporting to the MHRA and MREC, unless in the opinion of the Principal Investigator or Chief Investigator the event was related to the placebo (for example an allergic reaction to an excipient).

9.6 Safety Reports

A list of all SARs (expected and unexpected) will be reported annually to the MHRA, Main Ethics Committee, trial sponsor and AstraZeneca in a Development Safety Update Report (DSUR). This report must be submitted within 60 days of the anniversary of the MHRA CTA approval date.

The WCTU will report a list of all SARs (expected and unexpected) and any other safety recommendations to all PIs every six months throughout the course of the trial. This frequency may be reviewed and amended as necessary. This reporting will be done via the Investigator safety report (ISR).

The WCTU will provide six monthly reports to AstraZeneca to include a summary of the SAE line listings.

The WCTU will produce and submit the DSUR for AZD5363.

9.7 Flowchart for Serious Adverse Event reporting

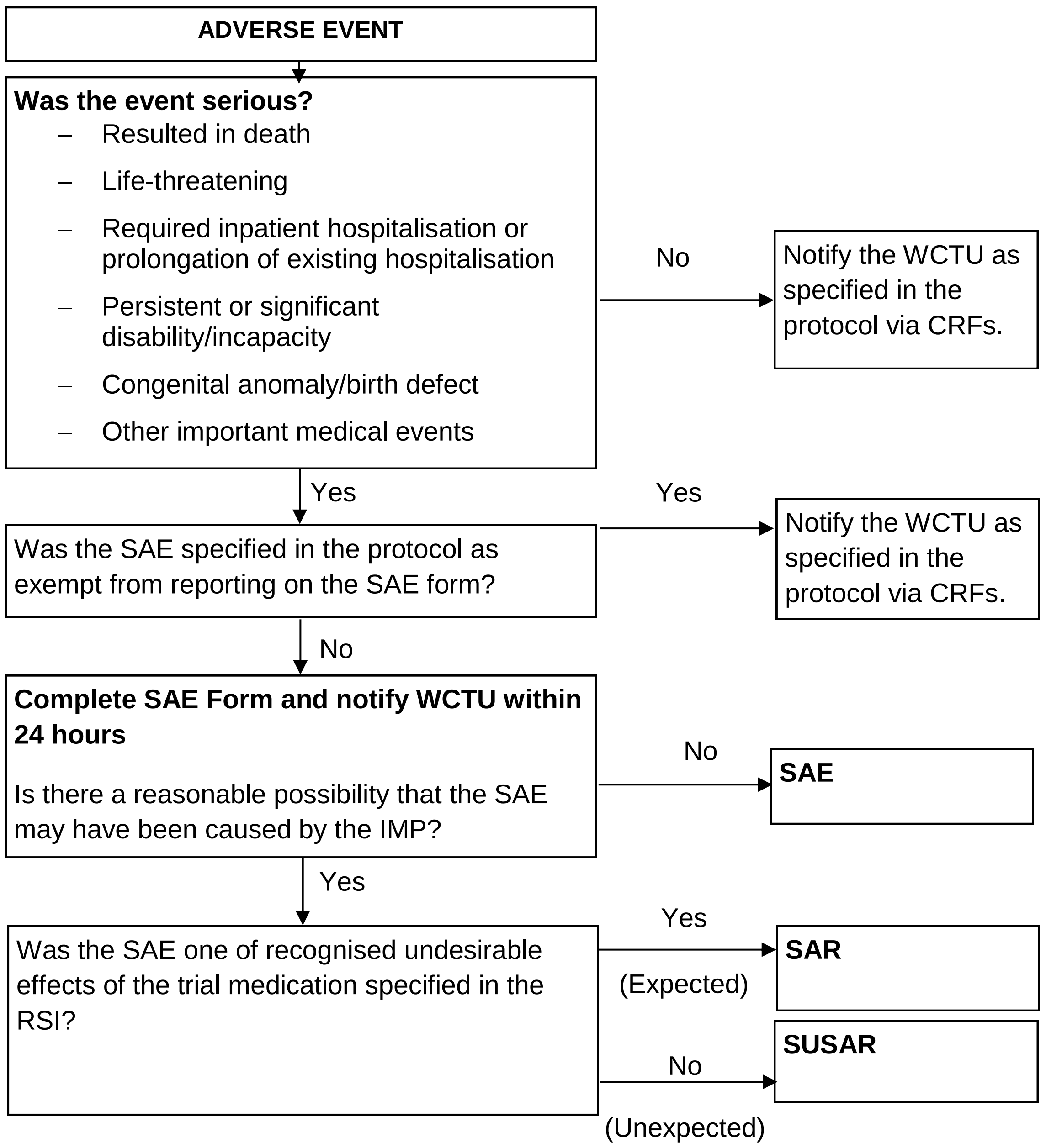

CRF Case Report Form
RSI Reference Safety Information
SAE Serious Adverse Event
SAR Serious Adverse Reaction
SUSAR Suspected Unexpected Serious Adverse Reaction
WCTU Wales Cancer Trials Unit

10.0 Trial monitoring and management

10.1 Monitoring

The clinical trial risk assessment has been used to determine the intensity and focus of central and on-site monitoring activity in the FAKTION trial. Higher intensity monitoring levels will be employed and are fully documented in the trial monitoring plan.

Investigators should agree to allow trial related monitoring, including audits and regulatory inspections, by providing direct access to source data/documents as required. Patient consent for this will be obtained.

10.1.1 Central monitoring

The WCTU will perform central data monitoring to review whether CRFs are returned on time, and that the protocol is being followed correctly in terms of whether scheduled assessments have been performed and toxicities and dose modifications have been recorded correctly.

10.1.2 Site monitoring

Investigators should agree to allow trial related monitoring, including audits and regulatory inspections, by providing direct access to source data/documents as required. Participant consent for this will be obtained. Routine and triggered monitoring visits will be performed, the frequency of which will be detailed in the separate trial monitoring plan.

10.2 Trial committees and trial management

The conduct of the trial is being overseen by the following committees:

1. Trial Management Group (TMG): The TMG will be responsible for the day-to-day running of the trial and will meet at least once every six months. The TMG members will include the CIs, other active trial investigators, WCTU representatives, and specialist advisors (e.g. Pharmacist, Statistician, consumer representative).

2. Independent Data Monitoring Committee (IDMC): The IDMC will consist of at least two independent Clinicians (not entering participants into the trial) and an independent Statistician who will review trial data approximately every six months. The remit of the IDMC will be to:

    a. Recommend after every meeting whether the accumulated data from the trial, together with results from other relevant trials, justifies continuing recruitment of further participants.

    b. Recommend after meeting to discuss the data from the pre-planned interim analysis at conclusion of Stage 3 as to whether to recommend discontinuation of recruitment, in all participants or in selected subgroups.

    Any decision to discontinue will be made only if the result is likely to convince a broad range of clinicians including PIs in the trial and the general clinical community. If a decision is made to recommend continuation, the IDMC will advise on the frequency of future reviews of the data on the basis of accrual and event rates. There will be two initial safety reviews of toxicities and SAEs after 20 and 40 participants have completed trial treatment in Phase 2. The IDMC will make confidential recommendations to the Trial Steering Committee (TSC).

3. Independent Trial Steering Committee (TSC): The TSC will be a committee of independent members that provides overall supervision of the trial. The role of the TSC is to act on behalf of the sponsor, to

provide overall supervision for the trial, to ensure that it is conducted in accordance with GCP, and to provide advice through its independent chairman. The TSC will review the recommendations from the IDMC and will decide on continuing or stopping the trial, or modifying the protocol. It will meet at least annually when it will consider each report of the IDMC, as well as results of other trials and new information which has arisen, and recommend appropriate action.

10.3 End of treatment and participant withdrawal

Participants and/or physicians may choose to end trial treatment; however, this does not necessitate participant withdrawal from the study.

Specific reasons for discontinuing study treatment are:

- Voluntary discontinuation by the participant who is at any time free to discontinue study treatment or their participation in the study as a whole, without prejudice to further treatment
- Safety reasons as judged by the investigator and/or sponsor including:
    - grade 4 hyperglycaemia
    - ALT or AST >8x ULN
    - or, ALT or AST >5x ULN for more than 2 weeks
    - or, ALT or AST >3x ULN and (total bilirubin >2x ULN or INR >1.5)
    - or, ALT or AST >3x ULN with the appearance of fatigue, nausea, vomiting, right upper quadrant pain or tenderness, fever, rash and/or eosinophilia (>5%)
- Severe non-compliance to protocol as judged by the investigator and/or sponsor
- Participant lost to follow-up
- Clinical disease progression
- Non-concordance with protocol treatment
- Incorrect enrolment i.e., the participant does not meet the required inclusion/exclusion criteria for the study

If a participant permanently discontinues trial treatment, follow-up visits and translational sample collection should be continued according the trial schedule where possible. If a participant withdraws consent to follow up, sample collection or data collection a withdrawal CRF should be faxed to the WCTU as soon as available (within 24h) with the hard copy to follow soon after. Participants do not have to give a reason for their withdrawal but sites should make a reasonable attempt to find out why. SAE reporting will continue for all trial participants for 30 days after the end of trial treatment regardless of the aspect of withdrawal and all SAE's will be followed up to resolution irrespective of timelines; this is for the purpose of patient safety.

Data and samples collected prior to a participant withdrawing consent for their collection will still be used for trial analysis by the WCTU. Participants who initially consented to be registered with the National Health Service Information Centre (NHSIC) or equivalent will remain on the system so that important research information on date and cause of death can be requested from NHSIC by the WCTU.

If a participant explicitly withdraws consent to have any data recorded, their decision must be respected and recorded on the withdrawal form. Details of the withdrawal form should be noted in the participant records and no further FAKTION CRFs should be completed for the participant. On receipt of a withdrawal form at the WCTU, the WCTU will contact the site to confirm exactly what aspects of the trial the participant has withdrawn from. SAE reporting will continue for all trial participants for 30 days after the end of trial treatment regardless of the aspect of withdrawal and all SAE's will be followed up to resolution irrespective of timelines.

10.4 Lost to follow up

If a participant is lost to follow up, the WCTU will request that the PI contacts the participant's GP to obtain information on the participant's status. Participants have the option to consent to NHSIC Flagging. This will entail completion of a separate consent form which will contain the participant name and will therefore be kept separate from the other data, and securely locked away. This will enable the WCTU to trace the participant's cause and date of death.

10.5 Protocol/GCP non-compliance

The PI should report any non-compliance to the trial protocol or the conditions and principles of GCP to the WCTU as soon as they become aware of it.

10.6 Trial closure

For the purposes of both MHRA and Research Ethics Committee approval, the study end date is deemed to be the date of last data capture. This will be the last patient's study visit, or when the required number (98) disease progression events have been reported, whichever is later. After this point, data on survival will be obtained from the NHS Information Centre flagging service.

10.7 Archiving

The TMF and ISF containing essential documents will be archived at an approved external storage facility for a minimum of 15 years. The WCTU will archive the TMF and TSFs on behalf of the Sponsor. The Principal Investigator is responsible for archival of the ISF at site. Essential documents pertaining to the trial shall not be destroyed without permission from the Sponsor. There is no requirement to retain the participant diary card at the end of the trial.

11.0 Statistical considerations

11.1 Randomisation

Randomisation will take place centrally via the IWRS. Participants will be randomised using the method of minimisation with a random element. The result of the PI3K kinase mutation status must be available prior to randomisation, as this is one of the criteria for the minimisation. This will ensure balanced treatment allocation by a number of clinically important stratification factors. Randomisation will have an allocation ratio of 1:1.

The tumour tissue results will be used to categorise each patient's PIK3CA and PTEN result for randomisation. Where the results of the tumour tissue are not available, the results of the blood analysis may be used for PIK3CA. A pre-specified algorithm has been developed in order to provide a consistent categorisation for any stratification disagreement at randomisation.

11.2 Outcome measures

11.2.1 Phase 2 - Primary outcome measure

- To establish the anti-tumour activity of the combination of AZD5363 with fulvestrant as measured by progression-free survival (PFS). This is the time from enrolment to any disease progression and/or any death, defined according to strict RECIST v1.1 (Appendix 1; www.recist.com) criteria. Lesions will be compared to baseline measurements to assess progression.

11.2.2 Secondary outcome measures

- Safety, tolerability and feasibility of use (the number of patients with adverse events and the number of patients requiring dose modifications)
- Objective response rate (ORR) and clinical benefit as assessed by RECIST 1.1 (Appendix 1)
- Overall survival (OS), time from randomisation to death with those still alive censored at date last seen
- The influence of activated PI3K pathway status (defined as mutation in PIK3CA found in archival tumour biopsy or plasma or low/absent expression of PTEN by IHC on archival tumour biopsy) on outcome in the two treatment groups. This will also include an exploratory TSA of the first 40 patients with wildtype tumours.
- Fulvestrant pharmacokinetics
- Exploratory biomarkers

11.3 Sample size calculation

The sample size was calculated for a Phase 2 screening design, based on a primary outcome of progression free survival, with a time-to-event hazard ratio of 0.65, and with 90% power and a one-sided significance of 20% and assuming an overall loss to follow-up of 10%. Assuming that the estimated PFS in the control arm is 5.4 months, and that we will have an 18 month accrual period and a six month follow-up, then a total of 98 events will be required overall. We aim to recruit 138 patients in order to detect 98 progression events.

If recruitment is restricted to the PIK3CA mutation positive group after 40 wildtype patients have been recruited and analysed, then we will need to record 70 events and therefore aim to achieve recruitment of 98 PIK3CA mutant patients, which will provide 90% power to detect a hazard ratio of 0.6.

11.4 Statistical analyses

A statistical analysis plan will be developed before the first analysis of the trial and will be signed off prior to unblinding the data for analysis.

A WCTU statistician will unblind the data prior to analyses for the safety run-in, IDMC analysis and final report. Unblinded interim reports will only be seen by the WCTU statistician and the IDMC. The final clinical study report will be unblinded.

Safety will be assessed by the IDMC when 20 and 40 patients have completed trial treatment.

The IDMC will also review the data after 40 confirmed wild type participants have been recruited in Stage 2 of the trial. The outcome variable for this analysis is change in tumour size at 8 weeks post registration, as assessed by RECIST v1.1 at baseline and 8 weeks. Full details of the analysis can be found in Appendix 2.

The size of the tumour analysis will be performed and if there is <10% difference between placebo and AZD5363 treated wildtype participants, then the eligibility criteria will be restricted to participants with PIK3CA mutant tumours only.

The trial data will be analysed when all participants have completed a minimum six months follow-up and at least 98 disease progression events are observed. Disease progression will be formally assessed according to the RECIST v1.1 criteria.

At the end of the trial both an intention-to-treat (all results analysed according to the participants' original trial arm allocation) and a per-protocol analysis will be carried out. We will attempt to trace participants who are lost to follow-up via their GP or through the NHS-IC. Where no information is available these participants will be censored at the date last seen.

Primary analysis will be of PFS described using Kaplan-Meier curves in both arms of the trial. The median PFS will be calculated for each arm of the trial, and then the logrank test will be then used to formally test the equality of the survivor functions. If the hazards are proportional, then Cox regression will also be performed to adjust the hazard ratio for the stratification factors.

11.5 Subgroup analyses

PFS, overall survival OS and objective response rate ORR will be analysed according to PI3k/PTEN pathway activation status. The definition of PI3k/PTEN pathway activation is as follows:

PI3K/PTEN pathway activated: at least one alteration observed in one of the markers i.e.

PIK3CA gene mutation in either cfDNA or tumour block analysis and/or low/reduced expression of PTEN analysed by immunochemistry (< 10% of tumour cells expressing PTEN at no more than 1+ level) independent of availability of remaining test results.

PI3K /PTEN pathway non-activated (wildtype): no mutations detected in PIK3CA gene in cfDNA or tumour block analysis and PTEN expression detectable in ≥ 10% of tumor cells at 1+ or in any tumour cells at >1+. Where PIK3CA mutation is un-interpretable in cfDNA but no mutation is found in tumour block DNA analysis, such a participant's tumour will be classified as pathway non-activated.

PI3K /PTEN pathway unknown: at least 1 un-interpretable result from analyses of the tumour block only (mutation or IHC) combined with no criteria of activation as defined above in any of the markers; does not meet the definition of PI3K pathway activated or non-activated.

12.0 FAKTION Translational research

12.1 Pharmacokinetic analysis of Fulvestrant

Pharmacokinetic analysis will be performed on patients in Phase 2 where possible. Blood (3ml) should be taken for pharmacokinetics analysis (PK) at Cycle 1 Day 15 prior to first AZD5363 administration and then at Cycle 2 Day 1 and Cycle 3 Day 1 prior to AZD5363. Samples should be collected into the lithium heparin tubes provided for PK analyses. Immediately invert the sample at least 10 times and then within 1 hour of collection centrifuge the samples at 1500g below 30°C (4°C preferred) for 10 minutes. Following centrifugation, use a disposable pipette to transfer all plasma into a 1.8 ml cryovial provided and labelled with key participant identifiers as detailed in the FAKTION laboratory manual. Ensure a clean pipette is used for each sample and ensure that the label is firmly attached.

The plasma samples will be stored at -20°C in an upright position within 30 minutes of plasma preparation and kept frozen at this temperature until shipment. These samples will be shipped to Covance for analysis. Arrangements for shipment will be provided in the separate FAKTION Laboratory Manual.

The minimum concentration (C_{min}) of fulvestrant will be measured in ng/ml at Cycle 1 Day 15, Cycle 2 Day 1 and Cycle 3 Day 1, prior to the administration of AZD5363/placebo and fulvestrant. The geometric mean will be compared between the AZD5363 and placebo groups to establish whether AZD5363 affects the C_{min} for fulvestrant.

12.2 Samples for mutational analysis and future translational research

Collection and analysis of exploratory blood-borne biomarkers

20ml venous blood will be taken at screening. This must be collected into two yellow and purple/black topped CellSave/Streck tubes provided by WCTU, inverted 10 times and placed immediately into first-class delivery pre-paid safeboxes provided by the WCTU. 10 ml will be used for cfDNA extraction from plasma and germ-line DNA extraction from buffy coat (to allow evaluation of new mutations) and 10ml will be stored for future translational research.

Some sites may be requested to collect an additional 10ml blood sample from patients at screening. The purpose of this additional sample and processing method is to compare the mutational PIK3CA status of the plasma sample processed at the time of collection with the blood sample collected in Streck/Cellsave tubes (and processed within 96h) and to check for any discordance. This 10ml blood sample must be spun immediately (within 30 minutes) at 1,000 RCF (Relative Centrifugal Force) for 10 minutes in a centrifuge to separate out the plasma. If not processed immediately, the sample collection container may be stored at 2-8°C if EDTA tube or at room temp for Streck/CellSave tubes, but must be processed within 2 hours. The plasma should then be removed into clean 1.5ml Eppendorf tubes in 1 ml aliquots. As much plasma as possible should be collected without disturbing the interface. Re-spin the plasma sample at 2,000 RCF for 10 minutes to ensure as many cells as possible have been removed from the plasma and transfer the upper / clear layer to a fresh tube. Each tube should be labelled with the FAKTION sample number, patient initials and date of collection. The tubes should be stored upright at -80°C until collection is arranged by WCTU.

An additional two x10ml sample for future translational research will be requested at week 8 and upon progression. These samples should also be collected into two yellow and purple/black CellSave/Streck tubes, inverted 10 times and placed immediately into first-class delivery pre-paid safeboxes.

[redacted]

Please remember to invert the CellSave/Streck tubes 10 times prior to sending; this will ensure full incorporation of preservative with blood.

13.0 Publication policy

Data from all centres will be analysed together and published as soon as possible. Individual participating PIs may not publish data concerning their participants that are directly relevant to questions posed by the trial until the TMG has published its report. The TMG will form the basis of the writing committee and will advise on the nature of publications, subject to sponsor requirements.

All potential contributors will have the opportunity to opt into a writing team. Criteria based on BMJ rules on *authorship* and *contributorship* (see http://www.bmj.com/about-bmj/resources-authors/article-submission/authorship-contributorship) will be used to acknowledge the level and nature of contribution of key individuals in publications arising from the project.

14.0 Informed consent, ethical and regulatory considerations

14.1 Ethical and other issues

This clinical trial protocol will be submitted to a Multi-centre Research Ethics Committee (MREC) that is legally recognised by the United Kingdom Ethics Committee Authority for review and approval. The approval of the MREC must be obtained before the start of a clinical trial or any trial procedures are conducted. The local Research and Development office (R&D) must also approve each institution, through the site specific information (SSI) process, before participants are recruited at that centre.

All substantial amendments to this trial protocol must be approved by the MREC responsible for the study, before the implementation of the amendments. Minor amendments will not require prior approval by the MREC.

If the trial is stopped due to adverse events it will not be recommenced without reference to the MREC responsible for the study.

The MREC will be notified within 90 days of trial completion. If the trial is terminated early, the MREC will be notified of this within 15 days.

A summary of the clinical trial report will be submitted to the MREC responsible for the study within one year of the completion of the last participant's final follow-up procedure.

The participant's consent to participate in the trial should be obtained after a full explanation has been given of the treatment options, including the conventional and generally accepted methods of treatment. All participants must be informed of the aims of the study, the possible adverse events, the procedures and possible hazards to which they may be exposed. They will be informed of the strict confidentiality of their participant data, but that their medical records may be reviewed for trial purposes by authorised individuals other than their treating physician.

Participant's consent will be sought to notify their GP of their involvement in the trial. Participants should be given sufficient time after being given the trial PIS to consider and discuss participation in the trial with friends and family. A contact number should be given to the participant should they wish to discuss any aspect of the trial. Following this, the enrolling investigator should determine that the participant is fully informed of the trial and their participation, in accordance with the principles of ICH-GCP. Participants should always be asked to sign a consent form. One copy should be given to the participant but the original copy should be kept in the study site file and a further copy should be kept with participant's hospital notes.

The right of the participant to refuse to participate in the trial without giving reasons must be respected. After the participant has entered the trial, the investigator must remain free to give alternative treatment

to that specified in the protocol, at any stage, if he/she feels it to be in the best interest of the participant. However, the reason for doing so should be recorded and the participant will remain within the trial for the purpose of follow up and data analysis according to the treatment option to which he/she has been allocated. Similarly, the participant must remain free to end the protocol treatment without giving reasons and without prejudicing his/her further treatment; follow-up, translational sample collection and data capture should be continued however the participant has the right to withdraw consent to any of these procedures for any reason and at any time. SAE reporting will continue for all trial participants for 30 days after the end of trial treatment regardless of the aspect of withdrawal and all SAE's will be followed up to resolution irrespective of timelines.

14.2 Regulatory status

The trial is being performed under a Clinical Trial Authorisation (CTA) from the MHRA. The Clinical Trials Authorisation (CTA), the approval of the MHRA, must be obtained before the start of the trial in accordance with Part 3, Regulation 12 of The Medicines for Human Use (Clinical Trials) Regulations 2004 (SI2004/1031).

14.3 Regulatory considerations

All substantial amendments to this Protocol must be approved by the MREC responsible for the study and MHRA, before the implementation of the amendments. Minor amendments will not require prior approval by the MREC and MHRA.

If the trial is temporarily halted it will not be recommenced without reference to the MREC responsible for the study and the MHRA.

The MREC and MHRA will be notified within 90 days of trial completion. If the trial is terminated early, the MREC and MHRA will be notified of this within 15 days.

A summary of the clinical trial report will be submitted to the MREC responsible for the study and MHRA within one year of the end of trial.

14.4 Research Governance approval

This trial protocol will be submitted through the Research Governance process of the host care organisation for review and approval. The Research Governance approval of the host care organisation must be obtained before recruitment of participants within that host care organisation.

14.5 Sponsorship

The FAKTION trial is being sponsored by Velindre NHS Trust. Velindre NHS Trust shall be responsible for ensuring that the trial is performed in accordance with the following:

- The Medicines for Human Use (Clinical Trials) Regulations 2004 (SI2004/1031) and subsequent amendments
- Conditions and principles of Good Clinical Practice
- Declaration of Helsinki (1996)
- Research Governance Framework for Health and Social Care (Welsh Assembly Government 2009 and Department of Health 2nd July 2005)
- the Data Protection Act 1998
- the Human Tissue Act 2004
- other regulatory requirements as appropriate

The Sponsor has/will be delegating certain responsibilities to [redacted]), the Chief Investigators, Principal Investigators, host sites and other stakeholder organisations as appropriate in accordance with the relevant agreement that is informed by regulation and study type.

14.6 Indemnity

- Non-negligent harm: This trial is an academic, investigator-led and designed trial sponsored by Velindre NHS Trust and coordinated by the WCTU. The Chief Investigator, local Investigators and WCTU do not hold insurance against claims for compensation for injury caused by participation in a clinical trial and therefore cannot offer any non-negligent harm indemnity. The Association of the British Pharmaceutical Industry (ABPI) guidelines will not apply.
- Negligent harm: In accordance with Technical Note 12 Indemnity for Clinical Research for research Sponsored by a Welsh body, Welsh Risk Pool Services provides indemnity cover against successful negligence claims arising from the management and conduct of the study. Where NHS employees are responsible for the design of a study, indemnity cover will also be provided for negligent harm arising from the study design. Velindre NHS Trust does not accept liability for any breach in the other NHS Organisations duty of care, or any negligence on the part of employees of these NHS Organisations.

14.7 Data protection

The WCTU will act to preserve patient confidentiality and will not disclose or reproduce any information by which participants could be identified, except where specific consent is obtained. This includes collection of NHS number (or equivalent – e.g. CHI number in Scotland), name and postcode to register and trace participants with the NHSIC. This also includes collection of NHS number or equivalent to utilise NHS data for future research through Cancer Research UK.

Data will be stored in a secure manner and will be registered in accordance with the Data Protection Act 1998. The data custodian and the translational sample custodian for this trial is the Director of the WCTU.

14.8 Finance

FAKTION is funded by Cancer Research UK (CR-UK) Feasibility Study Committee (FSC). The trial is supported and funded by an Investigator-Sponsored Study Collaboration between AstraZeneca and the National Cancer Research Network. The WCTU is core funded by CR-UK and these core resources will be used to support this trial. The trial is in the National Cancer Research Network (NCRN) and National Institute for Health (NIHR) portfolio. Local NCRN/WCTN/SCRN support should be available at each centre taking part to support entry of participants into this trial.

15.0 References

Altomere DA and Testa JR. Perturbations of the AKT signalling pathway in human cancer. Oncogene 2005; 24:7455-7464.

Bachelot T, Bourgier C, Cropet C, Ray-Coquard I, Ferrero JM, Freyer G, Abadie-Lacourtoisie S, Eymard JC, Debled M, Spaëth D, Legouffe E, Allouache D, El Kouri C, Pujade-Lauraine E. Randomized phase II trial of everolimus in combination with tamoxifen in patients with hormone receptor-positive, human epidermal growth factor receptor 2-negative metastatic breast cancer with prior exposure to aromatase inhibitors: a GINECO study. J Clin Oncol. 2012;30:2718-24.

Balko JM et al Genomic alterations indicative of a luminal A subtype associate with clinical benefit to buparlisib and letrozole in endocrine-resistant ER+/HER2– metastatic breast cancer SABCS 2015 P3-07-41.

Banerji U, Dean E, Pérez-Fidalgo JA, Batist G, Bedard PL, You B, Westin SN, Kabos P, Garrett MD, Tall M, Ambrose HJ, Barrett JC, Carr TH, Cheung SA, Corcoran C, Cullberg M, Davies BR, de Bruin EC, Elvin P, Foxley A, Lawrence P, Lindemann JPO, Maudsley R, Pass M, Rowlands V, Rugman P, Schiavon G, Yates JW, Schellens JHM A Phase 1 open-label study to identify a dosing regimen of the pan-AKT inhibitor AZD5363 for evaluation in solid tumors and in PIK3CA-mutated breast and gynecologic cancers. Clin Cancer Res. 2017 Oct 24. DOI: 10.1158/1078-0432.CCR-17-2260

Baselga J et al PIK3CA status in circulating tumor DNA (ctDNA) predicts efficacy of buparlisib (BUP) plus fulvestrant (FULV) in postmenopausal women with endocrine-resistant HR+/HER2– advanced breast cancer (BC): First results from the randomized, phase III BELLE-2 trial. SABCS 2015 [S6-01].

Blackwell K et al. Phase I/II dose-escalation study of PI3K inhibitors pilaralisib or voxtalisib in combination with letrozole in patients with hormone-receptor-positive and HER2-negative metastatic breast cancer refractory to a non-steroidal aromatase inhibitor. Breast Cancer Research and Treatment 2015; 154:287–297.

Chia S, Gradishar W, Mauriac L, Bines J, Amant F, Federico M, Fein L, Romieu G, Buzdar A, Robertson JF, Brufsky A, Possinger K, Rennie P, Sapunar F, Lowe E, Piccart M. Double-blind, randomized placebo controlled trial of fulvestrant compared with exemestane after prior nonsteroidal aromatase inhibitor therapy in postmenopausal women with hormone receptor-positive, advanced breast cancer: results from EFECT. J Clin Oncol. 2008;26:1664-70.

Davies C, Godwin J, Gray R, Clarke M, Cutter D, Darby S, McGale P, Pan HC, Taylor C, Wang YC, Dowsett M, Ingle J, Peto R. Relevance of breast cancer hormone receptors and other factors to the efficacy of adjuvant tamoxifen: patient-level meta-analysis of randomised trials. Early Breast Cancer Trialists' Collaborative Group (EBCTCG), Lancet. 2011; 378:771-84

Di Leo A, Jerusalem G, Petruzelka L, Torres R, Bondarenko IN, Khasanov R, Verhoeven D, Pedrini JL, Smirnova I, Lichinitser MR, Pendergrass K, Garnett S, Lindemann JP, Sapunar F, Martin M.
Results of the CONFIRM phase III trial comparing fulvestrant 250 mg with fulvestrant 500 mg in postmenopausal women with estrogen receptor-positive advanced breast cancer. J Clin Oncol. 2010;28:4594-600.

Di Nicolantonio F et al Deregulation of the PI3K and KRAS signaling pathways in human cancer cells determines their response to everolimus. J Clin Invest. 2010;120:2858-66.

Ellis MJ, Ding L, Shen D, Luo J, Suman VJ, Wallis JW, Van Tine BA, Hoog J, Goiffon RJ, Goldstein TC, Ng S, Lin L, Crowder R, Snider J, Ballman K, Weber J, Chen K, Koboldt DC, Kandoth C, Schierding WS, McMichael JF, Miller CA, Lu C, Harris CC, McLellan MD, Wendl MC, DeSchryver K, Allred DC, Esserman L, Unzeitig G, Margenthaler J, Babiera GV, Marcom PK, Guenther JM, Leitch M, Hunt K, Olson J, Tao Y, Maher CA, Fulton LL, Fulton RS, Harrison M, Oberkfell B, Du F, Demeter R, Vickery TL, Elhammali A, Piwnica-Worms H, McDonald S, Watson M, Dooling DJ, Ota D, Chang LW, Bose R, Ley TJ, Piwnica-Worms D, Stuart JM, Wilson RK, Mardis ER. Whole-genome analysis informs breast cancer response to aromatase inhibition. Nature 2012;486:353-60.

Fox EM, Kuba MG, Miller TW, Davies BR, Arteaga CL. Autocrine IGF-I/insulin receptor axis compensates for inhibition of AKT in ER-positive breast cancer cells with resistance to estrogen deprivation. Breast Cancer Res. 2013;15(4):R55.

Hyman DM, Smyth LM, Donoghue MTA, Westin SN, Bedard PL, Dean EJ, Bando H, El-Khoueiry AB, Pérez-Fidalgo JA, Mita A, Schellens JHM, Chang MT, Reichel JB, Bouvier N, Selcuklu SD, Soumerai TE, Torrisi J, Erinjeri JP, Ambrose H, Barrett JC, Dougherty B, Foxley A, Lindemann JPO, McEwen R, Pass M, Schiavon G, Berger MF, Chandarlapaty S, Solit DB, Banerji U, Baselga J, Taylor BS. AKT Inhibition in Solid Tumors With AKT1 Mutations. J Clin Oncol. 2017 Jul 10; 35(20):2251-2259.

http://www.cancerresearchuk.org/cancer-info/cancerstats/types/breast/ accessed 23rd April 2013

Hortobagyi GN et al Correlative Analysis of Genetic Alterations and Everolimus Benefit in Hormone Receptor-Positive, Human Epidermal Growth Factor Receptor 2-Negative Advanced Breast Cancer: Results From BOLERO-2. J Clin Oncol. 2016;34:419-26.

Krop I et al. Pictilisib for oestrogen receptor-positive, aromatase inhibitor-resistant, advanced or metastatic breast cancer (FERGI): a randomised, double-blind, placebo-controlled, phase 2 trial. Lancet Oncol. 2016; 17:811-21.

Lindsley CW. The Akt/PKB family of protein kinases: A review of small molecule inhibitors and progress towards target validation: A 2009 update. Current Topics in Medicinal Chemistry 2010;10:458-477.

Ma CX et al A phase II neoadjuvant trial of MK-2206, an AKT inhibitor, in combination with anastrozole for clinical stage 2 or 3 PIK3CA mutant estrogen receptor positive HER2 negative (ER+HER2-) breast cancer. SABCS 2015 [P5-13-04].

Mauri D, Pavlidis N, Polyzos NP, Ioannidis JP. Survival with aromatase inhibitors and inactivators versus standard hormonal therapy in advanced breast cancer: meta-analysis. J Natl Cancer Inst. 2006;98:1285-91.

Mayer IA, Abramson VG, Isakoff SJ, Forero A, Balko JM, Kuba MG, Sanders ME, Yap JT, Van den Abbeele AD, Li Y, Cantley LC, Winer E, Arteaga CL. Stand up to cancer phase Ib study of pan-phosphoinositide-3-kinase inhibitor buparlisib with letrozole in estrogen receptor-positive/human epidermal growth factor receptor 2-negative metastatic breast cancer. J Clin Oncol 2014; 32: 1202-09.

Miller TW, Balko JM, Fox EM, Ghazoui Z, Dunbier A, Anderson H, Dowsett M, Jiang A, Smith RA, Maira SM, Manning HC, González-Angulo AM, Mills GB, Higham C, Chanthaphaychith S, Kuba MG, Miller WR, Shyr Y, Osborne CK, Schiff R. Mechanisms of endocrine resistance in breast cancer. Annu Rev Med. 2011;62:233-47.

Moynahan ME et al. Correlation of PIK3CA mutations in cell-free DNA (cfDNA) and efficacy of everolimus (EVE) in metastatic breast cancer: Results from BOLERO-2. JCO 34; 2016; suppl abstr 519.

O'Brien C et al 2010 Predictive biomarkers of sensitivity to the phosphatidylinositol 3′ kinase inhibitor GDC-0941 in breast cancer preclinical models Clin Cancer Res 16:3670–3683.

Robertson JF, Lindemann JP, Llombart-Cussac A, Rolski J, Feltl D, Dewar J, Emerson L, Dean A, Ellis MJ. Fulvestrant 500 mg versus anastrozole 1 mg for the first-line treatment of advanced breast cancer: follow-up analysis from the randomized 'FIRST' study. Breast Cancer Res Treat. 2012; 136:503-11.

Robertson JFR, Bondarenko IM, Trishkina E, Dvorkin M, Panasci L, Manikhas A, Shparyk Y, Cardona-Huerta S, Cheung KL, Philco-Salas MJ, Ruiz-Borrego M, Shao Z, Noguchi S, Rowbottom J, Stuart M, Grinsted LM, Fazal M, Ellis MJ. Fulvestrant 500 mg versus anastrozole 1 mg for hormone receptor-positive advanced breast cancer (FALCON): an international, randomised, double-blind, phase 3 trial. Lancet. 2016 Dec 17;388 (10063): 2997-3005.

Saal LH, Holm K, Maurer M, Memeo L, Su T, Wang X, Yu JS, Malmström PO, Mansukhani M, Enoksson J, Hibshoosh H, Borg A, Parsons R. PIK3CA mutations correlate with hormone receptors, node metastasis, and ERBB2, and are mutually exclusive with PTEN loss in human breast carcinoma. Cancer Res. 2005 Apr 1;65(7):2554-9.

Sabnis G, Goloubeva O, Jelovac D, Schayowitz A, Brodie A. Inhibition of the phosphatidylinositol 3-kinase/Akt pathway improves response of long-term estrogen-deprived breast cancer xenografts to antiestrogens. Clin Cancer Res. 2007;13:2751-7.

Sanchez CG et al Preclinical modelling of combined phosphatidylinositol-3-kinase inhibition with endocrine therapy for estrogen receptor-positive breast cancer. Breast Cancer Res. 2011 13(2):R21. doi: 10.1186/bcr2833.

Sangai T, Akcakanat A, Chen H, Tarco E, Wu Y, Do KA, Miller TW, Arteaga CL, Mills GB, Gonzalez-Angulo AM, Meric-Bernstam F. Biomarkers of response to Akt inhibitor MK-2206 in breast cancer. Clin Cancer Res. 2012;18:5816-28.

Schmid P et al. Phase II Randomized Preoperative Window-of-Opportunity Study of the PI3K Inhibitor Pictilisib Plus Anastrozole Compared With Anastrozole Alone in Patients With Estrogen Receptor-Positive Breast Cancer. J Clin Oncol. 2016;34:1987-94.

Serra V et al NVP-BEZ235, a dual PI3K/mTOR inhibitor, prevents PI3K signaling and inhibits the growth of cancer cells with activating PI3K mutations. Cancer Res 2008; 68:8022-30.

Sheri A, Martin LA, Johnston S. Targeting endocrine resistance: is there a role for mTOR inhibition? Clin Breast Cancer. 2010;10:S79-85.

Smyth LM, Oliveira M, Ciruelos E, Tamura K, El-Khoueiry A, Mita A et al. AZD5363 in combination with fulvestrant in AKT1-mutant ER-positive metastatic breast cancer. SABCS 2017, P5-21-32/

Stemke-Hale K, Gonzalez-Angulo AM, Lluch A, Neve RM, Kuo WL, Davies M, Carey M, Hu Z, Guan Y, Sahin A, Symmans WF, Pusztai L, Nolden LK, Horlings H, Berns K, Hung MC, van de Vijver MJ, Valero V, Gray JW,

Bernards R, Mills GB, Hennessy BT. An integrative genomic and proteomic analysis of PIK3CA, PTEN, and AKT mutations in breast cancer. Cancer Res. 2008 Aug 1;68(15):6084-91.

Tamura K et al. Safety and tolerability of AZD5363 in Japanese patients with advanced solid tumors. Cancer Chemother Pharmacol. 2016; 77: 787–795.

Turner N, Alarcón E, Armstrong A, Philco M, Lópex Chuken YA, Sablin M, Tamura K, Gomez Villanueva A, Pérez-Fidalgo JA, Foxley A, Lindemann J, Maudsley R, Outhwaite E, Pass M, Rugman P, Schiavon G, Oliveira M. BEECH: A randomised Phase 2 study assessing the efficacy of AKT inhibitor AZD5363 combined with paclitaxel in patients with ER+ve advanced or metastatic breast cancer, and in a PIK3CA mutant sub-population. Ann Oncol 2017; 28(5): v74-v108.

Appendix 1: RECIST criteria

INTRODUCTION

This appendix details the implementation of RECIST 1.1 Guidelines (www.recist.com) for the FAKTION study with regards to Investigator assessment of tumour burden including protocol-specific requirements for this study.

DEFINITION OF MEASURABLE, NON-MEASURABLE, TARGET AND NON-TARGET LESIONS

Measurable:

A lesion, not previously irradiated, that can be accurately measured at baseline as ≥10 mm in the longest diameter (except lymph nodes which must have short axis ≥15 mm) with CT or MRI and which is suitable for accurate repeated measurements.

Non-measurable:

- All other lesions, including small lesions (longest diameter <10 mm or pathological lymph nodes with ≥10 to <15 mm short axis at baseline*).
- Truly non-measurable lesions include the following: bone lesions, leptomeningeal disease, ascites, pleural / pericardial effusion, inflammatory breast disease, lymphangitic involvement of skin or lung, abdominal masses/abdominal organomegaly identified by physical examination that is not measurable by CT or MRI.
- Previously irradiated lesions.**
- Skin lesions assessed by clinical examination.***
- Brain metastasis.***

* Nodes with <10 mm short axis are considered non-pathological and should not be recorded or followed as NTL.

** Localised post-radiation changes which affect lesion sizes may occur. Therefore, lesions that have been previously irradiated will not be considered measurable and must be selected as Non-Target Lesions (NTL) at baseline and followed up as part of the NTL assessment.

*** Skin lesions assessed by clinical examination and brain lesions are considered as NTL.

Special Cases:

- Lytic bone lesions or mixed lytic–blastic lesions, with identifiable soft tissue components, can be considered measurable if the soft tissue component meets the definition of measurability. Blastic lesions are considered non-measurable.
- Cystic metastases can be considered measurable lesions if they meet the criteria for measurability from a radiological point of view, but if non-cystic lesions are present in the same patient; these should be selected as target lesions.

Target lesions:

A maximum of 5 measurable lesions (with a maximum of 2 lesions per organ), representative of all lesions involved suitable for accurate repeated measurement, should be identified as target lesions (TL) at baseline.

Non-Target lesions:

All other lesions (or sites of disease) not recorded as TL should be identified as NTL at baseline.

METHODS OF ASSESSMENT

The same method of assessment and the same technique should be used to characterize each identified and recorded lesion at baseline and during follow-up visits.

A summary of the methods to be used for RECIST assessment is provided below and those excluded from tumour assessments for this study are highlighted, with the rationale provided.

Table 2: Summary of Methods of Assessment

Target Lesions	Non-Target Lesions	New Lesions
CT (preferred)	CT (preferred)	CT (preferred)
MRI	MRI	MRI
	Clinical examination	Clinical examination
	X-ray, Chest x-ray	X-ray, Chest x-ray
		Ultrasound
		Bone Scan
		FDG-PET

1.1 CT and MRI

CT and MRI are generally considered to be the best currently available and reproducible methods to measure TL selected for response assessment and to assess NTL and identification of any new lesions.

1.2 Clinical examination

In the FAKTION study, clinical examination will not be used for assessment of TL. Clinically detected lesions can be selected as target lesions if they are assessed by CT or MRI scans. Clinical examination can be used to assess NTL and to identify the presence of new lesions.

1.3 X-ray

1.3.1 Chest X-ray

Chest x-ray assessment will not be used for assessment of TL as they will be assessed by CT examination or MRI examination. Chest X-ray can, however, be used to assess NTL and to identify the presence of new lesions.

1.3.2 Plain X-ray

Plain x-ray may be used as a method of assessment for bone NTL and to identify the presence of new bone lesions.

1.4 Ultrasound

Ultrasound examination will not be used for assessment of TL and NTL as it is not a reproducible method, does not provide an accurate assessment of tumour size and it is subjective and operator dependent. Ultrasound examination can, however, be used to identify the presence of new lesions. If new clinical symptoms occur and an ultrasound is performed then new lesions should be confirmed by CT or MRI examination.

1.5 Endoscopy and laparoscopy

Endoscopy and laparoscopy will not be used for tumour assessments as they are not validated in the context of tumour assessment.

1.6 Tumour markers

Tumour markers will not be used for tumour response assessments as per RECIST 1.1.

1.7 Cytology and histology

Histology will not be used as part of the tumour response assessment as per RECIST 1.1.

Cytological confirmation of the neoplastic origin of any effusion that appears or worsens during treatment is required when the measurable tumour has met criteria for response or stable disease. In such circumstances, the cytology is necessary to differentiate between response / stable disease (an effusion may be a side effect of the treatment) and progressive disease (if the neoplastic origin of the fluid is confirmed). Where cytology findings are not available, any effusion that significantly worsens (from trace to large) or appearance of clinically significant effusion (requiring change in drug therapy) during the study treatment will be considered to be progression of NTL, or disease progression due to new lesions.

1.8 Isotopic bone scan

Isotopic bone scans are not a protocol requirement for FAKTION, but clinicians may use these if necessary for clinical decision making.

Bone lesions identified on an isotopic bone scan at baseline and confirmed by CT, MRI or X-ray at baseline should be recorded as NTL and followed by the same method as per baseline assessment.

Isotopic bone scans may be used as a method of assessment to identify the presence of new bone lesions at follow-up visits. New lesions will be recorded where a positive hot-spot that was not present on the baseline bone scan assessment is identified on a bone scan performed at any time during the study. The Investigator should consider the positive hot-spot to be a significant new site of malignant disease and represent true disease progression in order to record the new lesion. Confirmation by CT, MRI and x-ray is recommended where bone scan findings are equivocal.

1.9 FDG-PET scan

FDG-PET scans are not a protocol requirement for FAKTION, but clinicians may use these if necessary for clinical decision making.

FDG-PET scans may be used as a method for identifying new lesions, according with the following algorithm: New lesions will be recorded where there is positive FDG uptake* not present on baseline FDG-PET scan or in a location corresponding to a new lesion on CT/MRI at the same follow-up visit. If there is no baseline FDG-PET scan available, and no evidence of new lesions on CT/MRI scans then follow-up CT/MRI assessments should be continued, scheduled as per protocol or clinical indicated, in order to confirm new lesions.

* A positive FDG-PET scan lesion should be reported only when an uptake greater than twice that of the surrounding tissue is observed.

TUMOUR RESPONSE EVALUATION

1.10 Schedule of evaluation

Baseline assessments should encompass all areas of known predilection for metastases in the disease under evaluation and should additionally investigate areas that may be involved based on signs and symptoms of individual patients and should be performed no more than 28 days before the start of study treatment. Follow-up assessments will be performed every 12 weeks ($\pm$7 days) after start of treatment until objective disease progression as defined by RECIST 1.1. Any other sites at which new disease is suspected should also be adequately imaged at follow-up.

If an unscheduled assessment was performed and the patient has not progressed, every attempt should be made to perform the subsequent assessments at their scheduled visits. This schedule is to be followed in order to minimise any unintentional bias caused by some patients being assessed at a different frequency than other patients.

1.11 Target lesions (TL)

1.11.1 Documentation of target lesions

A maximum of 5 measurable lesions, with a maximum of 2 lesions per organ (including lymph nodes), representative of all lesions involved should be identified as TL at baseline. Target lesions should be selected on the basis of their size (longest diameter for non-nodal lesions or short axis for nodal lesions), but in addition should be those that lend themselves to reproducible repeated measurements. It may be the case that, on occasion, the largest lesion does not lend itself to reproducible measurement in which circumstance the next largest lesion, which can be measured reproducibly, should be selected.

The site and location of each TL should be documented as well as the longest diameter for non-nodal lesions (or short axis for lymph nodes). All measurements should be recorded in millimeters. At baseline the sum of the diameters for all TL will be calculated and reported as the baseline sum of diameters. At follow-up visits the sum of diameters for all TL will be calculated and reported as the follow-up sum of diameters.

Special cases:

- For TL measurable in 2 or 3 dimensions, always report the longest diameter. For pathological lymph nodes measurable in 2 or 3 dimensions, always report the short axis.
- If the CT/MRI slice thickness used is >5 mm, the minimum size of measurable disease at baseline should be twice the slice thickness of the baseline scan.
- If a lesion has completely disappeared, the longest diameter should be recorded as 0 mm.
- If a TL splits into two or more parts, then record the sum of the diameters of those parts.
- If two or more TL merge then the sum of the diameters of the combined lesion should be recorded for one of the lesions and 0 mm recorded for the other lesion(s).

- If a TL is believed to be present and is faintly seen but too small to measure, a default value of 5mm should be assigned. If an accurate measure can be given, this should be recorded, even if it is below 5 mm.

- If a TL cannot be measured accurately due to it being too large, provide an estimate of the size of the lesion.

- When a TL has had any intervention e.g. radiotherapy, embolisation, surgery etc., during the study, the size of the TL should still be provided where possible.

1.11.2 Evaluation of target lesions

This section provides the definitions of the criteria used to determine objective tumour visit response for TL.

Table 3: Evaluation of target lesions

Complete Response (CR)	Disappearance of all target lesions since baseline. Any pathological lymph nodes selected as target lesions must have a reduction in short axis to <10 mm.
Partial Response (PR)	At least a 30% decrease in the sum of the diameters of TL, taking as reference the baseline sum of diameters
Stable Disease (SD)	Neither sufficient shrinkage to qualify for PR nor sufficient increase to qualify for PD
Progressive Disease (PD)	At least a 20% increase in the sum of diameters of target lesions, taking as reference the smallest sum on study (this includes the baseline sum if that is the smallest on study). In addition to the relative increase of 20%, the sum must also demonstrate an absolute increase of at least 5mm.
Not Evaluable (NE)	Only relevant if any of the target lesions were not assessed or not evaluable or had a lesion intervention at this visit. Note: If the sum of diameters meets the progressive disease criteria, progressive disease overrides not evaluable as a target lesion response

1.12 Non-Target lesions (NTL)

1.12.1 Evaluation of non-target lesions

All other lesions (or sites of disease) not recorded as TL should be identified as NTL at baseline. Measurements are not required for these lesions, but their status should be followed at subsequent visits. At each visit an overall assessment of the NTL response should be recorded by the Investigator. This section provides the definitions of the criteria used to determine and record overall response for NTL at the investigational site at each visit.

Table 4: Evaluation of Non-Target Lesions

Complete Response (CR)	Disappearance of all non-target lesions since baseline. All lymph nodes must be non-pathological in size (< 10 mm short axis).
Non CR/Non PD	Persistence of one or more NTL

Progression (PD)	Unequivocal progression of existing non-target lesions. Unequivocal progression may be due to an important progression in one lesion only or in several lesions. In all cases the progression MUST be clinically significant for the physician to consider changing (or stopping) therapy.
Not Evaluable (NE)	Only relevant when one or some of the non-target lesions were not assessed and, in the Investigator's opinion, they are not able to provide an evaluable overall non-target lesion assessment at this visit.<br>Note: For patients without target lesions at baseline, this is relevant if any of the non-target lesions were not assessed at this visit and the progression criteria have not been met.

To achieve 'unequivocal progression' on the basis of non-target lesions, there must be an overall level of substantial worsening in non-target disease such that, even in presence of SD or PR in target lesions, the overall tumour burden has increased sufficiently to merit discontinuation of therapy. A modest 'increase' in the size of one or more non-target lesions is usually not sufficient to qualify for unequivocal progression status.

1.13 New Lesions

Details of any new lesions will also be recorded with the date of assessment. The presence of one or more new lesions is assessed as progression.

A lesion identified at a follow up assessment in an anatomical location that was not scanned at baseline is considered a new lesion and will indicate disease progression.

The finding of a new lesion should be unequivocal: i.e. not attributable to differences in scanning technique, change in imaging modality or findings thought to represent something other than tumour.

If a new lesion is equivocal, for example because of its small size, the treatment and tumour assessments should be continued until the new lesion has been confirmed. If repeat scans confirm there is a new lesion, then the progression date should be declared using the date of the initial scan.

1.14 Symptomatic deterioration

Symptomatic deterioration is not a descriptor of an objective response: it is a reason for stopping study therapy.

Patients with 'symptomatic deterioration' requiring discontinuation of treatment without objective evidence of disease progression at that time should continue to undergo tumour assessments where possible until objective disease progression is observed.

1.15 Evaluation of Overall Visit Response

The overall visit response will be derived using the algorithm shown in Table 5.

Table 5: Overall Visit Response

Target lesions	Non-Target lesions	New Lesions	Overall response
CR	CR	No	CR
CR	NA	No	CR

Target lesions	Non-Target lesions	New Lesions	Overall response
NA	CR	No	CR
CR	Non CR/Non PD	No	PR
CR	NE	No	PR
PR	Non PD or NE	No	PR
SD	Non PD or NE	No	SD
NA	Non CR/Non PD	No	SD (Non CR/Non PD)
NE	Non PD or NE	No	NE
NA	NE	No	NE
PD	Any	Yes or No	PD
Any	PD	Yes or No	PD
Any	Any	Yes	PD

CR = complete response, PR = partial response, SD = stable disease, PD = progressive disease, NE = not evaluable, NA = not applicable (only relevant if there were no TL/NTLs at baseline).

SPECIFICATIONS FOR RADIOLOGICAL IMAGING

These notes are recommendations for use in clinical studies. The use of standardized protocols for CT and MRI allows comparability both within and between different studies, irrespective of where the examination has been undertaken.

1.16 CT Scan

CT scans of the chest, abdomen, and pelvis should be contiguous throughout all the anatomic region of interest.

The most critical CT image acquisition parameters for optimal tumour evaluation using RECIST 1.1 are anatomic coverage, contrast administration, slice thickness, and reconstruction interval.

a. Anatomic coverage: Optimal anatomic coverage for most solid tumours is the chest, abdomen and pelvis. Coverage should encompass all areas of known predilection for metastases in the disease under evaluation and should additionally investigate areas that may be involved based on signs and symptoms of individual patients. Because a lesion later identified in a body part not scanned at baseline would be considered as a new lesion representing disease progression, careful consideration should be given to the extent of imaging coverage at baseline and at subsequent follow-up time points. This will enable better consistency not only of tumour measurements but also identification of new disease.

b. IV contrast administration: Optimal visualisation and measurement of metastases in solid tumours requires consistent administration (dose and rate) of IV contrast as well as timing of scanning. Typically, most abdominal imaging is performed during the portal venous phase and (optimally) about the same time frame after injection on each examination. An adequate volume of a suitable contrast agent should be given so that the metastases are demonstrated to best effect and a consistent method is used on subsequent examinations for any given patient. It is very important that the same technique be used at baseline and on follow- up examinations for a given patient. For patients who develop contraindications to contrast after baseline contrast CT is done, the decision as to whether non-contrast CT or MRI (enhanced

or non-enhanced) should be performed should also be based on the tumour type, anatomic location of the disease and should be optimised to allow for comparison to the prior studies if possible. Each case should be discussed with the radiologist to determine if substitution of these other approaches is possible and, if not, the patient should be considered not evaluable from that point forward. Care must be taken in measurement of target lesions on a different modality and interpretation of non-target disease or new lesions, since the same lesion may appear to have a different size using a new modality. Oral contrast is recommended to help visualise and differentiate structures in the abdomen.

If iodine contrast media is medically contraindicated at baseline or at any time during the course of the study then the recommended methods are: CT thoracic examination without contrast and abdominal and pelvis MRI with contrast. If MRI cannot be performed then CT without intravenous contrast is an option for the thorax, abdomen and pelvis examination. For brain lesions assessment, MRI is the preferred method.

c. Slice thickness and reconstruction interval: It is recommended that CT scans be performed at 5mm contiguous slice thickness and this guideline presumes a minimum 5 mm thickness in recommendations for measurable lesion definition. Exceptionally, particular institutions may perform medically acceptable scans at slice thicknesses greater than 5 mm. If this occurs, the minimum size of measurable lesions at baseline should be twice the slice thickness of the baseline scans.

All window settings should be included in the assessment, particularly in the thorax where lung and soft tissue windows should be considered. When measuring lesions, the TL should be measured on the same window setting for repeated examinations throughout the study. All images from each examination should be included in the assessment and not “selected” images of the apparent lesion.

1.17 MRI Scan

MRI has excellent contrast, spatial and temporal resolution; however, there are many image acquisition variables involved in MRI, which greatly impact image quality, lesion conspicuity and measurement. Furthermore, the availability of MRI is variable globally. The modality used at follow-up should be the same as was used at baseline and the lesions should be measured/assessed on the same pulse sequence. Generally, axial imaging of the abdomen and pelvis with T1 and T2 weighted imaging along with gadolinium-enhanced imaging should be performed. The field of view, matrix, number of excitations, phase encode steps, use of fat suppression and fast sequences should be optimised for the specific body part being imaged as well as the scanner utilised. It is beyond the scope appendix to prescribe specific MRI pulse sequence parameters for all scanners, body parts and diseases. Ideally, the same type of scanner should be used and the image acquisition protocol should be followed as closely as possible to prior scans. Body scans should be performed with breath-hold scanning techniques if possible.

For these reasons, CT is the imaging modality of choice.

1.18 FDG-PET scans

FDG-PET has gained acceptance as a valuable tool for detecting, staging and restaging several malignancies. If FDG-PET scans are included in a protocol, an FDG uptake period of 60 min prior to imaging has been decided as the most appropriate for imaging of patients with malignancy. Whole-body acquisition is important since this allows for sampling of all areas of interest and can assess if new lesions have appeared thus determining the possibility of interval progression of disease. Images from the base of the skull to the level of the mid-thigh should be obtained 60 min post injection. PET camera specifications are variable and manufacturer specific, so every attempt should be made to use the same scanner, or the same model scanner, for serial scans on the same patient. Whole-body acquisitions can be performed in

either 2- or 3-dimensional mode with attenuation correction, but the method chosen should be consistent across all patients and serial scans in the clinical trial.

1.18.1 PET/CT scans

At present, low dose or attenuation correction CT portions of a combined PET–CT are of limited use in anatomically based efficacy assessments and it is therefore suggested that they should not be substituted for dedicated diagnostic contrast enhanced CT scans for tumour measurements by RECIST 1.1. In exceptional situations, if a site can document that the CT performed as part of a PET–CT is of identical diagnostic quality to a diagnostic CT (with IV and oral contrast) then the CT portion of the PET–CT can be used for RECIST measurements. However, this is not recommended because the PET portion of the CT introduces additional data that may bias an investigator if it is not routinely or serially performed.

REFERENCES

Eisenhauer et al 2009
Eisenhauer EA, Therasse P, Bogaerts J, Schwartz LH, Sargent D, Ford R. New response evaluation criteria in solid tumours: Revised RECIST guideline (version 1.1). Eur J Cancer 45 (2009) 228-247

Appendix 2 – Change in Tumour Size

Tumour size is the sum of the longest diameters of the target lesions. Target lesions are measurable tumour lesions. The change in tumour size will be assessed using the log(ratio) of the week 8 tumour size over the baseline tumour size for each subject.
Patients who progress before week 8 should have a tumour assessment at the time of progression prior to treatment discontinuation. The tumour size from the progression assessment will be used instead of the week 8 assessment for these patients.

Missing data for tumour size analysis will be imputed as follows:

The first CT scan should be performed at the week 8 visit, if this is not performed at the scheduled time point then the first CT scan performed after starting trial treatment will be used.

If a patient has an incomplete week 8 assessment then provided ≤ 1/3 of the lesions sizes are missing, the sum of diameters at week 8 can be estimated by applying the scaling up rule described below. This estimated sum of diameters will be used in the analyses to estimate change in tumour size at 8 weeks.

Where baseline target lesion measurements were available, if ≤ 1/3 of the lesion sizes are missing at week 8 then a scaling up rule will be applied as follows:

- If ≤ 1/3 of target lesions recorded at baseline are missing then the results will be scaled up (based on the baseline sizes) to give an estimated sum of diameters and this will be used in calculations (this is equivalent to comparing the visit sum of diameters of the non-missing lesions to the baseline sum of diameters excluding the lesions that are missing and determining at what rate the lesions are changing)
- If > 1/3 of target lesions recorded at baseline are missing then the target lesion response will be NE unless the sum of non-missing target lesion diameters would result in PD, then assume that the change in tumour size is increased by 20%
- If a patient does not undergo a valid week 8 assessment (ie, sum of diameters at week 8 is unknown or cannot be estimated) then the following imputation rules will apply. This includes patients who progress prior to week 8 (± 1 week).

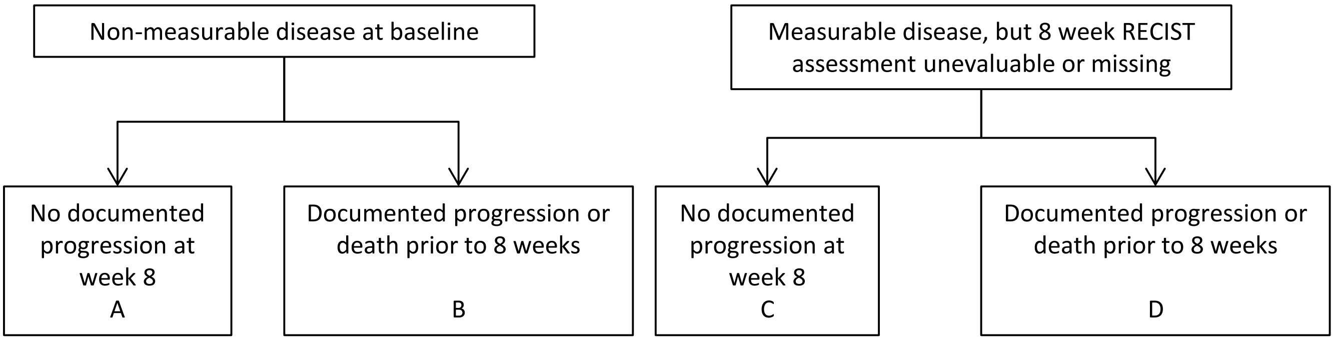

Missing Data Imputation Methods

Strategy	Missing Data Imputation Method
A	Impute change in tumour size is 0%
B	Impute change in tumour size as 20% increase
C	Impute change in tumour size as 0%
D	Impute change in tumour size as 20% increase

The change in tumour size will be assessed in the first 40 wildtype participants, as the log of the ratio of the week 8 over the baseline tumour size measurement for each patient. The effect of AZD5363 on change in tumour size will be estimated from an ANCOVA model adjusted for treatment with baseline tumour size as a covariate. If the Week 8 tumour size is 0, this will be imputed as 0.01 as the log transformed value in the log transformed analysis of these data. The results will be presented in terms of adjusted means (lsmeans) for each treatment together with their 2-sided 80% confidence intervals. Estimates of the treatment effect in all patients (difference in lsmeans, AZD5363– placebo) will be calculated together with their 2-sided 80% confidence interval. These point and interval estimates will be exponentially back transformed to provide estimates of the percentage change from baseline tumour size at 8 weeks. The number of subjects and percentage of subjects in each treatment group whose 8-week data is imputed will be presented on the footnote to the table of output from this analysis.

Appendix 3: List of concomitant medications that should be avoided

TREATMENT WITH COUMARINS SUCH AS WARFARIN IS NOT PERMITTED IN THE STUDY.

CONCOMITANT THERAPIES

Drugs affecting CYP3A4 metabolism that AstraZeneca strongly recommend are not combined with AZD5363

There are currently no data confirming that there are any pharmacokinetic (PK) interactions between any agents and AZD5363. The potential interactions detailed below are considered on the basis of the preclinical data only. The following lists are not intended to be exhaustive, and a similar restriction will apply to other agents that are known to strongly modulate CYP3A4 activity. Appropriate medical judgment is required. Please contact AstraZeneca with any queries you have on this issue.

[redacted]	[redacted]
[redacted]	[redacted]
[redacted]	[redacted]

[redacted]	[redacted]
[redacted]	[redacted]
[redacted]	
[redacted]	
[redacted]	[redacted]

Drugs affecting CYP3A4 metabolism that AstraZeneca considers may be allowed with caution.

[redacted]	[redacted]
[redacted]	[redacted]
[redacted]	[redacted]

Medicines that are significantly metabolised by CYP3A4 that AstraZeneca strongly recommend are not combined with AZD5363

There are currently no data confirming that there are any pharmacokinetic (PK) interactions between AZD5363 and the following CYP3A4 substrates. The potential interactions detailed below are considered on the basis of the preclinical data only. The following list is not intended to be exhaustive, and a similar restriction will apply to other agents that are known to be sensitive to CYP3A4 inhibitors. Appropriate medical judgment is required. Please contact AstraZeneca with any queries you have on this issue.

[redacted]	[redacted]
[redacted]	[redacted]
[redacted]	[redacted]

Specific guidance on concomitant statin use with AZD5363
The CYP3A4 isozyme is responsible for the metabolism of atorvastatin (ATV), cerivastatin (CRV), lovastatin (LOV), and simvastatin (SMV), and their exposure, pharmacological action and toxicity may be increased by inhibition of CYP 3A4. For these statins the potential for CYP-mediated drug-drug interactions (DDIs) with AZD5363 is high. However, there is minimal metabolism of fluvastatin (FLV), pravastatin (PRV), or rosuvastatin (RSV) by CYP3A4 and thus plasma levels are minimally influenced by CYP3A4 inhibitors, conveying a relatively low potential for clinically significant DDIs with AZD5363 via this mechanism.

New and emerging in vitro data has revealed that AZD5363 also has a potential to inhibit the OATP1B1 transporter. This transporter is implicated in the distribution and clearance of many of the statins. Of the statins that are minimally affected by CYP3A4 inhibition, RSV and PRV (but not FLV) can be affected by OATP1B1 inhibition. Based on an in vitro assessment of the potential for AZD5363 to inhibit OATP1B1 the AUC of these statins may be increased by 1.3-fold for PRV and 1.5-fold for RSV (a 'worst-case' assessment of the fold increase).

On this basis, AstraZeneca has modified its advice in relation to co-administration of statins and AZD5363. The initial advice was that if statins were required in a study patient, the agents RSV, PRV and FLV should be preferred. It is now additionally recommended that, if used, doses of RSV be capped to 10 mg once daily, and PRV be capped to 40 mg once daily when combined with AZD5363, and for a 2 week period before and after AZD5363 treatment. No dose cap is required for FLV.

Ref: Michalets EL. Update: clinically significant cytochrome P-450 drug interactions. Pharmacother. 1998;18(1):84-112. http://www.ncbi.nlm.nih.gov/pubmed/9469685

Agents that are significantly metabolised by CYP3A4 that the study team considers may be allowed with caution

[redacted]	[redacted]
[redacted]	[redacted]

Agents that are sensitive to CYP2D6 inhibition that AstraZeneca strongly recommends are not combined with AZD5363

There are currently no data confirming that there are any pharmacokinetic (PK) interactions between AZD5363 and the following CYP2D6 substrates. The potential interactions detailed below are considered on the basis of the preclinical data only. The following list is not intended to be exhaustive, and a similar restriction will apply to other agents that are known to be sensitive to CYP2D6 inhibitors. Appropriate medical judgment is required. Please contact AstraZeneca with any queries you have on this issue.

[redacted]	[redacted]
[redacted]	[redacted]
[redacted]	[redacted]

Agents that are sensitive to CYP2D6 inhibition that AstraZeneca considers may be allowed with caution

[redacted]	[redacted]
[redacted]	[redacted]

Agents that are sensitive to combined CYP3A4 and CYP2D6 inhibition that AstraZeneca strongly recommend are not combined with AZD5363

There are currently no data confirming that there is a pharmacokinetic (PK) interaction between AZD5363 and the following agents; a potential interaction is considered on the basis of the preclinical data only. This list is not intended to be exhaustive, and a similar restriction will apply to other agents with narrow therapeutic windows that are known to depend on combined CYP3A4 and CYP2D6 metabolism. Appropriate medical judgment is required. Please contact AstraZeneca with any queries you have on this issue.

[redacted]	[redacted]
[redacted]	[redacted]
[redacted]	[redacted]

Guidance for drugs that are that are significantly metabolised by CYP2B6, CYP2C9 or CYP2C19 and have a narrow therapeutic margin that AstraZeneca considers may be allowed with caution

Weak signals for competitive inhibition of CYP2B6, CYP2C9 and CYP2C19 cytochrome P450 activities have been demonstrated by in vitro laboratory investigations. There are currently no data confirming that there is a pharmacokinetic (PK) interaction between AZD5363 and substrates of these isoforms; a potential interaction is considered on the basis of the preclinical data only. The following list is intended to identify known sensitive substrates of CYP2B6, CYP 2C9 and CYP 2C19 that have a narrow therapeutic margin. The list is not intended to be exhaustive, and a similar restriction should be applied to any other sensitive substrate with narrow therapeutic margin. Appropriate medical judgment is required. Please contact AstraZeneca with any queries you have on this issue.

[redacted] [redacted]

[redacted] [redacted]

[redacted]

[redacted]

[redacted]

Appendix 4: Management of toxicities

DOSE ADJUSTMENTS FOR TOXICITY

In the event of an AE during the conduct of the FAKTION study which the investigator considers to be related to the administration of study treatment - AZD5363/placebo or fulvestrant - supportive therapy should be given at the discretion of the investigator. In addition, the investigator may decide that dosing of either study treatment should be temporarily interrupted, a subsequent treatment cycle delayed or study treatment permanently discontinued as per the guidelines outlined below.

Please refer to the study protocol sections 7.2: 'Doses delays and modifications' and 7.3: 'Management of toxicities' for general guidance regarding the parameters for study drug dose modification or withdrawal. The following is intended as guidance for investigators regarding dose adjustments for toxicities, with specific reference to management of AZD5363/placebo for hyperglycaemia, diarrhoea and rash.

SUMMARY OF GUIDANCE ON DOSE ADJUSTMENTS OF AZD5363/PLACEBO FOR TOXICITY

If a patient experiences a clinically significant and/or unacceptable toxicity including a DLT not attributable to the disease or disease-related processes under investigation, AZD5363/placebo dosing will be interrupted or the dose reduced and supportive therapy administered as required.

If the toxicity resolves or reverts to ≤ CTCAE grade 1 within 28 days of onset and the patient is showing clinical benefit, treatment with AZD5363 may be restarted at the next lowest dose level (see Figure 4 and section 7.3). If further toxicity develops, a participant may have a further dose reduction. However, a participant may not have more than two dose reductions without prior discussion with the Chief Investigators. If the toxicity does not resolve to ≤ CTCAE grade 1 after 28 days, then the patient should be discontinued from treatment and observed until resolution of the toxicity.

Patients experiencing CTC Grade 4 hyperglycaemia will not be permitted to restart AZD5363/placebo.

If a patient exhibits an aspartate aminotransferase (AST), or alanine aminotransferase (ALT) result in excess of 10x ULN, or AST or ALT in excess of 8x ULN in combination with a doubling of bilirubin from baseline, which is considered to be related to study drug, they will not be permitted to restart AZD5363/placebo.

Hy's Law

Cases where a patient shows an AST or ALT ≥3x ULN or total bilirubin ≥ 2x ULN may need to be reported as SAEs, please refer to Appendix 5 Hy's Law for further instructions.

Figure 4: Toxicity management algorithm

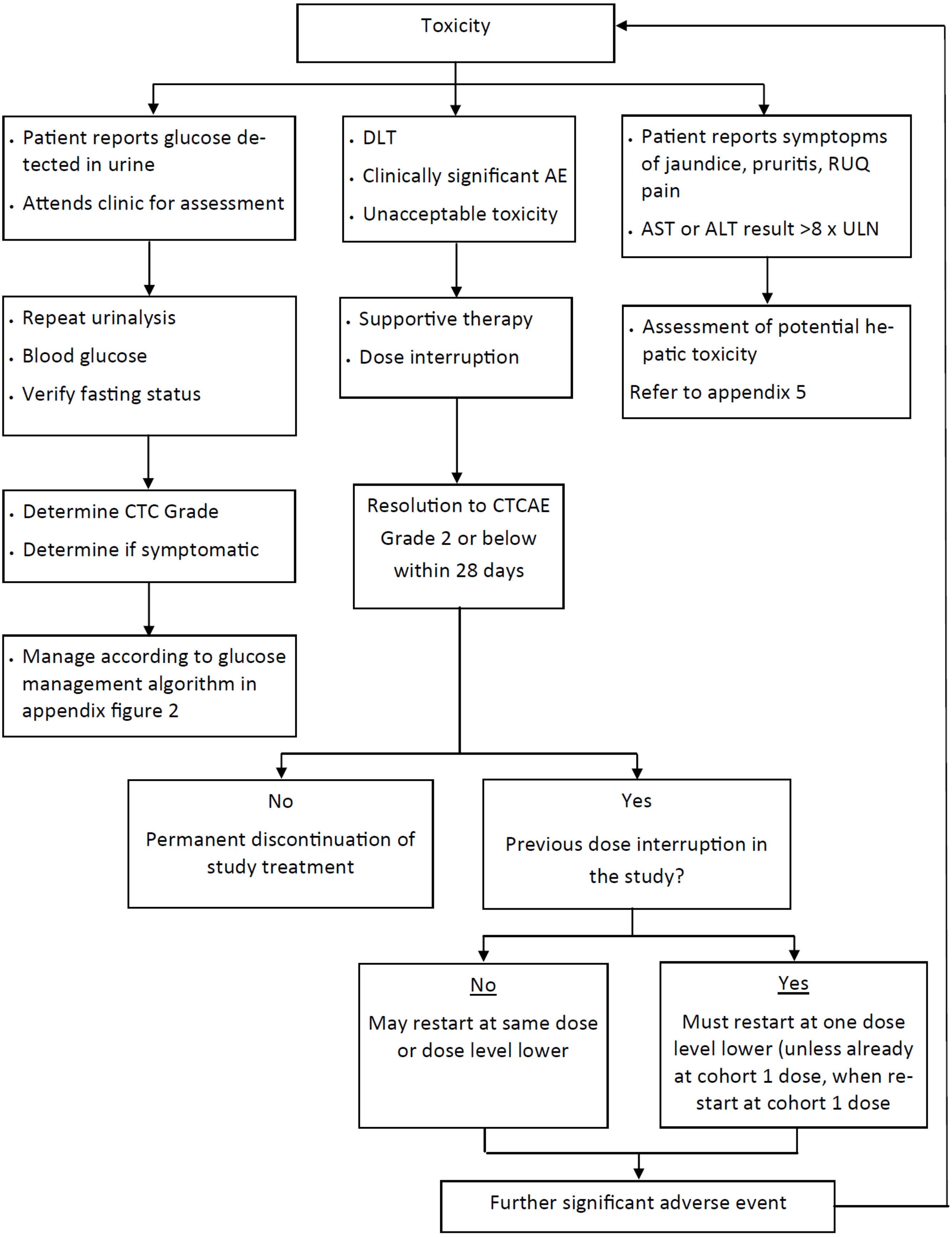

Table 15: Summary of guidance on dose adjustments of AZD5363/placebo for toxicity

Toxicity	AZD5363/placebo
Diarrhoea of CTCAE Grade ≥3 or that is clinically significant or intolerable and causally related to treatment with AZD5363/placebo	Institute appropriate anti-diarrhoeal treatment with loperamide or similar. If clinically appropriate or if toxicity does not improve to CTCAE Grade < 2 or remains clinically intolerable, despite optimal treatment, withhold AZD5363/matching placebo for up to 14 days.<br><br>If toxicity improves to CTCAE Grade <2, becomes clinically tolerable and is considered related to AZD5363/placebo treatment, re-instate AZD5363/placebo at a reduced dose (1 dose level) maintaining anti-diarrhoeal treatment if necessary.<br><br>Where a CTCAE Grade >2 or clinically significant or intolerable toxicity, that is considered related to AZD5363/placebo treatment, does not improve after 14 days of AZD5363/placebo dose interruption, AZD5363/placebo should be permanently discontinued.
Recurrence	On recurrence of CTCAE Grade >2 or clinically significant or intolerable diarrhoea, reinstate anti-diarrhoeal treatment as required. If toxicity does not improve to CTCAE Grade <2 or remains clinically significant or intolerable, despite optimal treatment, withhold dose for up to 14 days until improvement of toxicity.<br><br>If toxicity improves to CTCAE Grade <2, becomes clinically tolerable and is considered related to AZD5363/placebo treatment, re-instate AZD5363/placebo at a reduced dose (1 dose level) maintaining anti-diarrhoeal treatment if necessary. Two dose reductions are permitted without prior discussion with Chief Investigators.
Any skin reaction of CTCAE Grade ≥3 or that is clinically significant or intolerable with **unknown cause**	For any skin reaction that is CTCAE Grade ≥3 or clinically significant or intolerable withhold dose for up to 28 days until improvement of toxicity.
Any skin reaction of CTCAE Grade ≥3 or that is clinically significant or intolerable and **causally related to treatment with AZD5363/placebo**	For any skin reaction that is CTCAE Grade ≥3 or clinically significant or intolerable withhold dose for up to 14 days until improvement of toxicity.<br><br>If toxicity improves to CTCAE Grade ≤2 within 14 days reinstate AZD5363/placebo at a reduced dose (1 dose level) maintaining treatment for toxicity as necessary. If grade ≥3 rash recurs on rechallenge then AZD5363 should be permanently discontinued.<br><br>Where a CTCAE Grade ≥3 or clinically significant or intolerable skin reaction does not improve to a lower CTCAE Grade within 14 days of AZD5363/placebo dose interruption, AZD5363/matching placebo should be permanently discontinued.
Recurrence	NB. Skin reactions so far described with AZD5363, from phase I studies, are idiosyncratic and not dose related. If, despite one dose reduction, a skin reaction recurs that is not manageable with topical therapies such as corticosteroids and emollients, treatment with AZD5363/placebo should be discontinued.
High QTc	If the patient develops symptomatic QTc prolongation or develops ECG evidence of ventricular arrhythmia then AZD5363 should be permanently discontinued and the patient managed according to local cardiac guidelines.<br><br>If mean resting corrected QT interval (QTc) rises to >500 msec or >60 msec over baseline value obtained from 3 consecutive ECGs (taken 1 minute apart) and the patient is asymptomatic then AZD5363 should be temporarily discontinued but fulvestrant continued. Blood electrolytes

	should be checked and normalised as appropriate.<br><br>Triplicate ECGs should be repeated 24 hours later and AZD5363 reinstituted with one dose level reduction only if QTc falls below 480 msec and only if the patient remains asymptomatic. If QTc does not fall below 480 msec at 24 hours the ECGs should be repeated 1 week +/-2 days later for re-evaluation. AZD5363 should only be reinstituted, with one dose level reduction, if QTc falls below 480 msec and only if the patient remains asymptomatic. If the QTc has not fallen below 480 msec then AZD5363 should be permanently discontinued.<br><br>When AZD is reinstituted following reduction of QTc to <480 msec a repeat ECG should be performed at 24 and 72 hours after reinstitution (Days 2 and 4 of the weekly cycle) and on day 1 of each subsequent weekly AZD5363 dosing cycle for an additional 3 weeks. If QTc prolongation >500 msec or >60 msec from baseline recurs then AZD5363 should be permanently discontinued.
Proteinuria	If 3+ proteinuria is identified by dipstick assessment, a 24-hour urine collection for quantification of protein excretion should be performed. If 24 hour urine protein ≥ 3.5g/24hrs (grade 3 CTCAE v.4.03) then AZD5363 should be withheld until proteinuria has recovered to ≤grade1. It is recommended that 24 hour urine collections are performed every 1-2 weeks until recovery and a one level dose reduction is performed when AZD5363 is restarted. Once restarted repeat 24 hour urine collection is only required if proteinuria of 3+ is detected again on urine dipstick.<br><br>If proteinuria is 3+ on three successive occasions with < grade 3 proteinuria by 24 hour urine collection each time and normal serum albumin levels, then urine dipstick of 3+ should not be acted upon for subsequent cycles. However, 24 hour urine collection is recommended at day 1 of each subsequent cycle to ensure proteinuria does not progress.
Hyperglycaemia related to treatment with AZD5363/ placebo<br><br>Please also see the figures overleaf<br><br>Please also see the figures overleaf	1. Grade ≥1 hyperglycaemia detected on fasting blood glucose sample on day 1 of a treatment cycle.<br><br>• Check for symptoms of hyperglycaemia. If hyperglycaemia is symptomatic follow hospital guidelines<br><br>• Confirm fasting status: if not adequately fasted (<8 hours without caloric intake) repeat fasting glucose assessment the following day<br><br>• In the absence of symptoms and with confirmed fasting hyperglycaemia, institute treatment with metformin 500mg bd for the days of treatment with AZD5363/placebo only. Refer to section 7.7 for guidance on the management of metformin therapy.<br><br>• If fasting hyperglycaemia persists despite metformin therapy, reduce AZD5363/placebo one dose level and repeat fasting glucose one week later<br><br>• If fasting hyperglycaemia persists despite metformin therapy and first reduction in AZD5363/placebo dose, proceed to second AZD5363/placebo dose reduction and reassess fasting glucose and creatinine one week later.<br><br>• If fasting hyperglycaemia persists despite metformin therapy and two dose reductions of AZD5363/placebo, discontinue therapy with AZD5363/placebo.<br><br>• If fasting hyperglycaemia resolves with metformin or dose reduction participant should continue on treatment but must have fasting glucose assessed on day one of at least the next 2 treatment cycles to re-evaluate glycaemic control.<br><br>2. Glucose self-detected by participant on urine-dipstick<br><br>• Assess for symptoms of hyperglycaemia and review that day if symptoms present, following hospital guidelines for management.

	• If asymptomatic, review the following day for fasting glucose analysis (prior to taking AZD5363/placebo if on treatment days).<br>o In patients currently taking AZD5363/placebo (4 days on): if fasting hyerglycaemia is grade 1 continue AZD5363/placebo with no dose modification. If fasting hyperglycaemia is grade ≥2 commence metformin 500mg bd during days of AZD5363/placebo treatment and monitor fasting glucose and creatinine according to the guidelines above.<br>o In patients not taking AZD5363/placebo (3 days off) and fasting blood glucose drawn >24 hours after the last dose of AZD5363/placebo is elevated (grade ≥1 hyperglycaemia), hospital guidelines should be followed for the management of hyperglycaemia and metformin should be commenced with next dose of AZD5363.

Figure 5: Glucose management algorithm

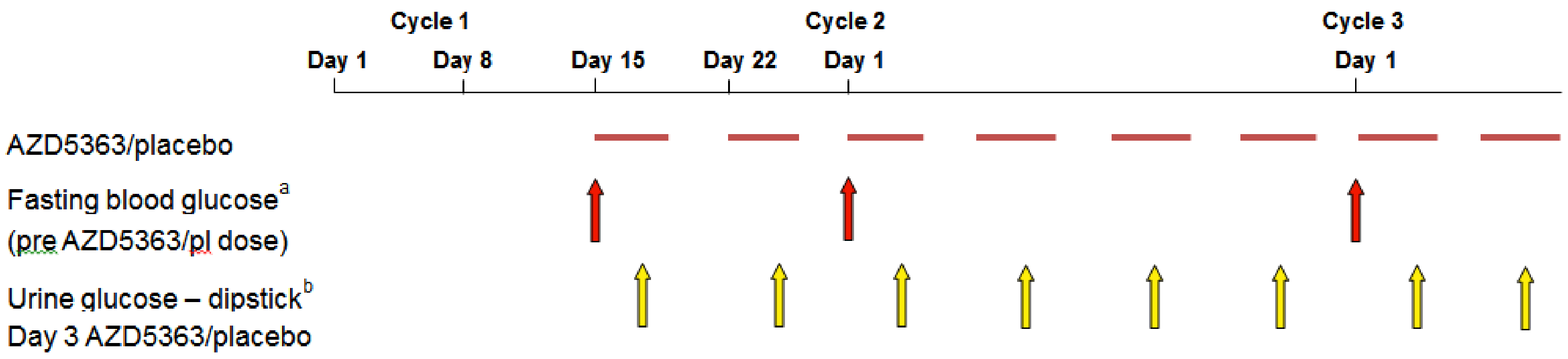

[a] If any grade of hyperglycaemia is seen on a confirmed fasting blood glucose pre-dosing before the 4 day block of AZD5363/placebo treatment, metformin should be commenced in accordance with the metformin guidelines (section 7.7 and appendix 1 table 1) and continued for the duration of AZD5363/placebo therapy.

[b] if gycosuria is detected on day 3 of AZD5363/placebo treatment the patient will contact their trial nurse and arrange for a fasting blood glucose to be performed the following day and before AZD5363/placebo dose is taken.

- If any grade of symptomatic hyperglycaemia or grade >1 fasting hyperglycaemia in the absence of symptoms is present during treatment days with AZD5363/placebo (or the morning of day 5) then metformin should be commenced in accordance with the metformin guidelines (section 7.7 and appendix 1 table 1) and continued for the duration of AZD5363/placebo therapy in the study
- If ≤ grade 1 fasting hyperglycaemia is seen in the absence of symptoms of hyperglycaemia, AZD5363/placebo can be continued without metformin treatment if subsequent glucose assessments on D1 of each cycle are in the normal range.
- If two positive urine dipsticks have been followed up with demonstration of fasting blood glucose ≤grade 1 in the absence of symptoms, no further on-treatment fasting blood glucose assessments will be required.

Figure 6: Non-fasting blood glucose management algorithm

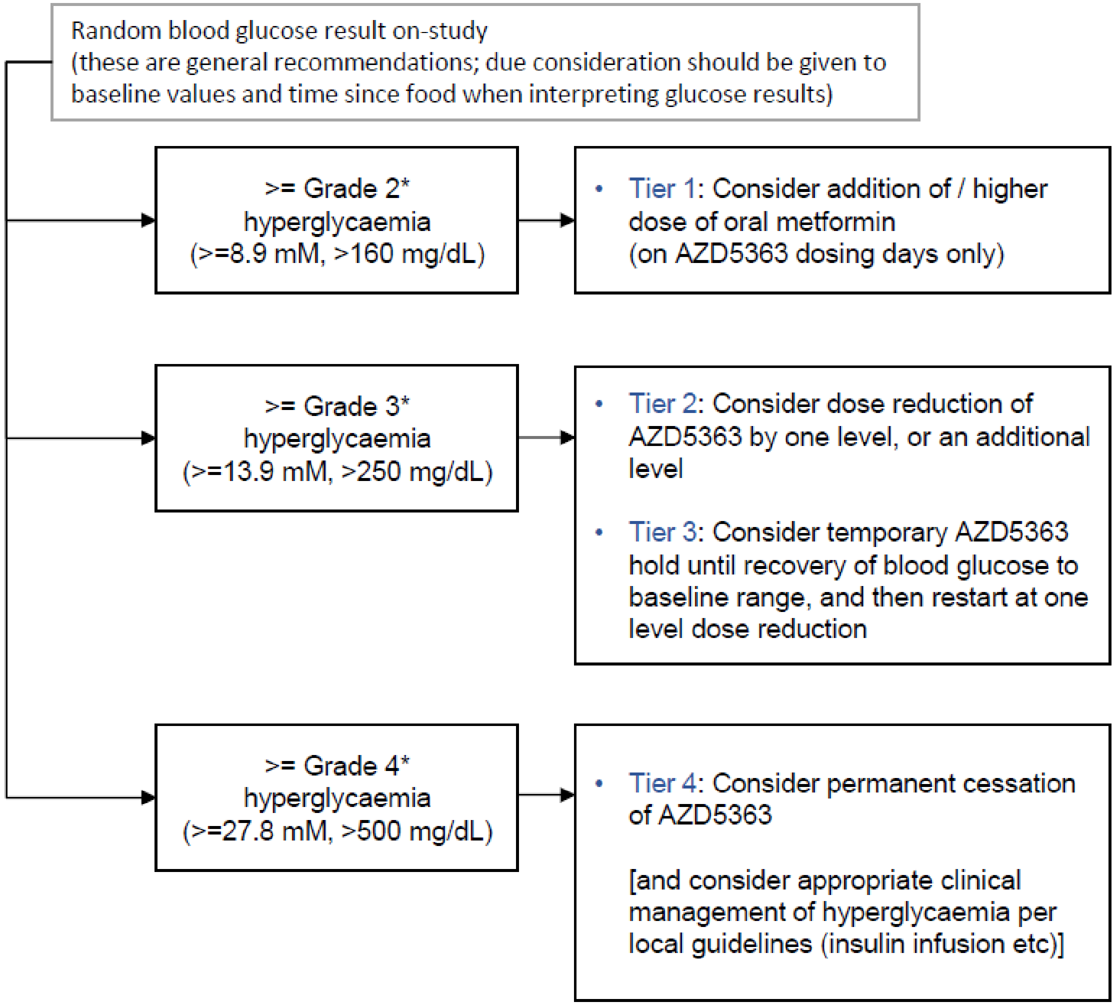

Where a patients exhibits signs or reports symptoms of hyperglycaemia (rather than biochemical findings in isolation of signs / symptoms) the investigator should consider actions at a "+1" tier in this guidance schema, e.g. where a Grade 3 biochemical hyperglycaemia is associated with a report of polydipsia and polyuria, consideration should be given to the "Tier 4" advice.

Symptoms of hyperglycaemia include polyphagia, polydipsia and polyuria.

It is recommended that approaches to the management of AZD5363-induced hyperglycaemia in diabetic patients include advice from the patient's diabetologist where appropriate.

*These grade thresholds based on CTCAE cut-offs for fasting glucose, but applied to random glucose here.

Appendix 5: Hy's Law

1. INTRODUCTION

During the course of the study the Investigator will remain vigilant for increases in liver biochemistry. The investigator is responsible for determining whether a patient meets potential Hy's Law (PHL) criteria at any point during the study. The Investigator participates, together with the study team, in review and assessment of cases meeting PHL criteria to agree whether Hy's Law (HL) criteria are met. HL criteria are met if there is no alternative explanation for the elevations in liver biochemistry other than Drug Induced Liver Injury (DILI) caused by the Investigational Medicinal Product (IMP).
The Investigator is responsible for recording data pertaining to PHL/HL cases and for reporting Adverse Events (AE) and Serious Adverse Events (SAE) according to the outcome of the review and assessment in line with standard safety reporting processes.

2. DEFINITIONS

Potential Hy's Law (PHL)

Aspartate Aminotransferase (AST) or Alanine Aminotransferase (ALT) ≥ 3x Upper Limit of Normal (ULN) **and** Total Bilirubin (TBL) ≥ 2xULN at any point during the study irrespective of an increase in Alkaline Phosphatase (ALP). The elevations do not have to occur at the same time or within a specified time frame.

Hy's Law (HL)

AST or ALT ≥ 3x ULN and TBL ≥ 2xULN, where no other reason, other than the IMP, can be found to explain the combination of increases, eg, elevated ALP indicating cholestasis, viral hepatitis, another drug. The elevations do not have to occur at the same time or within a specified time frame.

3. IDENTIFICATION OF POTENTIAL HY'S LAW CASES

In order to identify cases of PHL it is important to perform a comprehensive review of laboratory data for any patient who meets any of the following identification criteria in isolation or in combination:

- ALT ≥3x ULN
- AST ≥3x ULN
- TBL ≥2x ULN

The Investigator will, without delay, review each new laboratory report and if the identification criteria are met will:

- Determine whether the patient meets PHL criteria (see Section 2 of this Appendix for definition) by reviewing laboratory reports from all previous visits
- Promptly enter the laboratory data into the SAE form and submit as an SAE to WCTU – see section 9.0 of main Protocol - Safety reporting and pharmacovigilance

4. FOLLOW-UP

4.1 Potential Hy's Law Criteria not met

If the patient does not meet PHL criteria the Investigator will:

- Perform follow-up on subsequent laboratory results according to the guidance provided in the Clinical Study Protocol.

4.2 Potential Hy's Law Criteria met

If the patient does meet PHL criteria the Investigator will:

- Determine whether PHL criteria were met at any study visit prior to starting study treatment in the presence of liver metastases (See Section 6)
- On receipt of the SAE form at WCTU the safety team will provide guidance to the investigator, discuss and agree an approach for the study patients' follow-up and the continuous review of data, according to the trial specific SAE management SOP. Subsequent to this contact the Investigator will:
  - Monitor the patient until liver biochemistry parameters and appropriate clinical symptoms and signs return to normal or baseline levels, or as long as medically indicated
  - Investigate the etiology of the event and perform diagnostic investigations as discussed with the Study Physician

5. REVIEW AND ASSESSMENT OF POTENTIAL HY'S LAW CASES

The WCTU safety team will discuss with the CI and advise on follow-up procedure for all reported cases.

6. ACTIONS REQUIRED WHEN POTENTIAL HY'S LAW CRITERIA ARE MET BEFORE AND AFTER STARTING STUDY TREATMENT

This section is applicable to patients who meet PHL criteria on study treatment having previously met PHL criteria at a study visit prior to starting study treatment.

At the first on study treatment occurrence of PHL criteria being met the Investigator will:

- Determine if there has been a significant change[#] in the patients' condition compared with the last visit where PHL criteria were met
  - If there is no significant change no action is required
  - If there is a significant change notify the central Study Team, then follow the subsequent process described is Section 4.2 of this Appendix

# A 'significant' change in the patient's condition refers to a clinically relevant change in any of the individual liver biochemistry parameters (ALT, AST or total bilirubin) in isolation or in combination, or a clinically relevant change in associated symptoms. The determination of whether there has been a significant change will be at the discretion of the Investigator, this may be in consultation with the Study Physician if there is any uncertainty.

7. ACTIONS REQUIRED FOR REPEAT EPISODES OF POTENTIAL HY'S LAW

This section is applicable when a patient meets PHL criteria on study treatment and has already met PHL criteria at a previous on study treatment visit. The requirement to conduct follow-up, review and assessment of a repeat occurrence(s) of PHL is based on the nature of the alternative cause identified for the previous occurrence. The investigator should determine the cause for the previous occurrence of PHL criteria being met and answer the following question:

- Was the alternative cause for the previous occurrence of PHL criteria being met chronic or progressing malignant disease or did the patient meet PHL criteria prior to starting study treatment and at their first on study treatment visit as described in Section 6?

If No: follow the process described in Section 4.2 of this Appendix
If Yes: Determine if there has been a significant change in the patient's condition# compared with when PHL criteria were previously met

- If there is no significant change no action is required
- If there is a significant change follow the process described in Section 4.2 of this Appendix

# A 'significant' change in the patient's condition refers to a clinically relevant change in any of the individual liver biochemistry parameters (ALT, AST or total bilirubin) in isolation or in combination, or a clinically relevant change in associated symptoms. The determination of whether there has been a significant change will be at the discretion of the Investigator, this may be in consultation with the Study Physician if there is any uncertainty.

8. REFERENCES

FDA Guidance for Industry (issued July 2009) 'Drug-induced liver injury: Premarketing clinical evaluation': http://www.fda.gov/downloads/Drugs/GuidanceComplianceRegulatoryInformation/Guidances/UCM174090.pdf

Appendix 6: A guide to performing causality assessments

The following factors should be considered when deciding if there is a "reasonable possibility" that an SAE may have been caused by the drug.

- Time course. Exposure to suspect drug. Has the subject actually received the suspect drug? Did the SAE occur in a reasonable temporal relationship to the administration of the suspect drug?

- Consistency with known drug profile. Was the AE consistent with the previous knowledge of the suspect drug (pharmacology and toxicology) or drugs of the same pharmacological class? Or could the SAE be anticipated from its pharmacological properties?

- Dechallenge experience. Did the SAE resolve or improve on stopping or reducing the dose of the suspect drug?

- No alternative cause. The SAE cannot be reasonably explained by another aetiology such as the underlying disease, other drugs, other host or environmental factors.

- Laboratory tests. A specific laboratory investigation (if performed) has confirmed the relationship?

A "reasonable possibility" could be considered to exist for an SAE where one or more of these factors exist.

In contrast there would not be a "reasonable possibility" of causality if none of the above criteria apply, or where there is evidence of exposure and a reasonable time course, but any dechallenge (if performed) is negative or ambiguous or there is another more likely cause of the SAE.

In difficult cases other factors should be considered such as:
- Is this a recognised feature of overdose of the drug?
- Is there a known mechanism?

Ambiguous cases should be considered as being a "reasonable possibility" of causal relationship unless further evidence becomes available to refute this. Causal relationship in cases where the disease under study has deteriorated due to lack of effect should be classified as no reasonable possibility.

[redacted]
